# A new alligatoroid from the Eocene of Vietnam highlights an extinct Asian clade independent from extant *Alligator sinensis*

**DOI:** 10.7717/peerj.7562

**Published:** 2019-11-05

**Authors:** Tobias Massonne, Davit Vasilyan, Márton Rabi, Madelaine Böhme

**Affiliations:** 1 Department of Geosciences, Eberhard-Karls-Universität Tübingen, Tübingen, Germany; 2 Senckenberg Center for Human Evolution and Palaeoecology, Tuebingen, Germany; 3 JURASSICA Museum, Porrentruy, Switzerland; 4 Department of Geosciences, University of Fribourg, Fribourg, Switzerland; 5 Central Natural Science Collections, Martin-Luther University Halle-Wittenberg, Halle (Saale), Germany

**Keywords:** Eocene, Crocodylia, Asia, Na Duong, Phylogeny, Vietnam, *Alligator*

## Abstract

During systematic paleontological surveys in the Na Duong Basin in North Vietnam between 2009 and 2012, well-preserved fossilized cranial and postcranial remains belonging to at least 29 individuals of a middle to late Eocene (late Bartonian to Priabonian age (39–35 Ma)) alligatoroid were collected. Comparative anatomical study of the material warrants the diagnosis of a new taxon, *Orientalosuchus naduongensis* gen. et sp. nov. The combined presence of an enlarged fifth maxillary tooth, prominent preorbital ridges, a large supraoccipital exposure on the skull table, a palatine-pterygoid suture anterior to the posterior end of the suborbital fenestra, and a pterygoid forming a neck surrounding the choana is unique to this species. Unlike previous phylogenies, our parsimony analysis recovers a monophyletic Late Cretaceous to Paleogene East to Southeastern Asian alligatoroid group, here named Orientalosuchina. The group includes *Orientalosuchus naduongensis*, *Krabisuchus siamogallicus*, *Eoalligator chunyii*, *Jiangxisuchus nankangensis* and *Protoalligator huiningensis*, all of them sharing a medial shifted quadrate foramen aerum. The recognition of this clade indicates at least two separate dispersal events from North America to Asia: one during the Late Cretaceous by Orientalosuchina and one by the ancestor of *Alligator sinensis* during the Paleogene or Neogene, the timing of which is poorly constrained.

## Introduction

Alligatoroidea is a monophyletic group of Crocodylia that includes extant North American/Asian *Alligator* spp. and Central to Middle American caimans, as well as many fossil taxa (Brochu, 1999, 2004; Scheyer et al., 2013) and is defined as a stem-based group including living alligators and caimans and all taxa closer to them than to *Crocodylus* or *Gavialis* (Brochu, 1999). The fossil record points to the Late Cretaceous of North America as the time and place of origin, with subsequent dispersals to South America, Europe, and Asia, but the timing, mode, and the number of dispersals are poorly constrained (Brochu, 1999, 2004, 2010; Bona & Barrios, 2015). Europe may have been colonized multiple times by alligatoroids (Brochu, 2004), but the phylogeny is in a state of flux and key European taxa are in need of re-description.

Alligatoroids are now extinct in Europe, but they still survive in Asia with the Chinese alligator, *Alligator sinensis* Fauvel, 1879. Until now, no fossils from the Paleogene were placed on the stem-lineage of *Alligator sinensis*. Previous studies found the Paleogene East to Southeastern Asian alligatoroids *Krabisuchus siamogallicus* Martin & Lauprasert, 2010 from Thailand, *Protoalligator huiningensis* Young, 1982, *Eoalligator chunyii* Young, 1964 and the “Maoming alligatoroid” from China phylogenetically outside *Alligator* and mostly unresolved relative to other alligatoroids (Martin & Lauprasert, 2010; Skutschas et al., 2014; Wang, Sullivan & Liu, 2016; Wu, Li & Wang, 2018). Recently, *Eoalligator chunyii* was recovered as a basal member of Crocodylia together with *Jiangxisuchus nankangensis* (Li, Wu & Rufolo, 2019). The oldest record of the *Alligator sinensis* lineage has been reported from the Pliocene of Japan (Iijima, Takahashi & Kobayashi, 2016). The early Miocene *Alligator luicus* from China (Li & Wang, 1987; Brochu, 1999) was never included in a phylogenetic analysis and its relationships with extant *Alligator* species therefore remain uncertain. The ancestor of *Alligator sinensis* is nevertheless expected to be present in the Paleogene of Eastern Asia, since recent molecular clock analyses estimate its divergence from North American *Alligator mississippiensis* (Daudin, 1802) in the Paleocene or Eocene—during times of favorable climatic conditions for crocodylians crossing the Bering Strait (Wu et al., 2003; Roos, Aggarwal & Janke, 2007; Oaks, 2011). The arrival of *Alligator sinensis* from Europe is not supported by previous phylogenies, although European taxa are in need of revision. On the other hand, a post-Eocene dispersal via Beringia would have been problematic because of the low tolerance for cool climate of crocodylians (Markwick, 1998). *Alligator sinensis* therefore represents a biogeographic enigma, so the East and Southeastern Asian Paleogene fossil record is critical for resolving this issue. However, all previously described fossils from this continent are highly incomplete, which hinders a rigorous test of phylogenetic relationships.

Most of the Paleogene fossil record of Crocodylia comes from North America and Europe, whereas the Asian record is still insignificant in comparison: only ca. 10% of the Paleogene sampled taxa of phylogenies are Asian (Martin & Lauprasert, 2010; Brochu, 2012; Skutschas et al., 2014; Jouve, 2016; Wang, Sullivan & Liu, 2016; Shan et al., 2017). Moreover, with one exception (Shan et al., 2017), these taxa are only known from fragmentary or deformed fossils.

During systematic surveys in the Na Duong Basin in Northern Vietnam between 2009 and 2012, 29 well preserved individuals of an Eocene alligatoroid were collected and subsequently prepared by the Geological-Paleontological Institute of the Eberhard Karls University of Tübingen (GPIT) (Böhme et al., 2012).

In this study, we provide a complete description of this material, which represents the best preserved alligtatorid from the Paleogene of Asia. We demonstrate that Cretaceous-Eocene East to Southeastern Asian alligatoroids form a monophyletic group, here named Orientalosuchina and that Asia was colonized by alligatoroids at least two times independently.

## Geological settings

**Figure 1:**
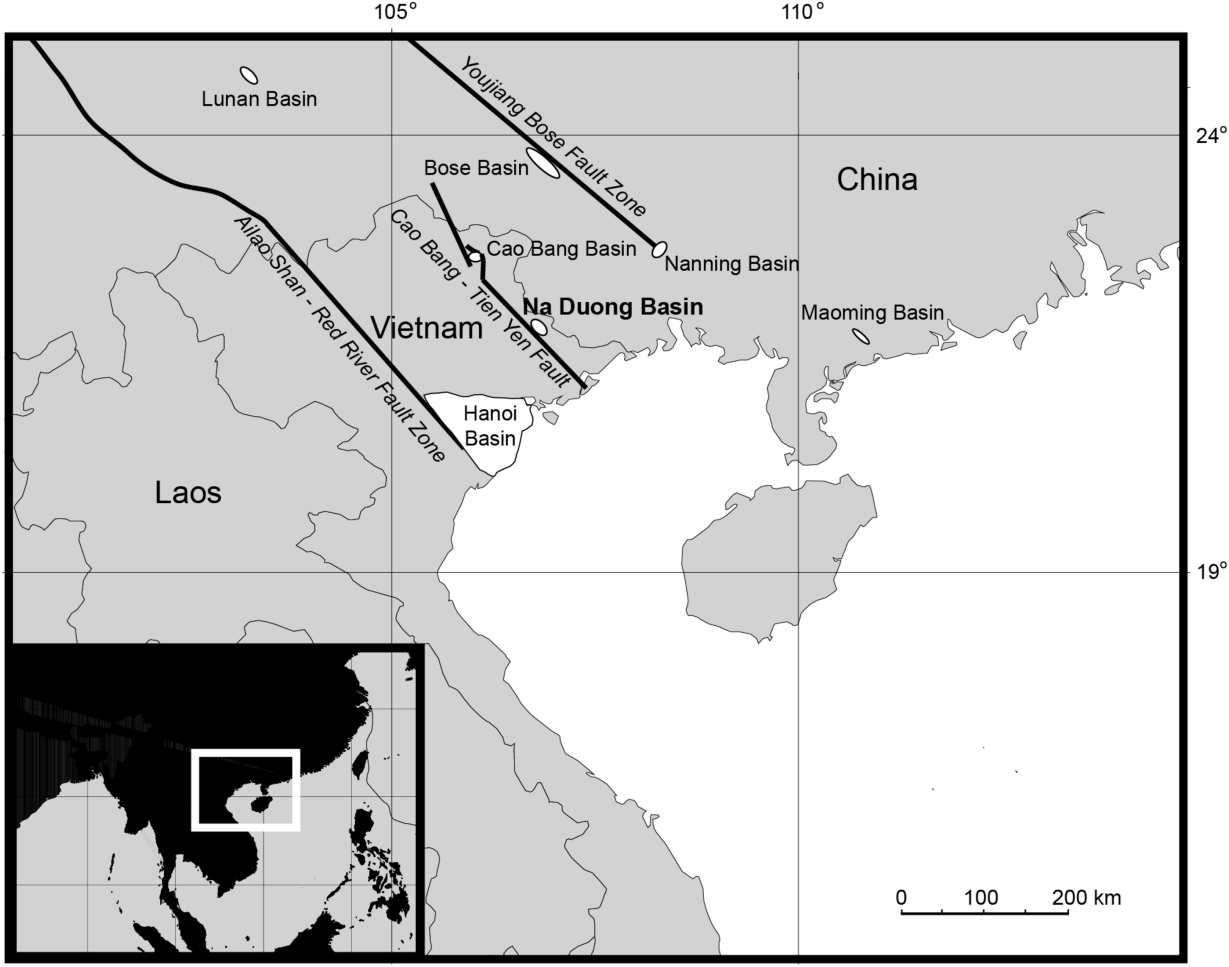
Map of northern southeastern Asia, showing the Na Duong Basin in northeastern Vietnam near the border with China (Böhme et al., 2012).

The Na Duong Basin is located in northern Vietnam near the border with China (Fig. 1). It represents one of the few areas in East and Southeastern Asia with continental sediments of Eocene to Oligocene age (Böhme et al., 2012). The pull-apart basin is part of the Cao Bang—Tien Yen fault system and covers an area of around 45 square kilometers. The middle to upper Eocene (late Bartonian-Priabonian (39–35 Ma)) Na Duong Formation is 240 m thick; its upper 140 m are part of the Na Duong open cast coal mine. The alligatoroid remains in this work were found within the transition zone between the coaly shale of the main seam and the underlying dark-brown clay-stone (layer 80), together with many other vertebrate fossils (Böhme et al., 2012).

The fossiliferous layer 80 was a tropic to warm-subtropical swamp ecosystem with aquatic and terrestrial environments. During sedimentation, the area was in a transitional stage from shallow ponds to an anoxic lake. Further, tomistomine and *Asiatosuchus*-like crocodiles, as well as many fish taxa and two different turtle species occurred sympatrically with the herein described alligatoroid (Böhme et al., 2012; Garbin, Böhme & Joyce, 2019).

## Materials and Methods

We expanded the taxon-character dataset of Brochu & Storrs (2012) (see Appendix), which was the most recent global Crocodylia matrix available during the start of this study. The expanded dataset includes 202 characters; 189 characters are from Brochu & Storrs (2012), two characters from Wang, Sullivan & Liu (2016), one character from Cossette & Brochu (2018), one character from Jouve (2004), and nine new characters from the present study. We further modified characters (51) and (91) of Brochu & Storrs (2012), (190) of Wang, Sullivan & Liu (2016) and (174) of Jouve et al. (2008) (195 in this study). In total, we included 114 taxa: in addition to the 103 taxa added from Narváez et al. (2015), *Globidentosuchus brachyrostris* Scheyer et al., 2013, *Culebrasuchus mesoamericanus* Hastings et al., 2013 and *Centenariosuchus gilmorei* Hastings et al., 2013 from Hastings, Reisser & Scheyer, 2016, the Maoming alligatoroid, *K. siamogallicus*, *Protoalligator huiningensis*, *Eoalligator chunyii* and *Asiatosuchus nanlingensis* Young, 1964 from Wang, Sullivan & Liu (2016), *Bottosaurus harlani* from Cossette & Brochu (2018), *J. nankangensis* from Li, Wu & Rufolo (2019) and the herein described *Orientalosuchus naduongensis*. For *Orientalosuchus naduongensis*, we could score 118 characters (the complete data set is found in File S1). Character scorings were modified for 32 taxa in total (a complete list of changes together with the specimen list can be found in File S2). We provided the complete character list in File S3.

We conducted a maximum parsimony analysis in TNT 1.5 standard version updated on November 20, 2018 (Goloboff, Farris & Nixon, 2008). We treated the multistate characters as unordered and equally weighted; set the maximum of trees to 10,000, and the tree replications to 1,000. For swapping algorithm, we used tree bisection reconnection with 10 trees saved per replication.

A first run of heuristic search tree-bisection-reconnection, failed to find all the most parsimonious trees (MPT) and, therefore, the heuristic search was repeated until the MPTs were found 50 times during each replicate (using the command “xmult = hits 50;”). The trees retained in the memory were exposed to a second round of tree-bisection-reconnection.

The electronic version of this article in portable document format will represent a published work according to the International Commission on Zoological Nomenclature (ICZN), and hence the new names contained in the electronic version are effectively published under that Code from the electronic edition alone. This published work and the nomenclatural acts it contains have been registered in ZooBank, the online registration system for the ICZN. The ZooBank Life Science Identifiers (LSIDs) can be resolved and the associated information viewed through any standard web browser by appending the LSID to the prefix http://zoobank.org/. The LSID for this publication is: urn:lsid:zoobank.org:pub:08B6F167-AAC7-4184-97BA-B7467D4F036B. The online version of this work is archived and available from the following digital repositories: PeerJ, PubMed Central and CLOCKSS.

## Systematic Paleontology

**Figure 2:**
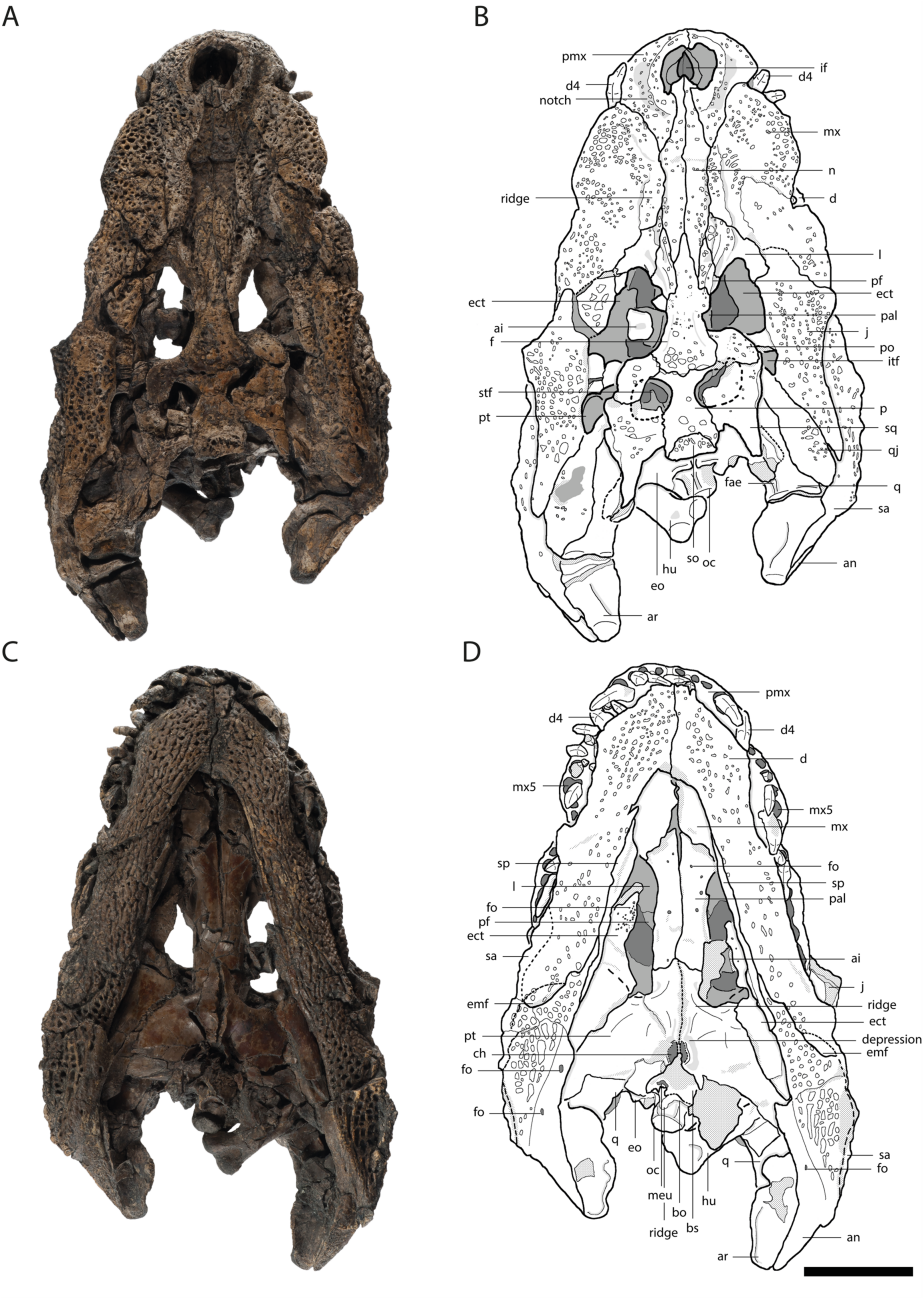
Skull of *Orientalosuchus naduongensis* (GPIT/RE/09761) (holotype), Na Duong Formation, upper Eocene, Vietnam. Skull in dorsal (A and B) and ventral (C and D) view. Abbreviations: ai, atlas intercentrum; an, angular; ar, articular; bo, basioccipital; bs, basisphenoid; ch, choana; d, dentary; d4, dentary tooth 4; emf, external mandibular fenestra; eo, exoccipital; ect, ectopterygoid; f, frontal; fo, foramen; fae, foramen aerum; hu, humerus; if, incisive foramen; itf, infratemporal fenestra; j, jugal; l, lacrimal; mx, maxilla; mx5, maxilla tooth 5; n, nasal; oc, occipital condylus; p, parietal; pf, prefrontal; pal, palatine; pmx, premaxilla; po, postorbital; pt, pterygoid; q, quadratum; qj, quadratojugal; sa, surangular; so, supraoccipital; sp, splenial; sq, squamosal; stf, supratemporal fenestra. Scale = 5 cm.

**Eusuchia** Huxley, 1875 sensu Brochu, 2003
**Crocodylia** Gmelin, 1789 sensu Benton & Clark, 1988
**Alligatoroidea** Gray, 1844 sensu Brochu, 2003
**Globidonta** Brochu, 1999
**Alligatoridae** Cuvier, 1807 sensu Brochu, 2003
**Orientalosuchina new clade name**
***Orientalosuchus* gen. nov.**
*Orientalosuchus naduongensis* sp. nov.
(Fig. 2)

### Orientalosuchus

Etymology: The name *Orientalosuchus* refers to the Latin word “oriens” for “east” and “suchus” the old Greek word “soukhos” for “crocodile.”

### Orientalosuchus naduongensis

Etymology: The species name “*naduongensis*” refers to the Na Duong coal mine type locality in northeastern Vietnam.

Diagnosis: *Orientalosuchus naduongensis* is diagnosed by the combination of the following characters: notch between the premaxilla and maxilla; dominant maxillary ridge alongside the nasal; the fifth maxillary tooth is the largest maxillary tooth; anterior tip of frontal is acute and projects between the nasal bones; small supratemporal fenestra; large supraoccipital exposure preventing the parietal from reaching the posterior skull table in adults; quadrate foramen aerum lies on the dorsomedial angle of the quadrate; large suborbital fenestrae reaching anteriorly the level of the seventh to eighth maxillary tooth; maxilla-palatine suture forms an obtuse angle and not reaching beyond the anterior end of the suborbital fenestra; palatine-pterygoid suture lies anterior to the posterior end of the suborbital fenestra; pterygoid forms a neck surrounding the choana; dentary tooth row with only 16 teeth; laterally compressed posterior teeth; very small external mandibular fenestra; foramen aerum at the lingual margin of the retroarticular process; axis with a hypapophysis that is located near the center of the centrum; coracoid with a very large glenoid; iliac blade with a rectangular posterior outline and a dorsal indentation; dorsal osteoderms with no or only modest ridge.

Differential diagnosis: *Orientalosuchus naduongensis* differs from *Krabisuchus siamogallicus* in having dorsal osteoderms with no or only a modest ridge; an inward-pushed pterygoid around the choana and a neck surrounding the aperture, while the pterygoid surface is flush with the choanal margin in *K. siamogallicus*; and a very large supraoccipital exposure, preventing the parietal from reaching the posterior edge of the skull.

*Orientalosuchus naduongensis* differs from *Eoalligator chunyii* in having dorsal osteoderms with no or only a modest ridge; very prominent preorbital ridges; squamosals, that do not extend ventrolaterally to lateral extent of paroccipital process; a very large supraoccipital exposure, preventing the parietal from reaching the posterior edge of the skull; a smooth dorsal surface of the surangular, whereas *Eoalligator chunyii* has a large sulcus next to the anterior half of the glenoid fossa; and an intersupratemporal bar similarly broad as the supratemporal fenestra, while the bar is strongly constricted in *Eoalligator chunyii*.

*Orientalosuchus naduongensis* differs from *Jiangxisuchus nankangensis* in having a deeply curved dentary; a small incisive foramen; prominent preorbital ridges; a palatine-pterygoid suture nearly at the posterior angle of suborbital fenestra; a frontoparietal suture entirely on the skull table (the suture modestly enters the supratemporal fenestra in *J. nankangensis*); a very large supraoccipital exposure preventing the parietal from reaching the posterior edge of the skull; anterior maxillary teeth with vertical ridges on their lateral surface; and an intersupratemporal bar that is similarly broad as the supratemporal fenestra (the bar is constricted in *J. nankangensis*).

*Orientalosuchus naduongensis* differs from *Protoalligator huiningensis* in having a deeply curved dentary; laterally compressed posterior teeth; a deep notch lateral to the naris; an occlusion pit between the seventh and eighth maxillary teeth with all other dentary teeth occluding lingually, while in *Protoalligator huiningensis* all dentary teeth are lingual to maxillary teeth; very prominent preorbital ridges; and maxillary teeth with vertical ridges on their lateral surface.

Holotype: GPIT/RE/09761; partial skeleton consisting of skull, lower jaws and incomplete postcranial skeleton (see Table 1).

**Table 1:** List of specimens of *Orientalosuchus naduongensis*, Na Duong Formation, upper Eocene, Vietnam.

Individual	Cranial material	Postcranial material
GPIT/RE/09761 (holotype) Fig. 2 and Figs. 11–20	Complete dorsoventrally flattened skull and lower jaw fused together	Atlas intercentrum; axis; seven cervical vertebrae; 10 dorsal vertebrae; one sacral vertebra; eight caudal vertebrae; two cervical ribs; four dorsal ribs; one scapula (right); one coracoid (right); two humeri; one radiale; four metacarpalia (?); seven manus phalanges (?); two ilia; one ischium (left); two femora; one tibia (left); one fibula (right); four claws; >50 osteoderms
GPIT/RE/09730 Fig. 3	Dorsoventrally flattened anterior skull part reaching slightly behind the orbita and the complete right lower jaw ramus	–
GPIT/RE/09729 Fig. 4	Well preserved dorsoventrally flattened posterior skull part reaching to the premaxilla on the right side	–
GPIT/RE/09728 Figs. 5–8	Crushed complete skull and broken but well preserved anterior lower jaw parts	–
GPIT/RE/09727 Fig. 9	Crushed complete skull and well preserved posterior lower jaw parts	Three cervical vertebrae; 11 dorsal vertebrae; six caudal vertebrae; two dorsal ribs; one scapula (left); one humerus (right); one radius (right); two ilia; two femora; two tibiae; two fibulae; >50 osteoderms
GPIT/RE/09762	Complete skull without the most anterior part, lower jaw fragments and a few further skull fragments	–
GPIT/RE/09763	Posterior skull part	–
GPIT/RE/09764	Posterior skull part	–
GPIT/RE/09765	Anterior skull part	–
GPIT/RE/09766	Posterior and lateral skull part	–
GPIT/RE/09767	Posterior and lateral skull part	One cervical vertebra
GPIT/RE/09768	Skull and lower jaw	–
GPIT/RE/09769 Fig. 6	Half skull	Vertebra + rib + osteoderms
GPIT/RE/09770	Anterolateral skull part	bone fragments
GPIT/RE/09771	Posterior and lateral skull parts	–
GPIT/RE/09772	Posterior skull part	–
GPIT/RE/09773	Posterior skull part with fused lower jaw	–
GPIT/RE/09774	Skull with fused lower jaw	–
GPIT/RE/09775	Skull and lower jaw with the anteriormost part missing	–
GPIT/RE/09776	Skull fragments and lower jaw	–
GPIT/RE/09777	Posterior skull part	–
GPIT/RE/09778	Premaxilla	One dorsal vertebra
GPIT/RE/09779	Skull fragments	–
GPIT/RE/09780	Single small skull fragment and lower jaw fragments	–
GPIT/RE/09781	Lower jaw	–
GPIT/RE/09782	Lower jaw ramus	–
GPIT/RE/09783	Lower jaw	Bone fragments
GPIT/RE/09784	Lower jaw fragment	Seven cervical vertebrae; eight dorsal vertebrae; five caudal vertebrae; one cervical rib; four dorsal ribs; one scapula (right); two ulnae; two ilia; two ischia; one pubis (left); two femora; two tibiae; two fibulae; three tarsalia; one astragalus (left); one calcaneus (left); five metatarsalia; 14 pedal phalanges; four claws; >50 osteoderms
GPIT/RE/09785	–	Fragments

Type locality and horizon: The fossils were recovered from layer 80 of the Na Duong coal mine in northern Vietnam (N 21°42.2′, E 106°58.6′); Na Duong Formation, Eocene, late Bartonian to Priabonian age (39–35 Ma) (Böhme et al., 2012).

Referred material: A total of 29 individuals represented by incomplete skulls, skull fragments and associated postcranial material, Na Duong Formation, Na Duong coal mine, Vietnam (see a complete list of preserved specimens and their associated material is presented in Table 1).

Preservation: The material from Na Duong is mostly well preserved and nearly complete but all skulls are dorsoventrally flattened and many have deformed and crushed areas or weathered surfaces. The postcranial material is mostly disarticulated or fused together with the matrix. The majority of the bones are pyritized.

## Phylogenetic Nomenclature

### Orientalosuchina

Definition: Orientalosuchina refers to the most inclusive clade containing *Orientalosuchus naduongensis* gen. et sp. nov., *Krabisuchus siamogallicus* Martin & Lauprasert, 2010, *Eoalligator chunyii* Young, 1964, *Jiangxisuchus nankangensis* Li, Wu & Rufolo, 2019 and *Protoalligator huiningensis* Young, 1982, but not *Brachychampsa montana* Gilmore, 1911, *Stangerochampsa mccabei* Wu, Brinkman & Russell, 1996, *Leidyosuchus canadensis* Lambe, 1907, *Diplocynodon darwini* (Ludwig, 1877), *Bottosaurus harlani* Von Meyer, 1832, or any species of recent Crocodylia.

## Description

### Cranial description

Measurements of the cranial material are presented in Table. 2. Unless otherwise stated, the description is based on the holotype (GPIT/RE/09761) (Fig. 2).

**Table 2:** Cranial measurements of *Orientalosuchus naduongensis*, Na Duong Formation, upper Eocene, Vietnam.

	GPIT/RE/09731 (Holotype)	GPIT/RE/09728	GPIT/RE/09729	GPIT/RE/09730
Skull length (premaxilla-supraoccipital)	190.5	185.1*	?	?
Skull width (quadratojugal-quadratojugal)	127.1	135.8	82.6*	?
Preorbital length	104.5	101.8*	?	64.4
Skull table length	43.6	49.3	34.6	?
Skull table width	65.2*	65.9	46.2	49.8
External naris length	21.1	10.8*	12.8	10.1*
External naris width	22.5	24.2	10.2*	17.8*
Orbita length	41.1	42.6*	33.3	28.6
Orbita width	26.8*	27.5	17.3*	19.7
Supratemporal fenestra length	12.3*	19.4	13.6	?
Supratemporal fenestra width	13.7	15.4	8.7	?
Infratemporal fenestra length	11.4	19.2*	9.1	?
Infratemporal fenestra height	?	?	11.5*	9.0*
Suborbital fenestra length	63.7*	?	49.9	43.6
Suborbital fenestra width	?	?	17.6*	?
Width between orbits	15.8	15.9	10.3	10.3
Width between supratemporal fenestrae	12.1	11.8	11.9	?
Width between suborbital fenestrae	22.7	?	13.6*	17.4
Occipital condyle height	9.5	9.7*	?	?
Occipital condyle width	14.4	12.8*	9.3	?

**Note:**

All measurements in mm (*measurements on deformed sections).

### Premaxilla

**Figure 3:**
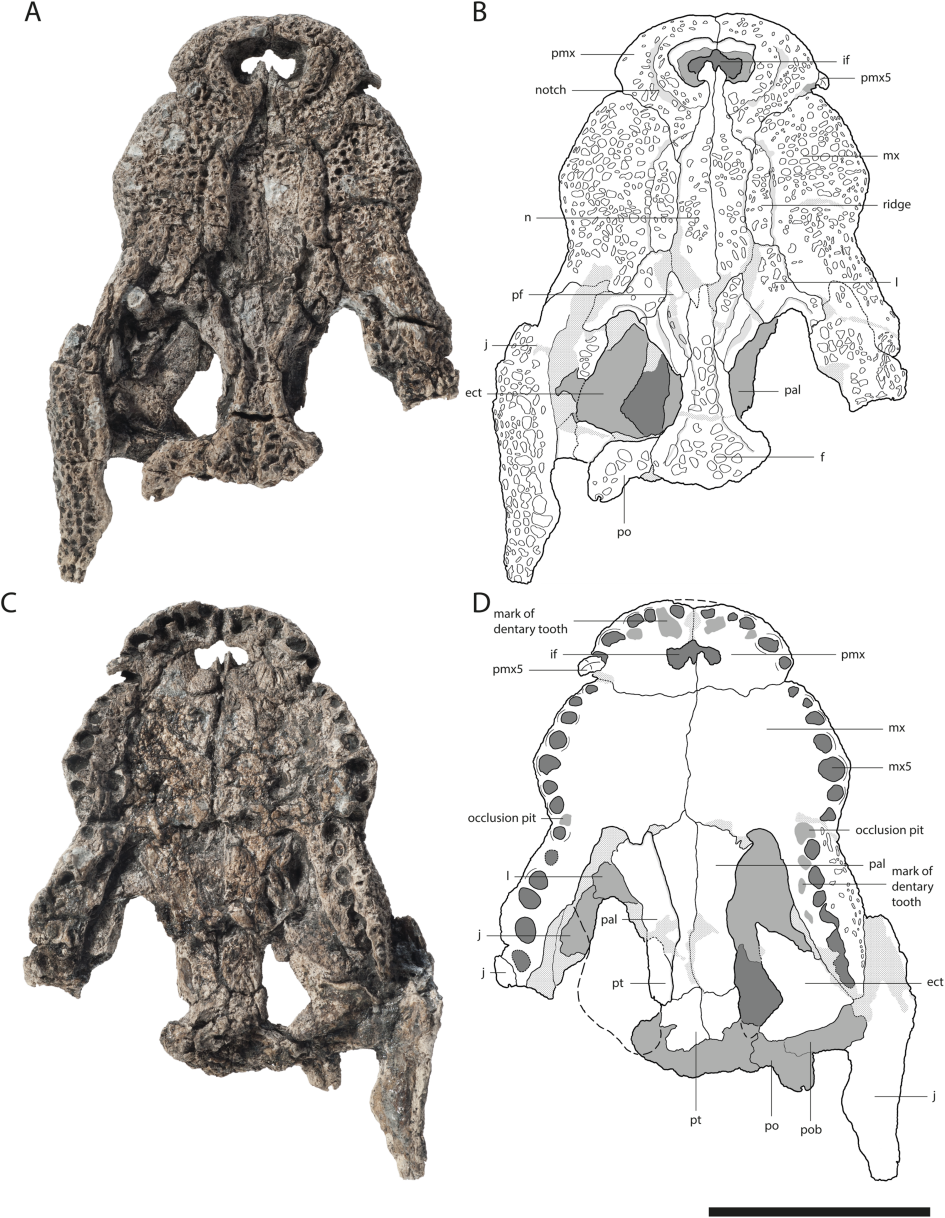
Skull of *Orientalosuchus naduongensis* (GPIT/RE/09730), Na Duong Formation, upper Eocene, Vietnam. Skull in dorsal (A and B) and ventral (C and D) view. Abbreviations: ect, ectopterygoid; f, frontal; if, incisive foramen; j, jugal; l, lacrimal; mx, maxilla; mx5, maxilla tooth 5; n, nasal; pal, palatine; pf, prefrontal; pmx, premaxilla; pmx5, premaxilla tooth 5; po, postorbital; pob, postorbital bar; pt, pterygoid. Scale = 5 cm.

The premaxilla in ventral view is best observable in GPIT/RE/09730 (Fig. 3). It has five teeth in total. Between the first and second tooth there is a large occlusion pit for the first dentary tooth. The teeth increase in size posteriorly. The first two teeth are very small with the third one nearly double their size. The fourth tooth is the largest one. The fifth tooth is again much smaller, but still larger than the first two.

The dorsal surface is best preserved in GPIT/RE/09761 (Fig. 2) and GPIT/RE/09730 (Fig. 3). It is ornamented with multiple small pits. The premaxilla surrounds the naris with a prominent anterolateral bulge, but it does not possess a crest. Lateral to this bulge, the premaxilla has a deep depression. The anterior margin of the naris has a short (roughly one third of the naris length) posteriorly reaching process formed by the premaxilla. The naris opening itself has a roughly square-shaped to round outline. The naris is dorsally oriented. The relatively small oval incisive foramen (Fig. 3) does not abut the tooth row.

Laterally, the premaxilla-maxilla suture originates in a notch for the enlarged fourth dentary tooth and terminates in a long premaxillary process, which extends between the nasal and the maxilla up to the level of the fourth maxillary tooth. The lateral origin of the suture lies shortly behind the level of the posterior part of the naris. The deep notch for receiving the fourth dentary tooth is present in all the large and presumably adult individuals; in the preserved juveniles, this region is damaged, making it impossible to document the condition early in ontogeny.

In ventral view, the premaxilla-maxilla suture is somewhat obscure, but seems to extend relatively straight lateromedially along the level of the notch (Fig. 3).

The premaxilla-nasal suture originates at the posterior end of the naris and flares lateromedially toward the posterior process of the dorsal plate of the premaxilla.

### Maxilla

The maxillary tooth row comprises 13 teeth. The first maxillary tooth is about the same size as the fifth premaxillary tooth. They increase in size until reaching the fifth maxillary tooth, which is the largest one (Fig. 3). Between the seventh and eighth maxillary tooth there is a complete interfingering of a dentary tooth (most likely the 11th). The fourth dentary tooth fits in the notch between the premaxilla and maxilla. The posterior part of the dentary tooth row lies completely lingually to the maxillary tooth row, except for the presumably 11th dentary tooth, indicated by marks of the posterior dentary teeth lingual to the maxillary teeth, best visible in GPIT/RE/09730 (Fig. 3).

The lateral outline of the maxilla is considerably curved. In dorsal view, the bone flares laterally until reaching the level of the fifth maxillary tooth, which marks the most convex point of the snout. From there it tapers medially up to the level of the constriction between the seventh and eighth teeth and flares further posterior towards the suture with the jugal.

**Figure 4:**
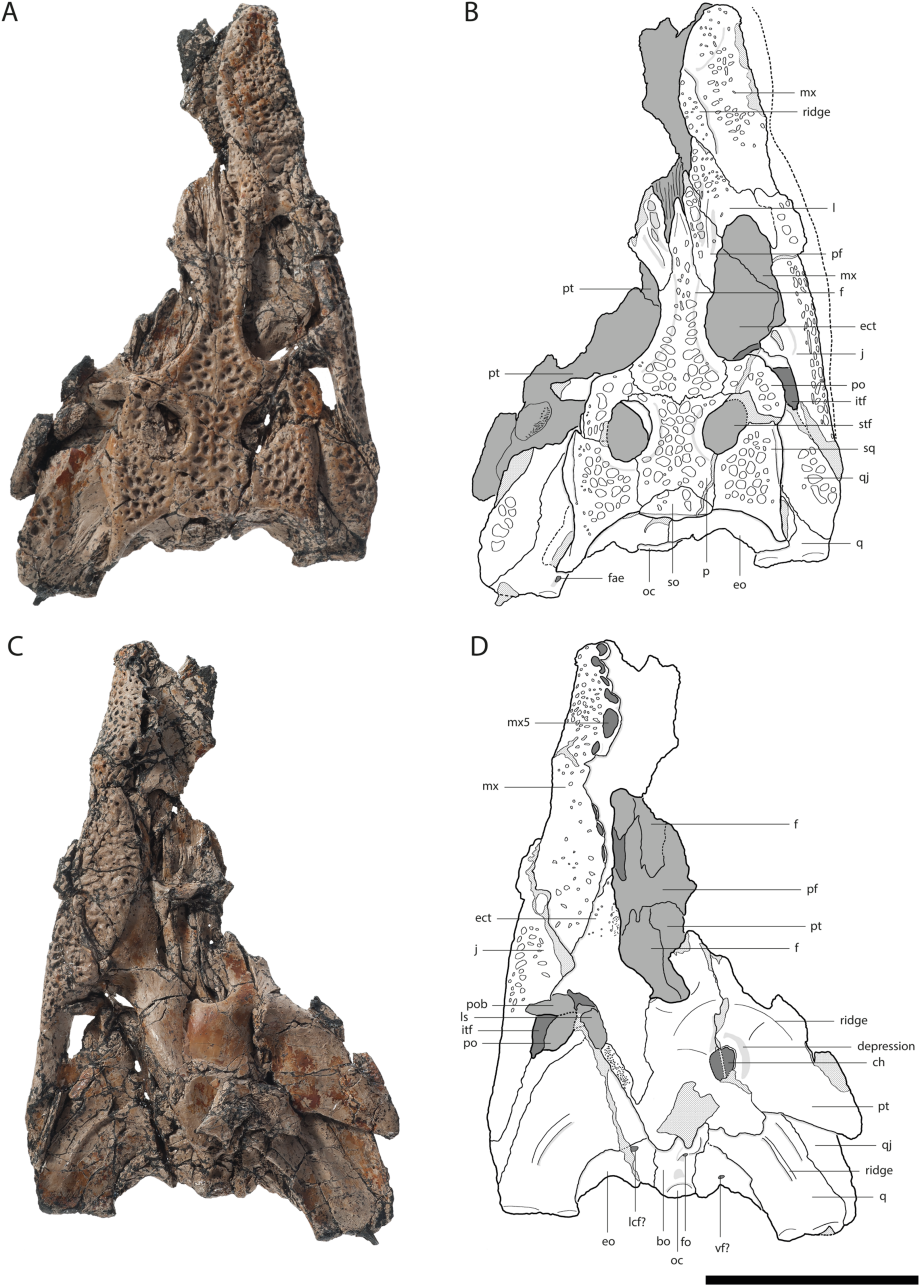
Skull of *Orientalosuchus naduongensis* (GPIT/RE/09729), Na Duong Formation, upper Eocene, Vietnam. Skull in dorsal (A and B) and ventral (C and D) view. Abbreviations: bo, basioccipital; ch, choana; ect, ectopterygoid; f, frontal; fae, foramen aerum; fo, foramen; itf, infratemporal fenestra; j, jugal; l, lacrimal; lcf, lateral carotid foramen; ls, laterosphenoid; mx, maxilla; mx5, maxilla tooth 5; oc, occipital condylus; p, parietal; pf, prefrontal; po, postorbital; pob, postorbital bar; pt, pterygoid; q, quadrate; qj, quadratojugal; so, supraoccipital; sq, squamosum; stf, supratemporal fenestra; vf, vagus foramen. Scale = 5 cm.

**Figure 5:**
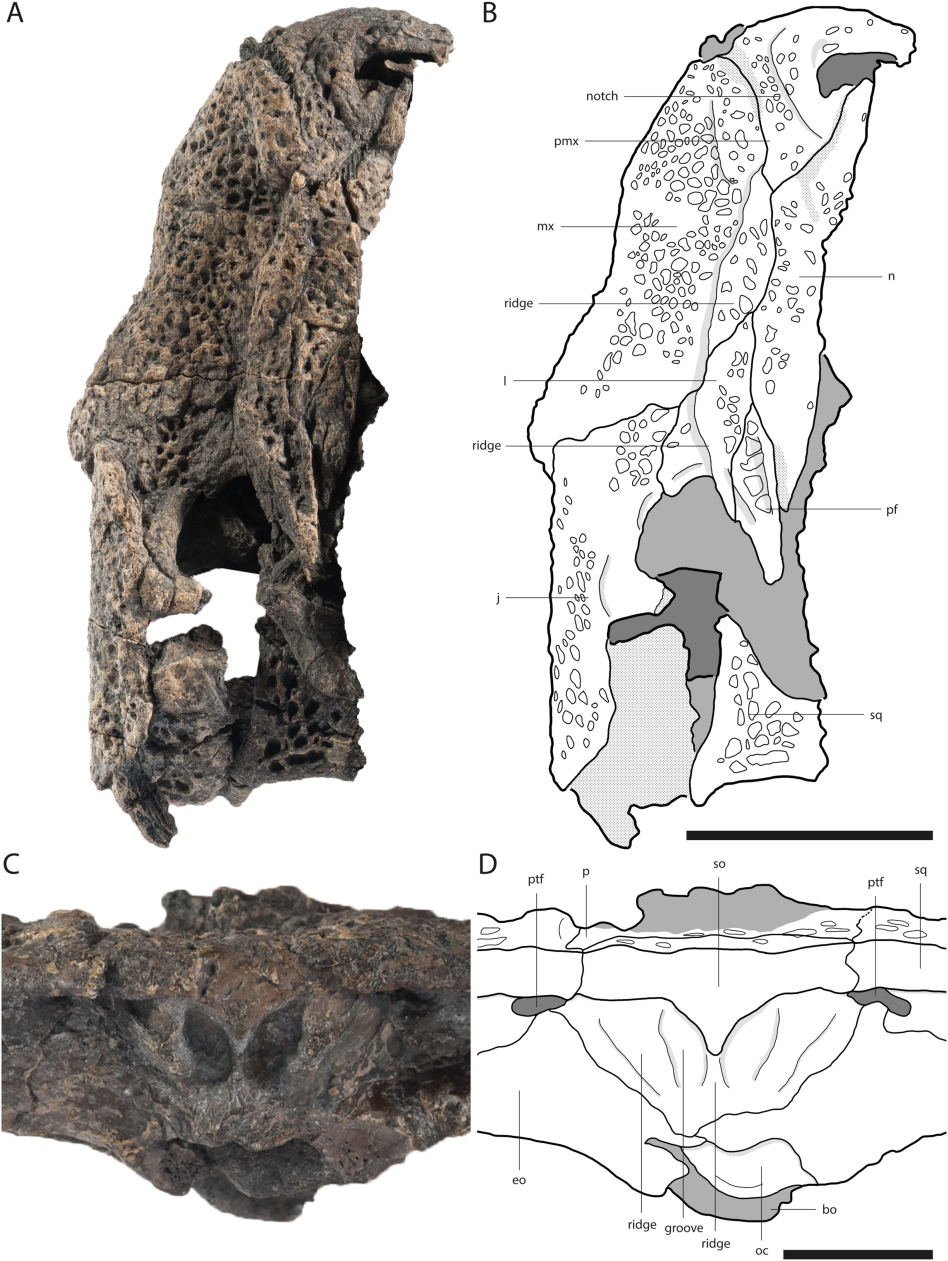
Skull of *Orientalosuchus naduongensis* (GPIT/RE/09728), Na Duong Formation, upper Eocene, Vietnam. Skull in dorsal (A and B) and occipital (C and D) view. Abbreviations: bo, basioccipital; eo, exoccipital; j, jugal; l, lacrimal; mx, maxilla; n, nasal; oc, occipital condylus; p, parietal; pf, prefrontal; pmx, premaxilla; ptf, posterior temporal fenestra; so, supraoccipital; sq, squamosum. Scale = 5 cm (A and B). Scale = 1 cm (C and D).

The dorsal surface of the maxilla is densely ornamented and has a strictly anteroposteriorly oriented prominent ridge alongside the nasal bone (best observable in GPIT/RE/09730 Fig. 3, GPIT/RE/09729 Fig. 4 and GPIT/RE/09728 Fig. 5). The dorsal surface of the ridge is rounded and becomes flatter anteriorly and terminates at the posteriormost part of the premaxillary process. Posteriorly, the ridge continues across the lacrimal and prefrontal until reaching the anteromedial part of the orbit. Laterally to the ridge, there is an elongated groove. The maxilla-premaxilla suture extends posteromedially until reaching the nasal. Slightly posteriorly to the suture, a shallow groove is present. The maxilla-nasal suture extends straight anteroposteriorly and is relatively short due to the long premaxilla process and the far anteriorly reaching lacrimal. The suture between the maxilla and lacrimal projects posterolaterally and then projects laterally in front of the orbit. The suture with the jugal is somewhat obscure, but seems to extend laterally until it becomes straight when it extends posteriorly (best observable in GPIT/RE/09729 Fig. 4).

In ventral view, the suture between the maxilla and palatine forms an obtuse angle with the anteriormost tip of the palatine situated at the level of the anterior end of the suborbital fenestra. The suture extends posterolaterally until reaching the anteromedial border of the fenestra. The small maxillary foramen for the palatine ramus of the cranial nerve V is visible in a single individual (GPIT/RE/09770) medial to the fifth tooth of the maxilla.

The maxilla-ectopterygoid suture is shifted posterolaterally from the tooth row, preventing the ectopterygoid from contacting the alveoli (best seen in GPIT/RE/09730 Fig. 3).

### Nasal

The elongated nasal is around four-times longer anteroposteriorly than lateromedially wide and similarly ornamented as the premaxilla and maxilla. The bone seems recessed compared to the paired maxillary ridges in GPIT/RE/09761 (Fig. 2) and GPIT/RE/09730 (Fig. 3), however, this is an artifact due to the postmortem deformation of the skull. GPIT/RE/09728 (Fig. 5) reveals, that the nasal was at the same height as the maxilla ridges, giving them a rim-like outline.

Anteriorly, the nasal projects into the naris with a short process for around one-third of the naris length, but it is unclear how complete this septum in the intact skull was. Therefore it remains unclear whether the naris was bisected. A complete or near-complete bisection would be consistent with the midline posterior process of the premaxilla but only better preserved specimens will help resolving this. The nasal-lacrimal suture is posteriorly oriented. The suture between the nasal and prefrontal slopes slightly posteromedially until reaching the frontal. Posteriorly, the nasal sends a long process between the frontal and prefrontal.

### Lacrimal

The general outline of the lacrimal is roughly triangular with a concavity around the lacrimal-maxilla suture, leading to a relatively slender appearance of the bone, best visible in GPIT/RE/09730 (Fig. 3) and GPIT/RE/09729 (Fig. 4). The ornamentation is overall weak but it is pronounced near the lacrimal-nasal suture. The medial part of the lacrimal is strongly elevated as the maxilla ridge proceeds toward the orbit, which is best observable in GPIT/RE/09728 (Fig. 5).

The lacrimal-prefrontal suture originates at the anteromedial part of the orbit and extends anteromedially until reaching the nasal. Posteriorly, the lacrimal covers the anterior part of the orbit. The naso-lacrimal duct is visible on the posteromedial end of the bone near the suture with the prefrontal. The contact between the lacrimal and jugal projects nearly straight posteriorly from the anterior end of the orbit.

### Prefrontal

The prefrontal is roughly wedge-shaped. Its central region is highly elevated due to the posteriorly projecting ridge, which extends roughly to the posterior part of the bone. Medially to the ridge, the prefrontal has an anteroposterior-oriented row of deep pits, especially visible in GPIT/RE/09729 (Fig. 4) and GPIT/RE/09728 (Fig. 5). The suture between the prefrontal and frontal extends anteroposteriorly and is relatively short due to the far posteriorly reaching nasal.

### Frontal

The frontal is roughly wedge-shaped with an elongated anterior process projecting between the two nasal bones. It forms the dorsomedial border of the orbit. The border itself is nearly flush with the orbital margin (GPIT/RE/09729 Fig. 4) or only very slightly upturned (GPIT/RE/09730 Fig. 3). The orbit is nearly oval and slightly constricted anteromedially.

The anterior region of the frontal has nearly no ornamentation, whereas large pits are present between the orbits posteriorly. Between the orbits, the pits are roughly aligned in a pair of rows and dissolve in a field of large pits posteriorly (best observable in GPIT/RE/09729 Fig. 4).

The suture with the parietal is oriented entirely on the skull table and has a small posteriorly reaching medial process, best visible in GPIT/RE/09761 (Fig. 2) and GPIT/RE/09729 (Fig. 4). The suture with the postorbital originates near the posteromedial border of the orbit and slopes afterwards very slightly posteromedially until reaching the parietal.

### Postorbital

The bone is best observable in GPIT/RE/09729 (Fig. 4). It is nearly boomerang-shaped and forms the anterolateral part of the skull table. The ornamentation is roughly arranged in a single line in the center of the bone and is composed of relatively large pits. The anterior part of the postorbital forms the posterior margin of the orbit, whereas its posterior region forms the anterolateral margin of the supratemporal fenestra. The slender postorbital bar is inset from the skull table and shapes the anterior part of the nearly triangular infratemporal fenestra.

The postorbital-parietal suture originates at the anteriormost point of the supratemporal fenestra and extends anteriorly only for a very short section until projecting straight medially and reaching the frontal. The suture between the postorbital and squamosal begins roughly at the level of the last third to the mid point of the supratemporal fenestra and projects laterally, until reaching the skull table and then it becomes obscure. The suture between the postorbital and jugal on the postorbital bar cannot be clearly followed.

### Parietal

The parietal is best observable in GPIT/RE/09729 (Fig. 4). It is roughly rectangular and densely ornamented with deep pits. It forms the anteromedial and medial walls of the supratemporal fenestra. The supratemporal fenestrae are oval and open. They are relatively small and located far away from the posterior border of the skull table, leading to a very long parietal-squamosal suture, which originates at the posteromedial margin of the supratemporal fenestra and projects straight toward the posterior edge of the skull. Posteriorly, the parietal does not reach the skull table in adults because of the large trapezoid supraoccipital.

### Squamosal

The squamosal forms the posterolateral margin of the skull table and the posterolateral margin of the supratemporal fenestra and is best observable in GPIT/RE/09729 (Fig. 4). Its surface is richly ornamented with deep pits. The dorsal and ventral rims of the squamosal groove for the external ear valve musculature are parallel. Due to the dorsoventral crushing of all individuals, the otic aperture is not preserved.

Posterolaterally, the squamosal has an elongated process projecting dorsally towards the paroccipital process. The most posterolateral part is not well preserved, but the squamosal does extend ventrolaterally to the lateral extent of the paroccipital process. The suture between the squamosal and exoccipital origins ventrolaterally from the posttemporal fenestra and extends ventrolaterally. Due to the crushing, the suture between the squamosal and quadrate is obscure.

### Jugal

The jugal is best observable in GPIT/RE/09761 (Fig. 2). It covers the lateral part of the skull contacting the maxilla and lacrimal anteriorly, the quadratojugal posteriorly and the postorbital on the postorbital bar dorsomedially. Its posterodorsal surface is highly ornamented with larger pits (GPIT/RE/09730 Fig. 3).

Medially, the jugal forms the ventrolateral border of the orbit and infratemporal fenestra. The border with the orbit is nearly straight, only curving slightly laterally, whereas at the border with the infratemporal fenestra, the jugal seems slightly more concave in outline. The postorbital bar is not flush with the rest of the jugal, but inserted from it medially. At the height of the postorbital bar, the posteroventral part of the jugal is strongly concave.

The jugal forms a straight suture with the quadratojugal bone, which slopes posterolaterally. The suture seems to originate from the posterolateral corner of the infratemporal fenestra (Figs. 2 and 4), but the preservation is insufficient to state this with confidence.

### Quadratojugal

The quadratojugal surface is mainly smooth, but ornamented with a few large pits near the suture with the jugal.

The bone forms the posterolateral part of the skull and the posterior border of the infratemporal fenestra. The border is smooth and does not possess a spine. The quadratojugal seems to cover the whole border preventing the quadrate from reaching the postorbital, as seen in GPIT/RE/09761 (Fig. 2) and GPIT/RE/09729 (Fig. 4). Posteromedially, the bone is very broad and rounded. Posterolaterally, it nearly reaches the most posterior part of the skull, only slightly anterior to the quadrate.

### Quadrate

The condyles lay on a horizontal axis, which is slightly inclined ventromedially with the lateral condyle larger than the medial one. The medial condyle bears a notch for the foramen aerum on its dorsomedial border. The relatively small foramen aerum is visible in GPIT/RE/09761 (Fig. 2) GPIT/RE/09729 (Fig. 4) and GPIT/RE/09728 and lies on the dorsomedial surface of the medial condyle. The opening of the cranioquadrate canal and the otic area are crushed. On the ventral surface of the bone, a prominent crest of the posterior mandibular abductor muscle is visible.

### Palatine

The palatine shapes the most part of the interfenestral bridge between the suborbital fenestrae. Its surface is smooth, but bears many small foramina, especially in the anterolateral region (GPIT/RE/09761 Fig. 2). Anteriorly, the palatine is fan-shaped, but does not produce a shelf into the suborbital fenestra.

**Figure 6:**
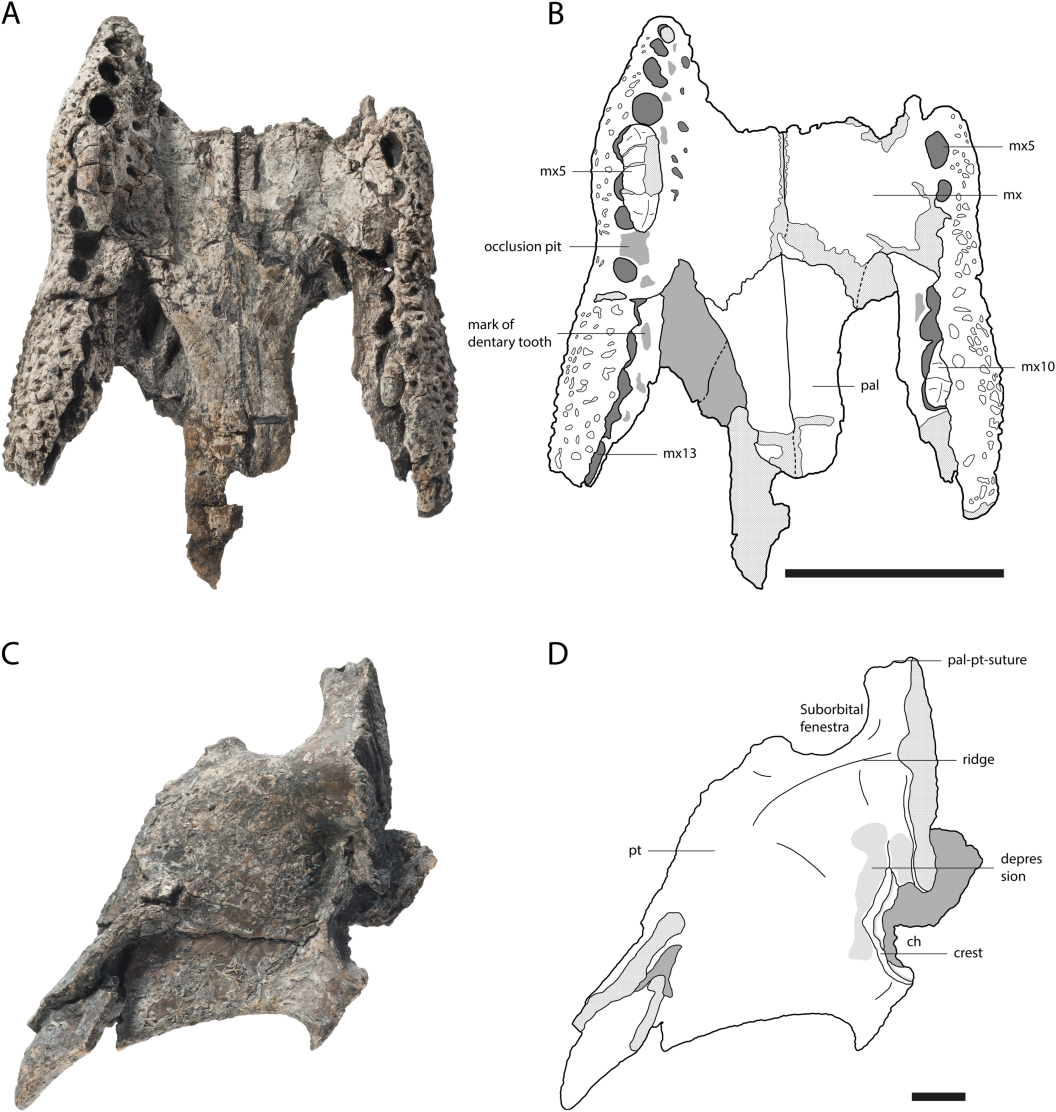
Partial palate (GPIT/RE/09769) and pterygoid (GPIT/RE/09728) of *Orientalosuchus naduongensi* Na Duong Formation, upper Eocene, Vietnam. Skull (A and B) and pterygoid (C and D) in ventral view. Abbreviations: ch, choana; mx, maxilla; mx5, maxilla tooth 5; mx10, maxilla tooth 10; mx13, maxilla tooth 13; pal, palatine; pt, pterygoid. Scale = 5 cm (A and B). Scale = 1 cm (C and D).

Anteriorly, the palatine does not reach at all, or protrudes only slightly beyond, the suborbital fenestra and contacts the maxilla with an obtuse V-shaped suture (GPIT/RE/09761 Fig. 2, GPIT/RE/09730 Fig. 3 and GPIT/RE/09769 Fig. 6). The suture with the pterygoid lies in front of the posterior end of the suborbital fenestra and is nearly straight lateromedially, except for a small midline process from the pterygoid projecting into the palatine (GPIT/RE/09761 Fig. 2).

The suborbital fenestrae are anteroposteriorly very large, reaching anteriorly the level of the inline occlusion between the seventh and eighth maxillary teeth (GPIT/RE/09730 Fig. 3). Their medial border is anteroposteriorly nearly straight, whereas the lateral border is slightly constricted due to the ectopterygoid reaching into the fenestra. The posterior border of the fenestra is smooth without a notch.

### Pterygoid

The pterygoid is preserved in GPIT/RE/09761 (Fig. 2) and GPIT/RE/09729 (Fig. 4). It forms the posterior and posteromedial borders of the suborbital fenestra and contacts the palatine anterior to the posterior end of the fenestra. Dorsally, the pterygoid extends further anteriorly along the suborbital bridge and reaches the level of the prefrontal pillar (Fig. 4). Posteriorly, it is lateromedially straight, except for a very prominent pair of the posterior pterygoid processes. Laterally, it contacts the ectopterygoid with a posterolateral projecting suture. Although the anterior part of the suture is not optimally preserved, a flexure seems to be absent.

The pterygoid surface is uneven. In the medial region, posterior to the suborbital fenestra there is a prominent bulge, which transforms into a posterolaterally and a posteromedially projecting ridge. The posterolateral ridge flattens shortly after, whereas the posteromedial ridge projects caudally until reaching the choana opening. The choana itself is not entirely preserved, but its rough outline is still visible. Its orientation cannot be determined due to dorsoventral crushing, although it seems to be anteroventrally oriented in a juvenile individual (GPIT/RE/09772). A septum seems present at least anteriorly as indicated in a CT-scan of GPIT/RE/09761, but it is unclear if it projects out of the choana or remains recessed within. In adult individuals, the posterolateral margin of the choana is smooth. In the juvenile GPIT/RE/09772, the posterolateral margin seems more concave (notched), but this could be a crushing artifact.

**Figure 7:**
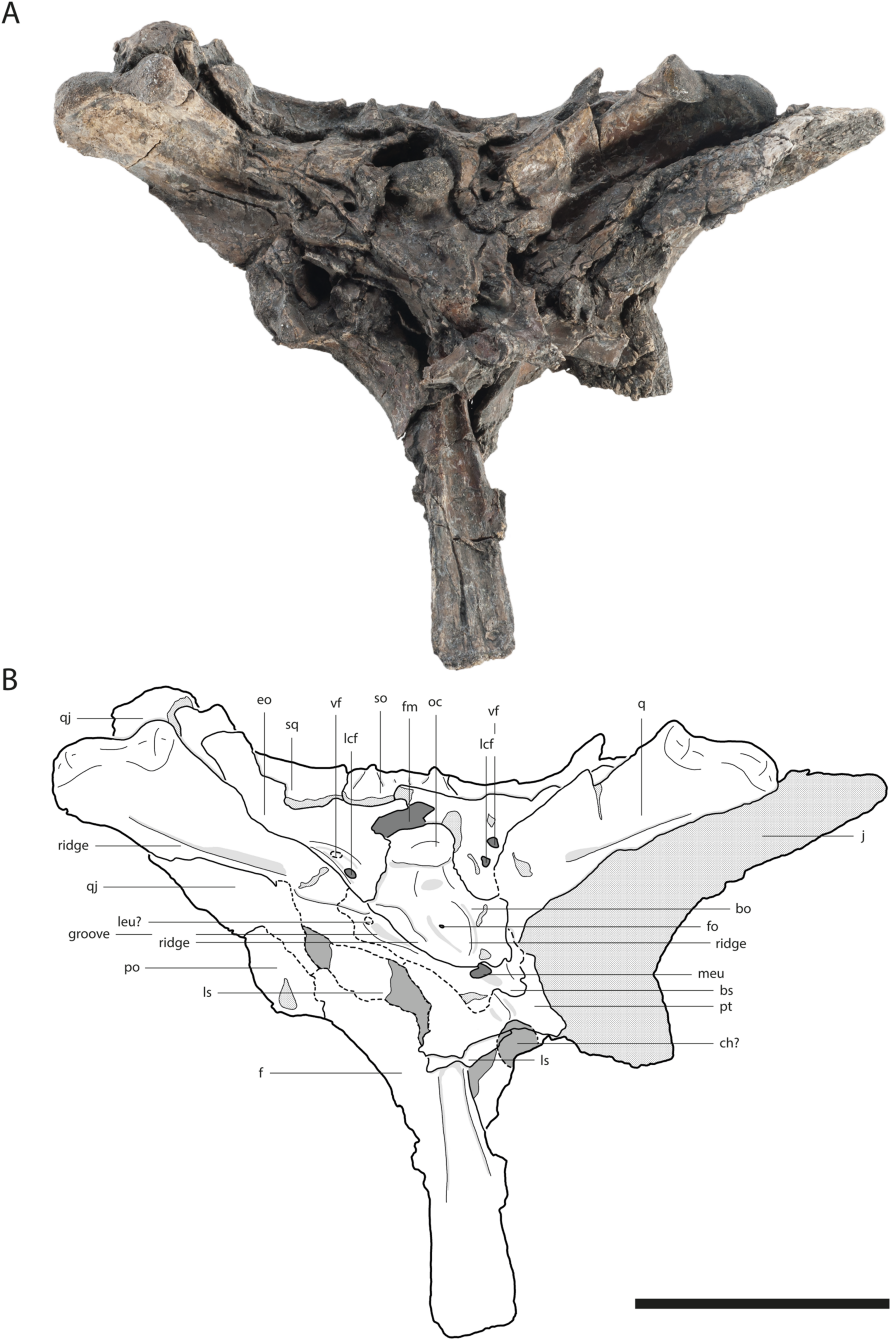
Skull of *Orientalosuchus naduongensis* (GPIT/RE/09729), Na Duong Formation, upper Eocene, Vietnam. Skull in occipital (A and B) view. Abbreviations: bo, basioccipital; bs, basisphenoid; ch, choana; eo, exoccipital; f, frontal; fm, foramen magnum; fo, foramen; j, jugal; lcf, lateral carotid foramen; leu, lateral eustachian opening; ls, laterosphenoid; meu, medial eustachian opening; oc, occipital condylus; po, postorbital; pt, pterygoid; q, quadrate; qj, quadratojugal; so, supraoccipital; sq, squamosum; vf, vagus foramen. Scale = 5 cm.

Anterolateral to the choana, the pterygoid surface is pushed inward and forms a thin neck surrounding the choana opening in GPIT/RE/09761 (Fig. 2) and GPIT/RE/09728 (Fig. 6). Between the posterior border of the choana and the suture with the basisphenoid, the pterygoid has a shallow medial ridge (GPIT/RE/09728 Fig. 7).

### Ectopterygoid

The ectopterygoid forms the posterolateral border of the suborbital fenestra. Anteromedially, the bone tapers into an acute tip and expands posteriorly, forming a small medial shelf projecting into the suborbital fenestra. Posteromedially to the suborbital fenestra, the ectopterygoid contacts the pterygoid and forms a posterolaterally projecting process that does not reach as far posteriorly as the pterygoid. The ectopterygoid does not abut the maxillary tooth row as seen in GPIT/RE/09730 (Fig. 3) and dorsally it terminates ventrally to the postorbital bar.

The surface of the anterior process bears many small foramina, whereas the posterior region is decorated with very fine anteroposteriorly oriented lines, visible in GPIT/RE/09761 (Fig. 2) and GPIT/RE/09729 (Fig. 4).

### Supraoccipital

The supraoccipital is the most dorsal bone of the occipital region and has an ornamented dorsal surface. It forms a large trapezoid process on the skull table, which prevents the parietal from reaching the posterior edge of the skull table in adults. In a juvenile (GPIT/RE/09772), the supraoccipital is still large, but the parietal has a minor lateral contact with the skull table.

GPIT/RE/09728 (Fig. 5) offers the best occipital view for the supraoccipital. The bone has a dominant, strictly dorsoventrally oriented ridge, which is most prominent dorsally and becomes shallow ventrally. Laterally, there is a pair of even more dominant ridges, which extends parallel to the medial one. Between these ridges, the bone is deeply pushed inwards. The posttemporal fenestra has oval outline and slopes ventrolaterally.

The suture with the exoccipital originates ventrally to the posttemporal fenestra and projects ventromedially resulting in a roughly triangular outline of the bone.

### Exoccipital

The exoccipital is best observable in GPIT/RE/09728 (Fig. 7). It shapes most of the occipital region and the paroccipital process. Further, it surrounds the foramen magnum nearly completely. Although the foramen magnum is crushed, its posterolateral margin can still be seen as well as the lateral pillars of the exoccipital, which were attached to the occipital condyle.

Alongside the ventrolaterally extending suture with the basioccipital, the exoccipital possesses a relatively short ventrally oriented process. Laterally from the occipital condyle, the caudal aperture of the carotid foramen is visible, and dorsolaterally from the occipital condyle lies the opening for the foramen vagus. The suture with the basisphenoid is not preserved.

### Basioccipital

The basioccipital forms the ventral part of the occipital region and the occipital condyle. Directly ventrally to the condyle, a small foramen is visible in GPIT/RE/09729 (Fig. 4), which is not present in GPIT/RE/09728 (Fig. 7). Further ventrally, the basioccipital forms a prominent dorsoventrally projecting medial ridge leading into the median eustachian opening. On the lateral side of the ridge, a small foramen is visible in GPIT/RE/097298 (Fig. 7). At the lateral contact with the basisphenoid, the basioccipital has a bulge, whereas the ventral suture around the median eustachian opening is relatively smooth.

### Basisphenoid

The basisphenoid is anteroventrally located from the basioccipital and projects relatively far ventrally. Its extension on the lateral braincase wall cannot exactly be determined in GPIT/RE/09728 (Fig. 7), but it looks relatively narrow in GPIT/RE/09761 (Fig. 2). The lateral eustachian opening is not preserved, but a canal potentially leading into it can be seen in GPIT/RE/09728 (Fig. 7). This reconstructs the opening at the same height as the dorsal end of the medial ridge of the basioccipital. The suture between the basisphenoid and pterygoid is strongly curved with the basisphenoid sending a rounded process ventrally into the pterygoid.

### Dentary

**Figure 8:**
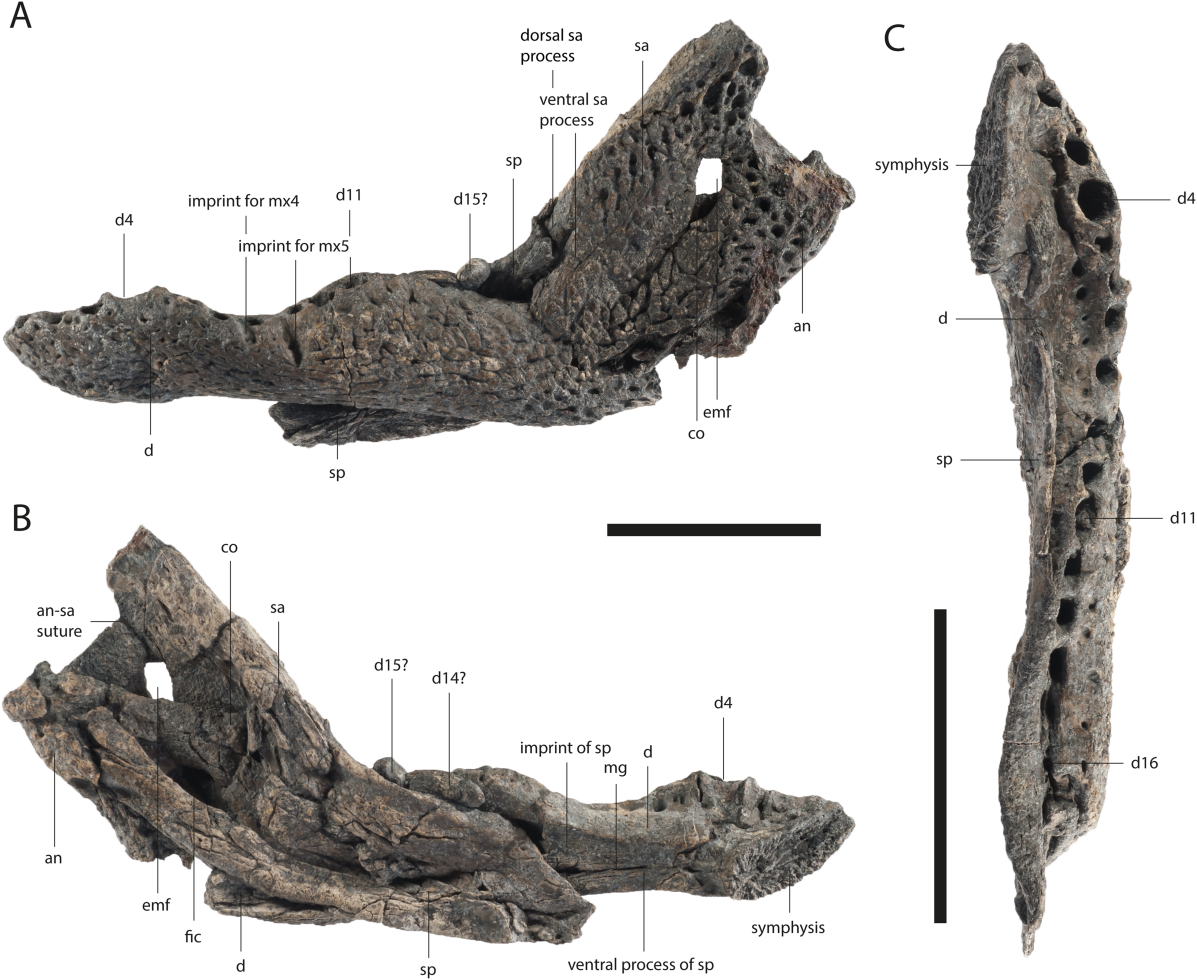
Lower jaw of *Orientalosuchus naduongensis* (GPIT/RE/09728), Na Duong Formation, upper Eocene, Vietnam. Lower jaw in lateral (A), medial (B) and dorsal (C) view. Abbreviations: an, angular; co, coronoid; d, dentary; d1–16, dentary tooth 1–16; emf, external mandibular fenestra; fic, foramen intermandibularis caudalis; mg, meckelian groove; mx4-5, maxilla tooth 4-5; sa, surangular; sp, splenial. Scale = 5 cm.

**Figure 9:**
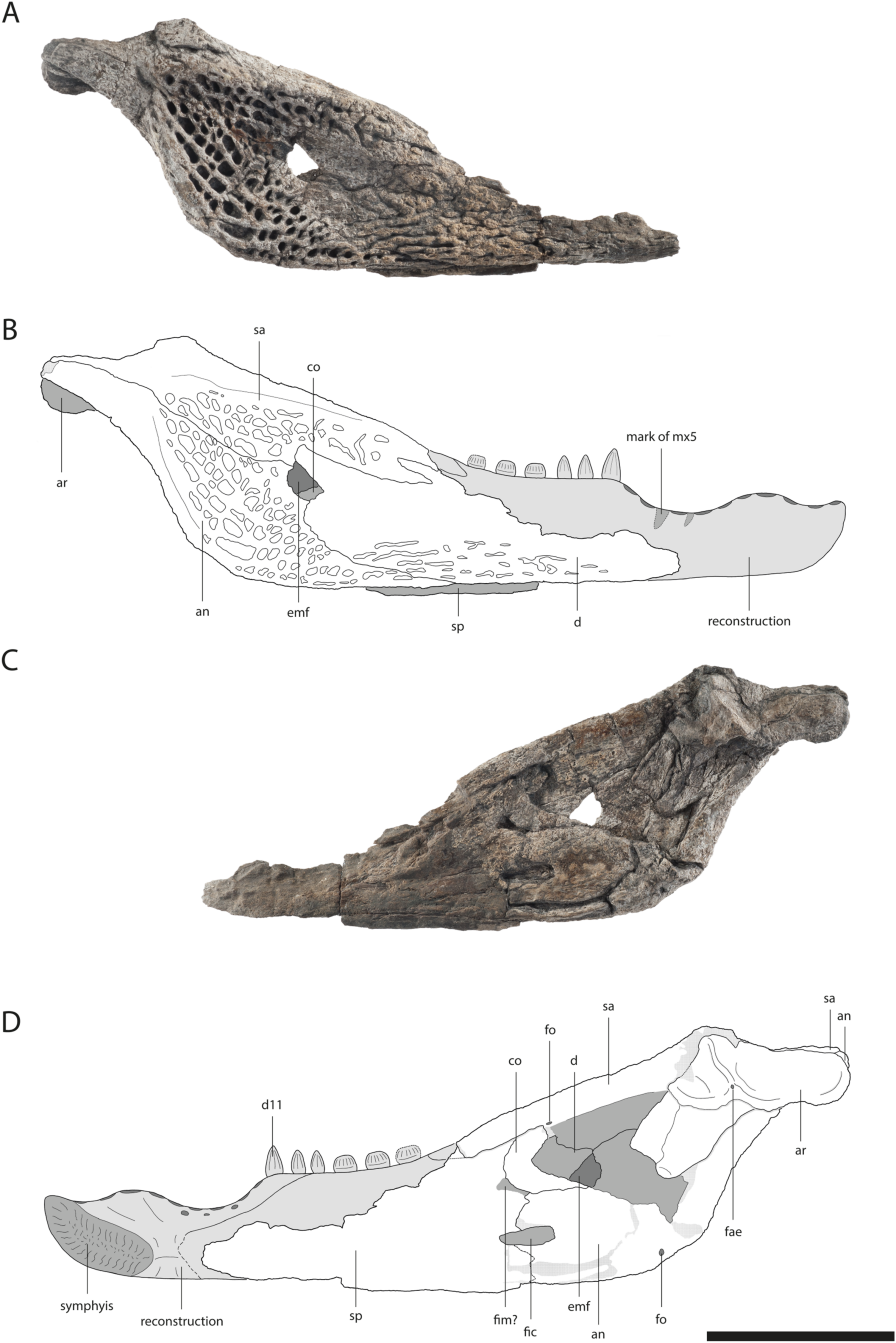
Lower jaw of *Orientalosuchus naduongensis* (GPIT/RE/09727), Na Duong Formation, upper Eocene, Vietnam. Lower jaw in lateral (A and B) and medial (C and D) view. Abbreviations: an, angular; ar, articular; co, coronoid; d, dentary; d11, dentary tooth 11; emf, external mandibular fenestra; fo, foramen; fic, foramen intermandibularis caudalis; fim, foramen intermandibularis medius; mx5, maxilla tooth 5; sa, surangular; sp, splenial. Scale = 5 cm.

The dentary is best observable in GPIT/RE/09728 (Fig. 8) and GPIT/RE/09727 (Fig. 9). It lies nearly completely lingually to the maxilla as observable in GPIT/RE/09761 (Fig. 2). The only exception is the region around the fourth and around the 11th dentary alveoli where an inline occlusion with the maxillary tooth row occurs (indicated by occlusion pits in GPIT/RE/09730 Fig. 3 and GPIT/RE/09769 Fig. 6). The general outline of the tooth row of the dentary is strongly sigmoidal. There is a shallow curvation between the first and fourth alveoli and a much deeper one between the fourth and 11th alveoli. The level of the 11th alveolus is slightly higher than the level of the fourth one. Below the posteriormost teeth, the outline is nearly even.

In total the tooth row consists of 16 teeth (Fig. 8). The first three alveoli are nearly equal in size, whereas the fourth one is much larger, fitting in the notch between the premaxilla and maxilla. The fifth and sixth alveoli are very small, with the fifth being the smallest of the dentary. The seventh to the 10th alveoli, are as large as the third. Between the seventh and eighth alveoli, there is a small diastema for receiving the fourth maxillary tooth. A larger diastema is present between the eighth and ninth alveoli for the massive fifth maxillary tooth, which left a very prominent mark on the dentary. The 11th alveolus is slightly larger than the previous ones and the second largest alveolus in the dentary. The 11th dentary tooth is most likely the one interfingering between the seventh and eighth maxillary teeth. The 12th and 13th alveoli are again smaller. The posterior alveoli of the 14th to 16th alveoli are lateromedially flattened and anteroposteriorly elongated.

The dentary surface is ornamented with small pits anteriorly and grooves posteriorly. Anteriorly to the external mandibular fenestra, nearly no ornamentation is visible. The symphysis extends to the height of the fifth dentary tooth. The Meckelian groove is preserved as a very narrow canal, nearly closed by the surrounding dentary.

The dentary-splenial suture abuts the tooth row at the level of the 13th dentary tooth. The dentary contacts the angular posteroventrally and the surangular posterodorsally. The suture with the angular projects ventrally from the height of the most posterior dentary tooth to the ventral part of the external mandibular fenestra in a bowed line. The suture with the surangular intersects the external mandibular fenestra at its posterodorsal corner. The external mandibular fenestra itself is very small. It has nearly the same size as the foramen intermandibularis caudalis (best seen in GPIT/RE/09727 Fig. 9). Despite its size, it forms posteroventrally a clear concavity with the angular. Its anterior margin, on the other hand, is straight.

### Splenial

The splenial lies lingually to the dentary and does not participate in the symphysis. Its surface is smooth. The splenial abuts the tooth row from the 13th tooth posteriorly and extends anteriorly to the level of the seventh dentary tooth. The anterior process passes ventrally to the Meckelian groove. This is visible in a single individual (GPIT/RE/09781) and in form of marks in GPIT/RE/09728 (Fig. 8). An anterior perforation for the mandibular ramus of the cranial nerve V is not present.

Posteriorly, the splenial meets the surangular, coronoid and angular, as well as it forms the anterior border of the foramen intermandibularis caudalis. The suture with the surangular projects anteriorly until reaching the tooth row. The concave suture with the coronoid is only visible in the right ramus of GPIT/RE/09727 (Fig. 9). The anteroventral part of the coronoid is damaged, but it seems the foramen intermandibularis medius was located completely on the splenial or at least the coronoid only borders the most posterior border of the foramen. The suture between the splenial and angular, ventrally to the foramen intermandibularis caudalis is ambiguous, but it seems to extend slightly posteriorly, before turning ventrally. Dorsally to the foramen, the suture is visible and projects nearly straight dorsoventrally without the splenial producing a posterior process between the angular and coronoid.

### Surangular

The surangular is best observable in GPIT/RE/09761 (Fig. 2) and GPIT/RE/09727 (Fig. 9) and shapes the posterodorsal portion of the lower jaw. The dorsolateral part of the bone is elevated laterally in form of a shallow bulge and densely ornamented with deep pits, whereas the anterior and the posterior parts around the retroarticular process does not possess ornamentation.

Anteriorly, the surangular has two processes. The ventral process is slightly shorter than the dorsal one, but they do not differ much in length. Posteriorly, the surangular extends to the posterior end of the retroarticular process. Dorsally, the surangular is slightly elevated, but does not seem to reach to the dorsal tip of the lateral wall of the glenoid fossa (best observable in GPIT/RE/09783). Laterally, the suture with the angular contacts the external mandibular fenestra slightly ventrally to its posterodorsal corner. Lingually, the suture between the surangular and articular cannot be clearly followed within the glenoid fossa, due to deformation. The suture ventrally to the fossa is simple, without any visible lamina. A lingual foramen is not preserved. The surangular-angular suture meets lingually the articular dorsally to the ventral tip of the articular. Posterodorsally to the surangular-coronoid suture, a small foramen is visible in GPIT/RE/09727 (Fig. 9).

### Angular

The angular is best observable in GPIT/RE/09761 (Fig. 2) and GPIT/RE/09727 (Fig. 9). Its lateral surface is densely ornamented with deep posteriorly elongated pits. The retroarticular process has no ornamentation. Laterally, the angular forms the concave posterior border of the external mandibular fenestra.

The bone borders most of the foramen intermandibularis caudalis dorsally and ventrally as well as its complete posterior border. The angular-coronoid suture is nearly straight and anteroposteriorly oriented. Ventrally, anteriorly to the height of the glenoid fossa, a relatively large foramen is visible. Another posterior foramen, is only preserved in GPIT/RE/09761 (Fig. 2).

### Coronoid

The coronoid is only well preserved in GPIT/RE/09727 (Fig. 9). Its outline is roughly boomerang-shaped with an incomplete posterodorsal part. The ventral process is relatively large and projects posteriorly. The bone has a smooth surface and does not bear any foramina.

### Articular

The articular possesses the glenoid fossa and the posterodorsally oriented retroarticular process in its medial part. Anteromedially, the process produces a broad shelf. The glenoid fossa is separated from the process by a mediolaterally oriented sigmoidal-shaped ridge with its lateral part being slightly more anteriorly oriented than the medial one. The foramen aerum is visible in GPIT/RE/09727 (Fig. 9) on the medial corner of the ridge.

### Teeth

**Figure 10:**
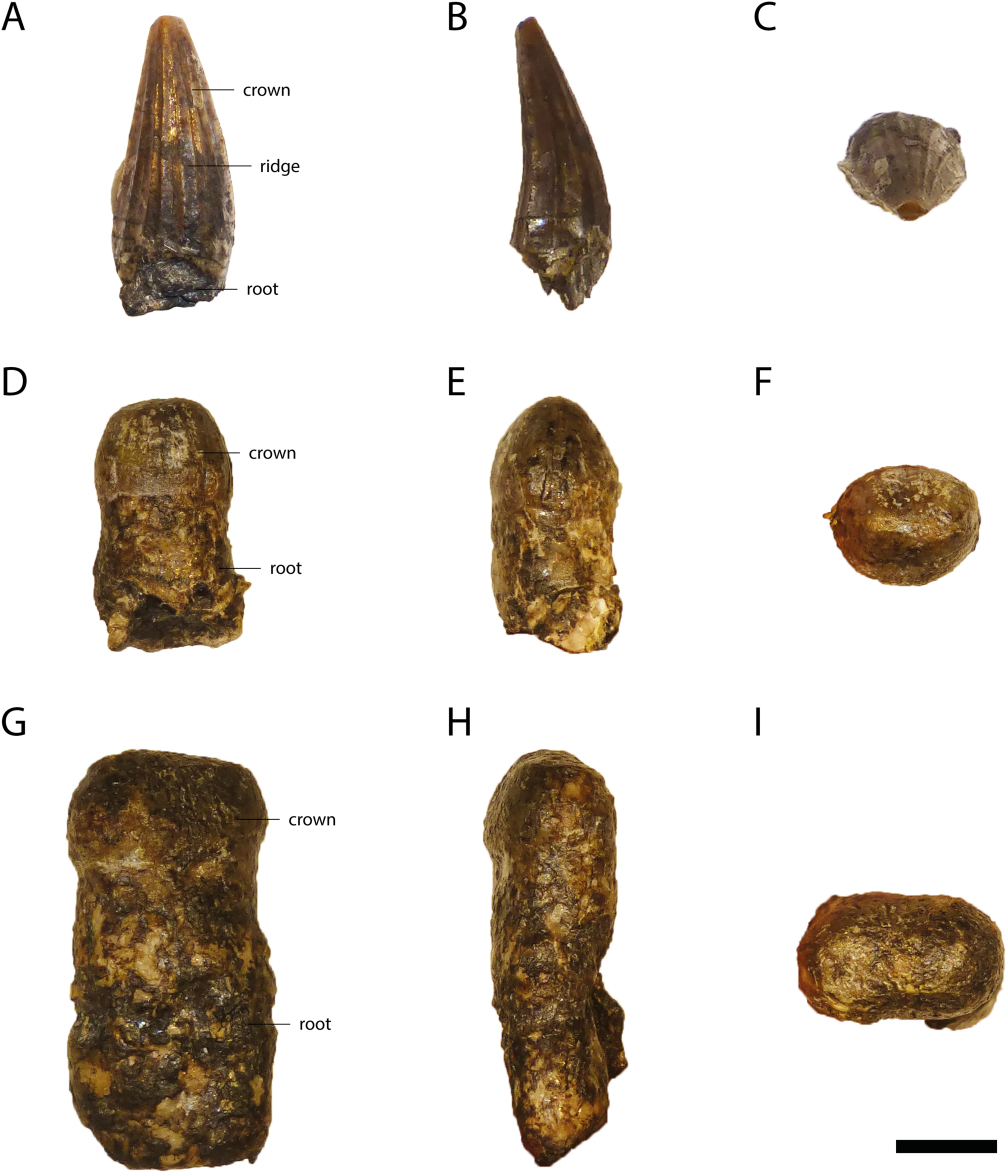
Tooth morphology of *Orientalosuchus naduongensis*, Na Duong Formation, upper Eocene, Vietnam. Anterior tooth of GPIT/RE/09761 (holotype) in lateral (A), anterior (B) and dorso/ventral (C) view. Medial tooth of GPIT/RE/09728 in lateral (D), anterior (E) and dorso/ventral (F) view. Posterior tooth of GPIT/RE/09728 in lateral (G), anterior (H) and dorso/ventral (I) view. Scale = 0.5 cm.

Most of the teeth attached to the skulls and lower jaws are poorly weathered. Only disarticulated teeth of GPIT/RE/09728 (posterior ones) and GPIT/RE/09761 (a single anterior one) (Fig. 10) are well preserved, but their original positions are not clear. Based on the size of the alveoli, the fourth and 11th dentary teeth were the largest teeth in the lower jaw, whereas in the upper jaw, the fifth maxillary tooth was by far the largest one and left deep marks on the lateral dentary wall (GPIT/RE/09728 Fig. 8).

The anterior and middle teeth are pointed. In a single well-preserved tooth several relatively dominant vertical ridges are present laterally and weaker ones lingually. This condition is not visible in poorly-preserved teeth, making it unclear if this condition is true for all pointed teeth. Laterally, the teeth are slightly convex, whereas they are concave lingually.

Posteriorly, at the 10th maxilla tooth (GPIT/RE/09769 Fig. 6) the teeth become conical and blunt.

The most posterior three teeth are relatively large, have a very blunt crown and are anteroposteriorly elongated and laterally compressed. They are smaller than in typically bulbous tooth taxa like *Hassiacosuchus haupti* Weitzel, 1935.

Both types of posterior teeth bear fine dorsoventrally oriented lines, but no clear vertical ridges are present like in the well-preserved anterior one (Fig. 10).

### Postcranial Description

Most of the postcranial material is preserved in GPIT/RE/09761, GPIT/RE/09727 and GPIT/RE/09784. If not otherwise stated the description is based on the holotype GPIT/RE/09761. Measurements are deposited in the File S4.

### Atlas

From the atlas, only the rectangular intercentrum is preserved dislocated into the left orbit of GPIT/RE/09761 (Fig. 2). The bone is plate-shaped in lateral view and has prominent parapophyseal processes. The slightly convex ventral part has a shallow anteroposteriorly projecting central groove.

### Axis

**Figure 11:**
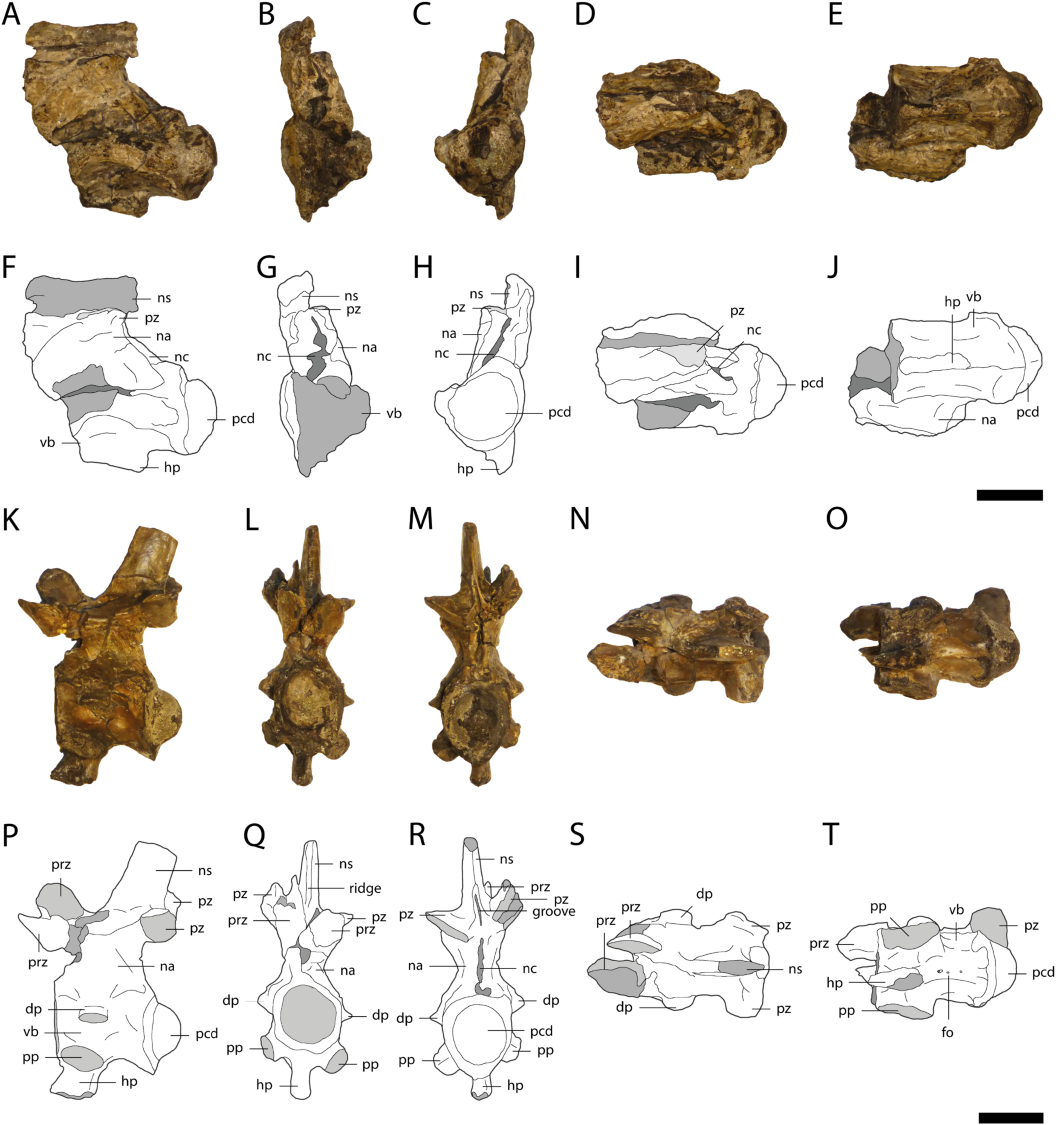
Cervical vertebrae of *Orientalosuchus naduongensis* (GPIT/RE/09761) (holotype), Na Duong Formation, upper Eocene, Vietnam. Cervical vertebrae in lateral left (A and F), anterior (B and G), posterior (C and H) dorsal (D and I), and ventral (E and J) view. Cervical vertebra in lateral left (K and P), anterior (L and Q), posterior (M and R) dorsal (N and S), and ventral (O and T) view. Abbreviations: dp, diapophysis; hp, hypapophysis; na, neural arch; nc, neural canal; ns, neural spine; pcd, posterior condylus; pp, parapophysis; prz, prezygapophysis; pz, postzygapophysis; vb, vertebra body. Scale = 1 cm.

The axis (Fig. 11) is better preserved posteriorly. The neural spine looks completely horizontal, but its surface is weathered. The base of the postzygapophysis is visible on the left side, but also poorly preserved. The neural arch and neural canal are laterally compressed due to fossilization. The hypapophysis is posteriorly shifted.

### Cervical vertebrae

A total of 18 cervical vertebrae are preserved (seven in GPIT/RE/09761 and GPIT/RE/09784, three in GPIT/RE/09727 and one in GPIT/RE/09767 (1). All of these are crushed and/or covered by a pyritized matrix except for the single vertebra of GPIT/RE/09761 (Fig. 11).

The neural spine is slightly sloping anteriorly, but the most dorsal tip is not preserved. The spine has a dorsoventrally extending anterior ridge and a posterior groove, with the latter deepest between the postzygapophyses. The hypapophysis is located at the anterior part of the centrum and reaches posteriorly roughly its midpoint. The pre- and postzygapophyses are similarly formed and have oval articular surfaces. The diapophysis initiates above the base of the neural arch, whereas the parapophysis originates ventrally on the centrum. The centrum is concave medially, smooth and without any pits. A lateral foramen is preserved on the right side, slightly posteriorly between the diapophysis and parapophysis. A few smaller foramina are visible posterior to the hypapophysis.

### Dorsal vertebrae

**Figure 12:**
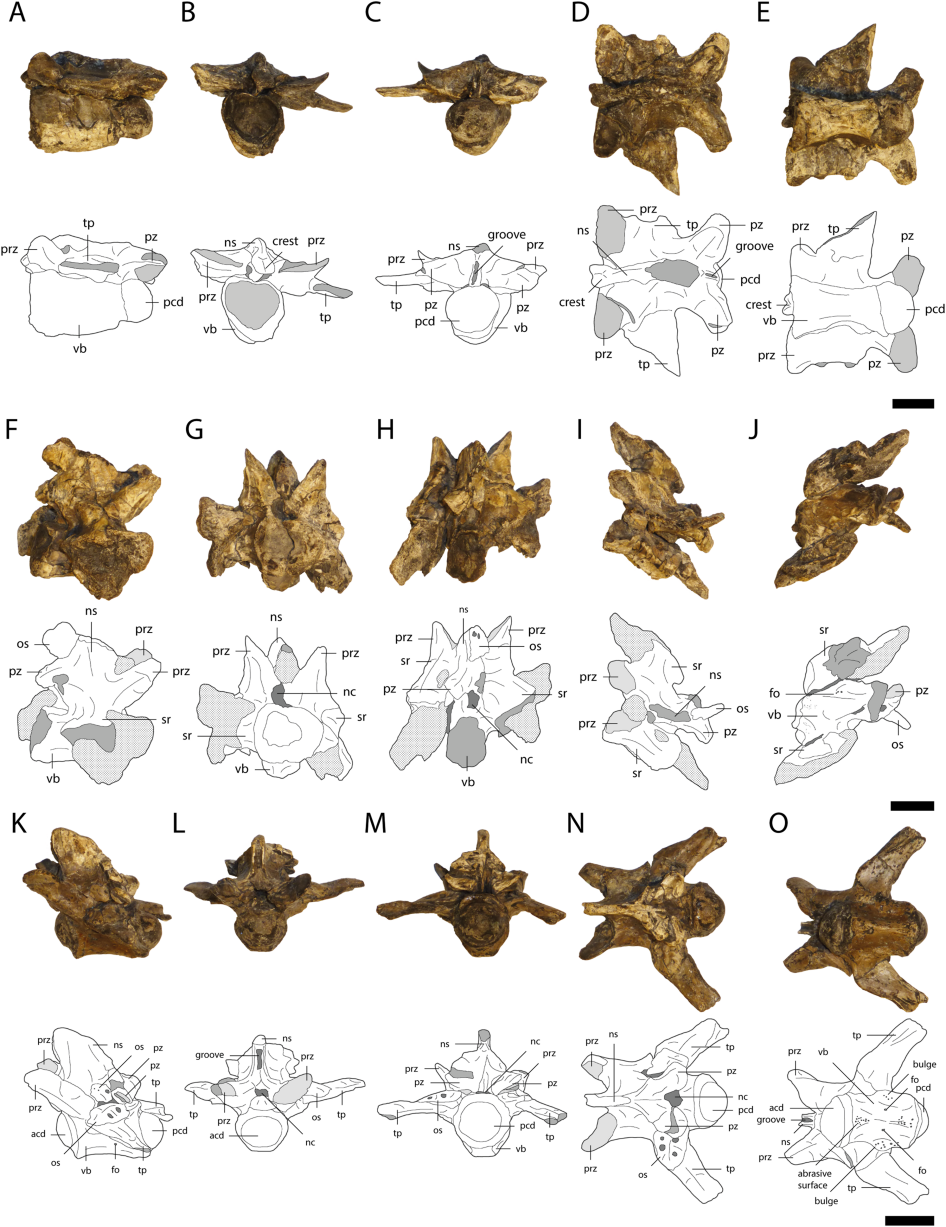
Vertebrae of *Orientalosuchus naduongensis* (GPIT/RE/09761) (holotype), Na Duong Formation, upper Eocene, Vietnam. Dorsal vertebra in lateral left (A), anterior (B), posterior (C), dorsal (D), and ventral (E) view. First sacral vertebra in lateral right (F), anterior (G), posterior (H), dorsal (I), and ventral (J) view. First caudal vertebra in lateral left (K), anterior (L), posterior (M), dorsal (N), and ventral (O) view. Abbreviations: acd, anterior condylus; fo, foramen; nc, neural canal; ns, neural spine; os, osteoderm; pcd, posterior condylus; prz, prezygapophysis; pz, postzygapophysis; sr, sacral rib; tp, transverse process; vb, vertebra body. Scale = 1 cm.

A total of 30 dorsal vertebrae are preserved (10 in GPIT/RE/09761, 11 in GPIT/RE/09727, one in GPIT/RE/09778 and eight in GPIT/RE/09784). They are best preserved in GPIT/RE/09761 (Fig. 12). The posterior dorsal vertebrae are anteroposteriorly elongated and lack a hypophyseal keel. The transverse processes are only preserved as small fragments. The neural spine has an anterior, dorsoventrally oriented keel and a posterior groove. In a single vertebra of GPIT/RE/09761, there is an anterior pit ventrally to the keel. The anterodorsal end of the neural spine possesses a large crest with a rounded outline, which forms a small horizontal plateau. The articular surfaces of the prezygapophyses are oval and slightly medially shifted. The articulation surfaces of the postzygapophyses are also oval, but facing straight ventrally. The vertebral centra are procoelous and differ in length. They are slightly concave and smooth without any visible foramina at the middle part of the lateral surface.

### Sacral vertebra

Only the first sacral vertebra (Fig. 12) is preserved, but it is twisted and deformed. The neural spine slopes slightly anteriorly. Its dorsal tip is missing. The prezygapophyses are relatively large, whereas the only preserved left postzygapophysis in comparison is very small. The articulation facets of the prezygapophyses are oriented medially, whereas the postzygapophysis is ventrolaterally oriented. The centrum has a smooth surface with a small foramina ventrally.

### Caudal vertebrae

A total of 19 caudal vertebrae are preserved (eight in GPIT/RE/09761, six in GPIT/RE/09727 and five in GPIT/RE/09784). The first caudal vertebra in GPIT/RE/09761 (Fig. 12) is by far the best preserved and can be distinguished from the others by its convex anterior and posterior condyles. The neural spine of the first caudal vertebra originates relatively anteriorly and slopes posteriorly. Its anterior part is nearly vertical and has a large dorsoventrally extending groove. The prezygapophyses are relatively large and oval. Their articulation surfaces point dorsomedially. The transverse processes are laterally oriented at their bases, but bent posterolaterally. The centrum is slightly concave medially. The condyle is slightly larger than the cotyle. The lateral surface of the centrum is smooth with a lateroventrally located foramen on each side. In contrast, the antero- and posteroventral regions of the centrum have a rough surface with many small pores. A further rough surface is located posterodorsally on a small bulge of around 5 mm in length and continues from the base of the transverse process posteroventrally toward the condyle.

The rest of the caudal vertebrae are poorly preserved and their neural spines are narrower and originate more on the posterior part of the centrum. Their pre- and postzygapophyses are of similar size and relatively small. The prezygapophyses are very close to each other. Their articulation surfaces point medially. The transverse processes are fragmentarily preserved in the anterior caudal vertebrae and absent in the more posterior ones. The centra gradually elongate and flatten laterally as well as reduce in size posteriorly in the vertebral column. The centrum further has a broad, anteroposteriorly extending groove, which makes the centra even narrower and the articulation facets more oval posteriorly. The ventral side of the centra bears a deep anteroposteriorly projecting sulcus in all caudal vertebrae except for the first one.

### Ribs

**Figure 13:**
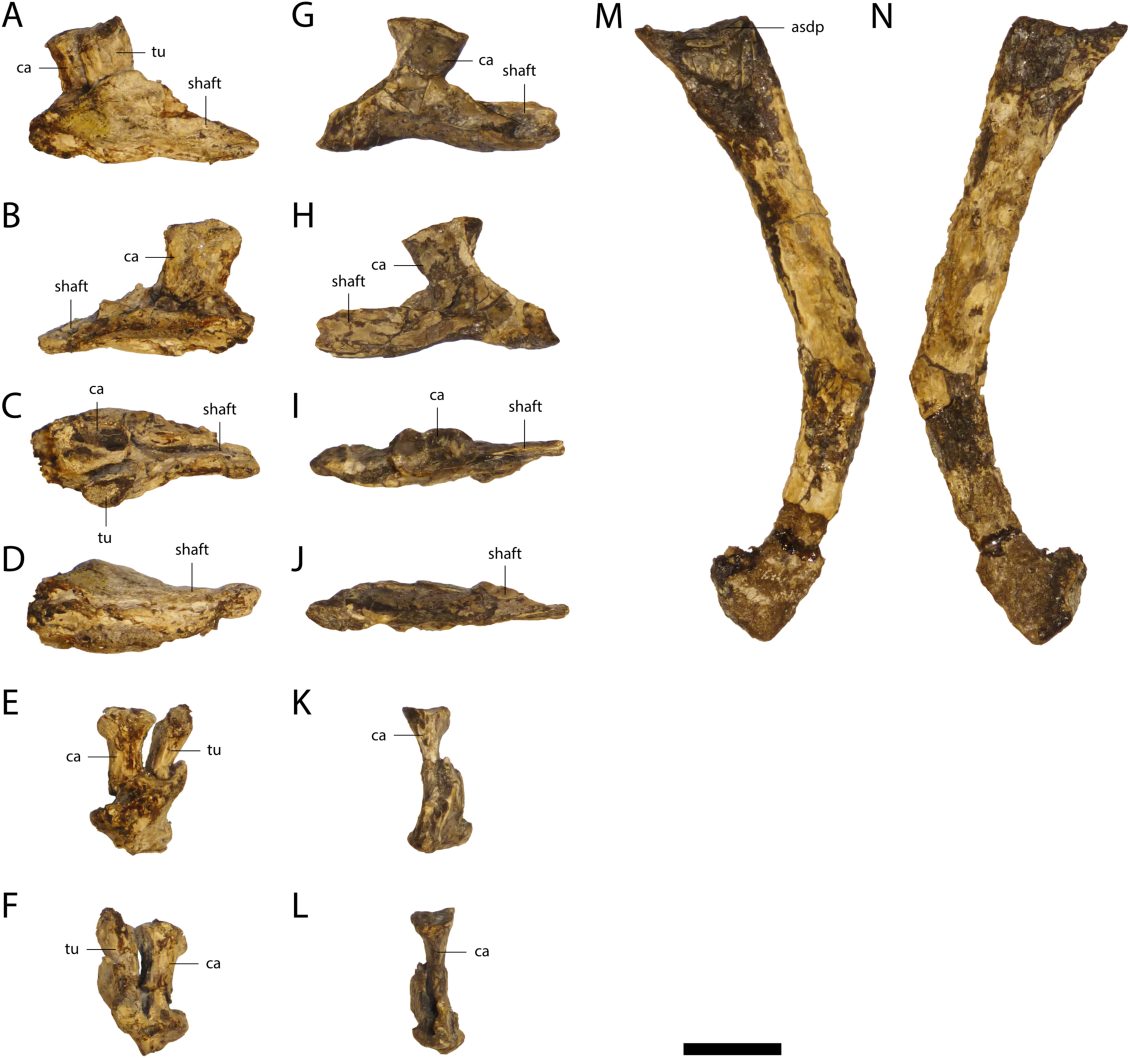
Ribs of *Orientalosuchus naduongensis* (GPIT/RE/09761) (holotype), Na Duong Formation, upper Eocene, Vietnam. Cervical ribs in lateral (A and G), medial (B and H), dorsal (C and I), ventral (D and J), anterior (E and K) and posterior (F and L) views; dorsal rib in lateral (M) and medial (N) view. Abbreviations: asdp, articulation surface with diapophysis; ca, capitulum; tu, tuberculum. Scale = 1 cm.

Three cervical and 10 dorsal ribs are preserved. The cervical ribs (Fig. 13) have a horizontally oriented shaft. The capitulum and tuberculum project at nearly 90° from this shaft near its anterior end. The capitulum is larger than the tuberculum and the articular surface of the former is nearly twice as large as the articulation surface of the latter.

The shaft of the dorsal ribs (Fig. 13) is slightly convex anteriorly and concave posteriorly. Its ventral end is broad anteroposteriorly, but flattened lateromedially.

### Scapula

**Figure 14:**
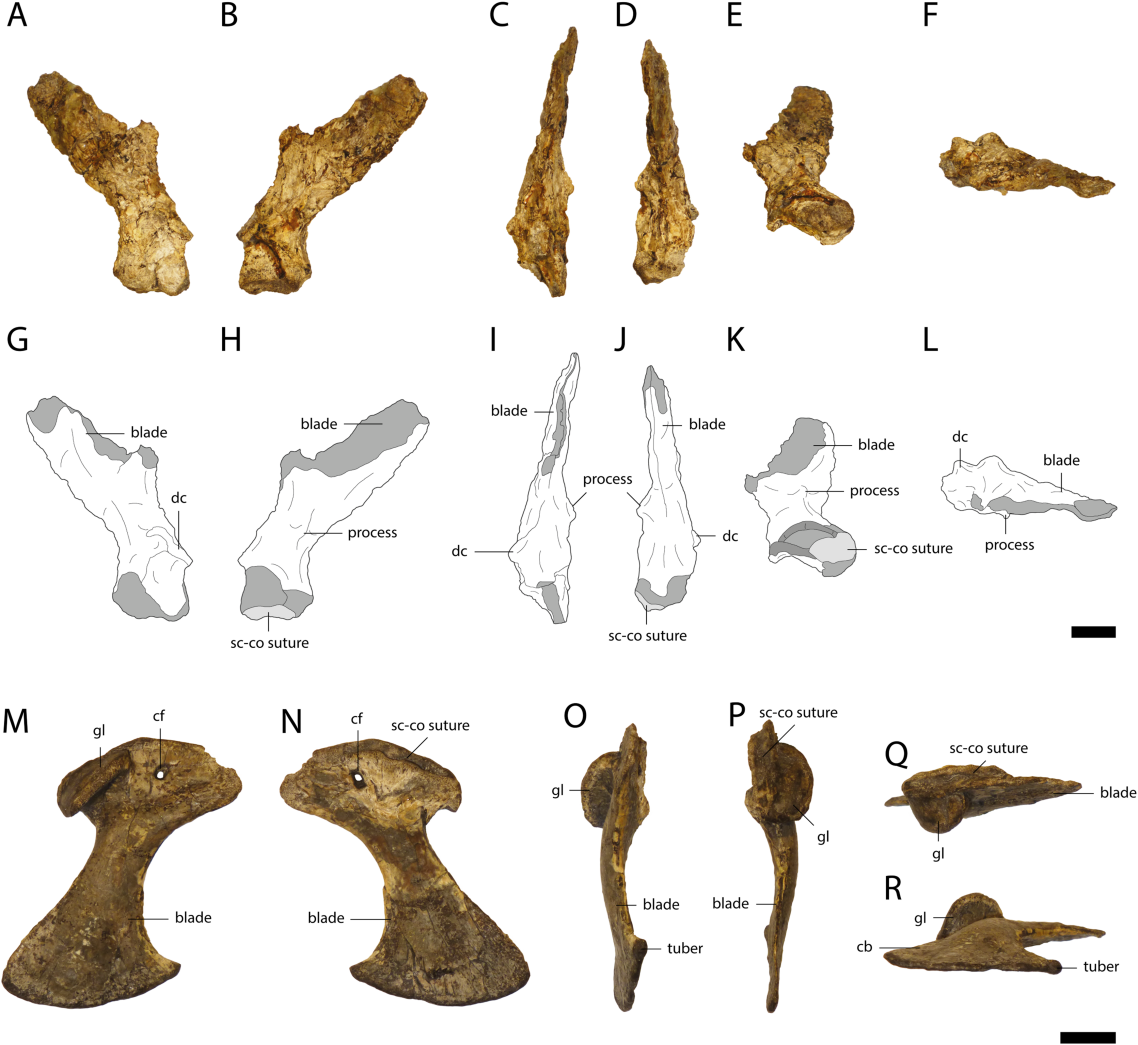
Pectoral girdle of *Orientalosuchus naduongensis* (GPIT/RE/09761) (holotype), Na Duong Formation, upper Eocene, Vietnam. Right scapula in lateral (A and G), medial (B and H), anterior (C and I), posterior (D and J), ventral (E and K) and dorsal (F and L) view. Right coracoid in lateral (M), medial (N), anterior (O), posterior (P), dorsal (Q), and ventral (R) view. Abbreviations: cf, coracoid foramen; dc, deltoid crest; gl, glenoid; sc-co suture, scapula-coracoid suture. Scale = 1 cm.

Two highly weathered right scapulae (GPIT/RE/09761, Fig. 14 and GPIT/RE/09784) and a well preserved, but broken left scapula (GPIT/RE/09727) were recovered. The scapular blade flares dorsally. The deltoid crest is damaged and located at the antero-ventral part of the constricted area between the base of the scapula and its blade. The crest seems to be narrow, but due to the weathering, this cannot be stated with confidence.

### Coracoid

The right coracoid of GPIT/RE/09761 (Fig. 14) is well preserved. Only the posteromedial part of the scapula-coracoid suture is weathered and pushed toward the glenoid, resulting in a deep postmortem notch along the suture. The glenoid is very broad, oval and anteriorly elongated. The coracoid foramen is located anteriorly to the glenoid near the scapula-coracoid suture. The coracoid blade is relatively broad and flares anteroposteriorly. It slopes slightly anteriorly at the connection surface with the interclavicula and ends with an anterior tip in form of a small tuber.

### Humerus

**Figure 15:**
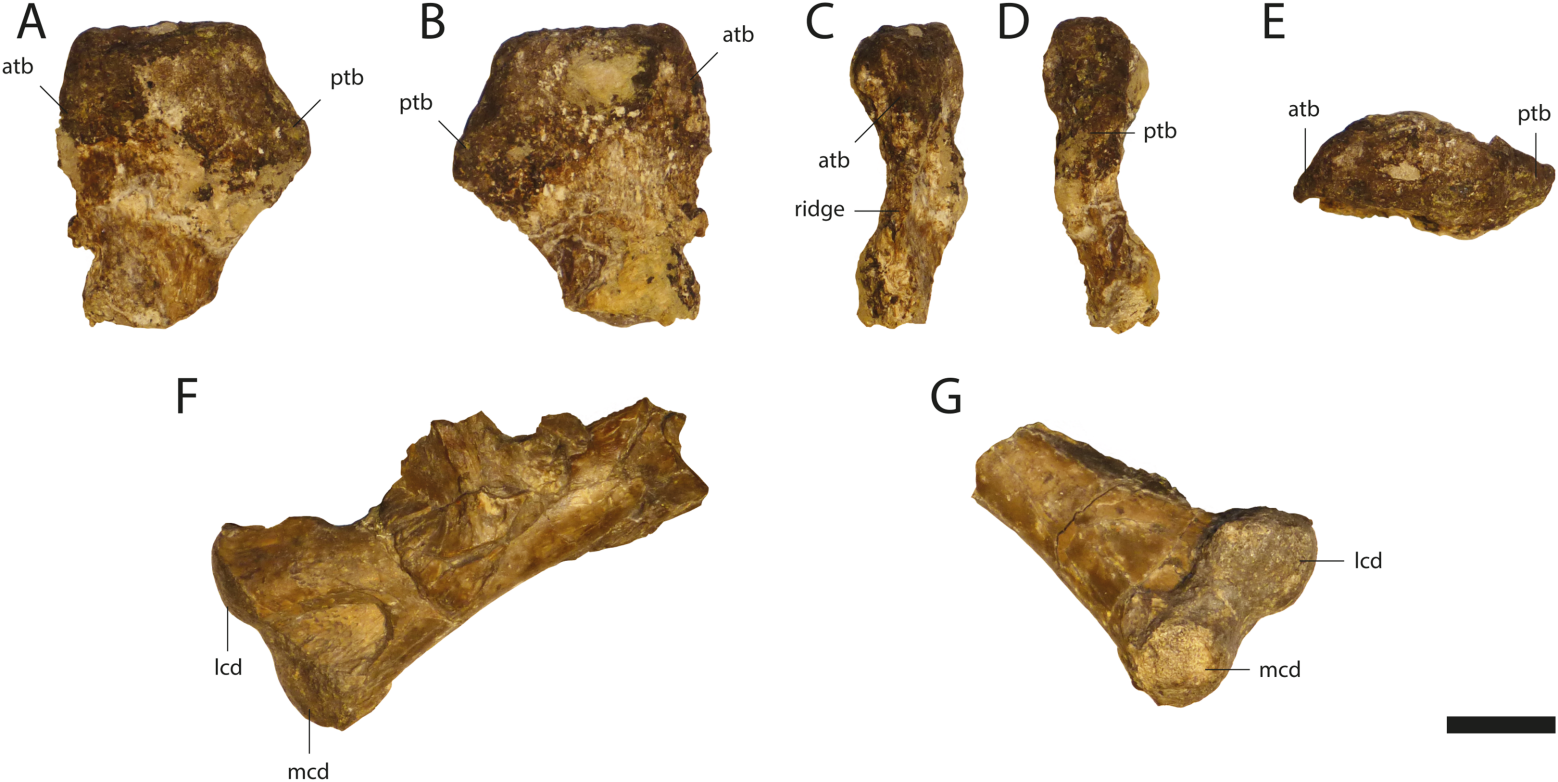
Humeri of *Orientalosuchus naduongensis* (GPIT/RE/09761) (holotype), Na Duong Formation, upper Eocene, Vietnam. Proximal portion of right humerus in dorsal (A), ventral (B), lateral (C), medial (D) and proximal (E) view. Distal portion of left humerus in dorsal (F) and ventro-distal (G) view. Abbreviations: atb, anterior tuberosity; lcd, lateral condylus; mcd, medial condylus; ptb, posterior tuberosity. Scale = 1 cm.

The humeri of GPIT/RE/09761 (Fig. 15) are partially preserved. The right humerus of GPIT/RE/09727 is complete in length (83.2 mm), but highly weathered. Its anterior part including the deltopectoral crest is missing.

The humeral head is only slightly elevated from the anterior tuberosity. The head and the anterior tuberosity form a nearly horizontal plateau, which bends slightly towards the latter. The posterior tuberosity lies on a small process distally from the humerus head. The deltopectoral crest is damaged, but the ridge between the crest and the anterior tuberosity is partially preserved.

The lateral condyle from the distal end is larger and nearly round, whereas the medial condyle is more oval. The shaft is slightly bowed posteriorly.

### Ulna/Radius

Only the right radius (63.1 mm) is preserved in GPIT/RE/09727. It has a very broad proximal and a broad distal end. The shaft is slightly S-shaped, which could be an artifact of crushing. The right and left ulnae of GPIT/RE/09784 are poorly preserved.

### Radiale

**Figure 16:**
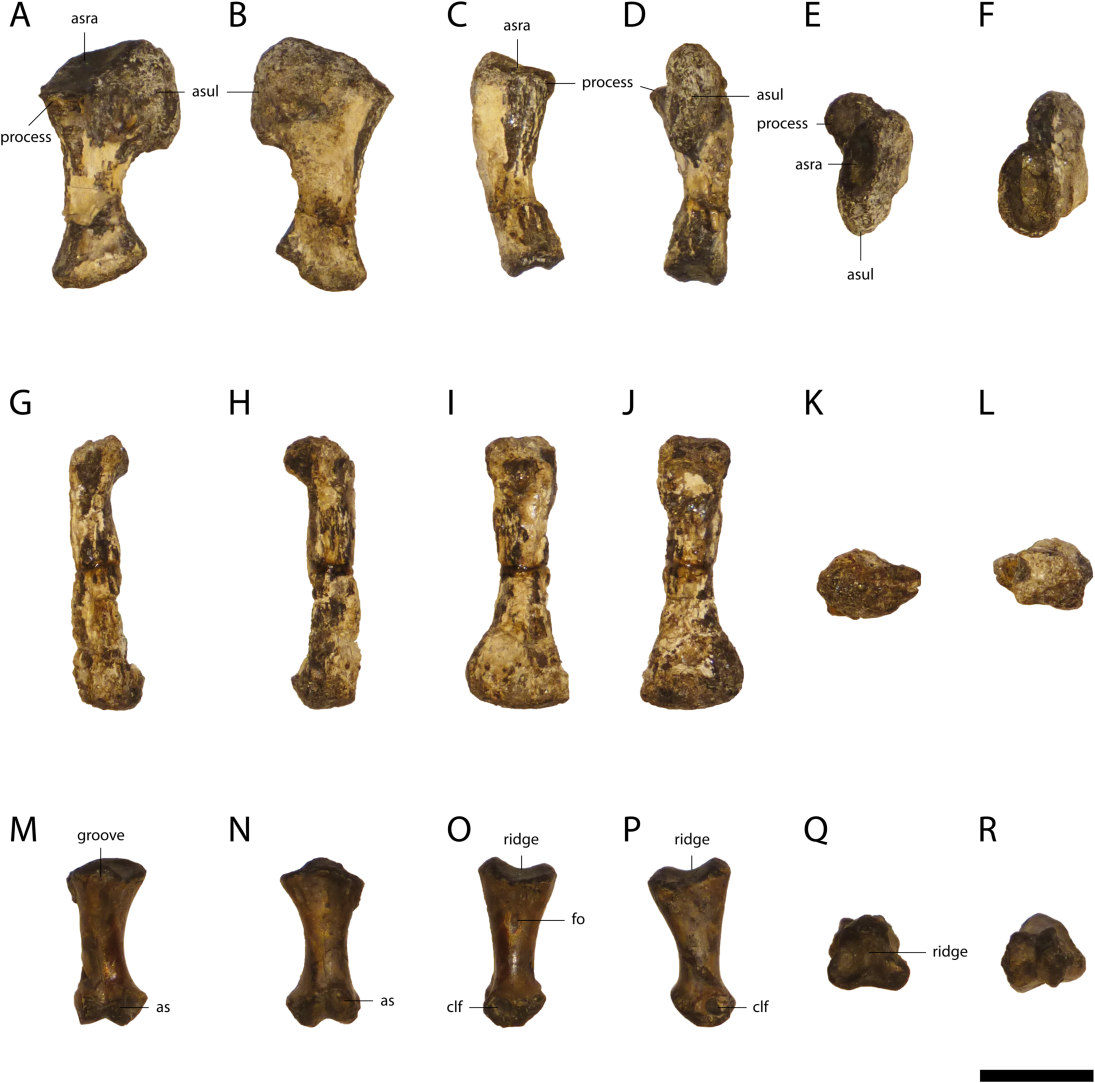
Left Radiale, Metapodial and Phalange of *Orientalosuchus naduongensis* (GPIT/RE/09761) (holotype), Na Duong Formation, upper Eocene, Vietnam. Left radiale in lateral (A), medial (B), anterior (C), posterior (D), proximal (E), and distal (F) view. Metapodial in lateral left or right (G and H), dorsal (I), ventral (J), proximal (K) and distal (L) view. Phalange in dorsal (M), ventral (N), lateral left or right (O and P), proximal (Q) and distal (R) view. Abbreviations: asra, articulation surface with radius; asul, articulation surface with ulna; clf, collateral ligament fossa; fo, foramen. Scale = 1 cm.

The left radiale (Fig. 16) is relatively well preserved, but slightly twisted and the medial part of the proximal end is eroded. The proximal end consists of the articular surface for the ulna and radius. Posteriorly the contact zone with the ulna is nearly vertical. The contact zone is very broad, giving the bone a P-shaped outline. In contrast, the contact area with the radius is kidney-shaped, nearly horizontal and slopes only slightly anterolaterally with a small lateral process. The shaft of the radiale is constricted, very thin and slightly sloping anteriorly. The distal end is oval and enlarged.

### Metapodials

In GPIT/RE/09761, elongated autopodial elements (Fig. 16), either metacarpals or metatarsals are preserved, but cannot be assigned with confidence. The autopodials of GPIT/RE/09784 represent metatarsals based on their position on the excavation side and are much longer than the elements in GPIT/RE/09761 suggesting that those are metacarpals. The distal portion of the shaft of the metapodials are dorsoventrally flattened. Their proximal end is oval, whereas their distal end with two condyles is smaller and narrower.

### Phalanges

There are seven disarticulated phalanges of GPIT/RE/09761 (Fig. 16), but we cannot determine whether they belong to the pes or manus. They are mostly highly weathered, only a single one is well preserved. The 14 phalanges of GPIT/RE/09784 belong to the pes based on their recovered position.

Its proximal end is nearly triangular with three knob-like structures, which are separated by a ridge. Its distal end has two condyles. Their articulation surfaces slope dorsally and ventrally. The condyles are separated from each other by an intercondylar groove. In lateral view, relatively deep collateral ligament fossae are visible on the distal condyles. The shaft has a smooth surface, but bears a foramen on the slightly laterally bent side.

### Ungual phalanges

The preserved claws of GPIT/RE/09761 differ considerably in length, but cannot be assigned to either manus or pes. They are long, only slightly curved, ventrally flattened, dorsally curved and pointed. The claws of GPIT/RE/09784 belong to the pes based on their position recorded in the field and are partly articulated with the distal phalanges. The better preserved ones are much smaller and stronger curved than the claws of GPIT/RE/09761, but it is unclear to which digit they belong.

### Ilium

**Figure 17:**
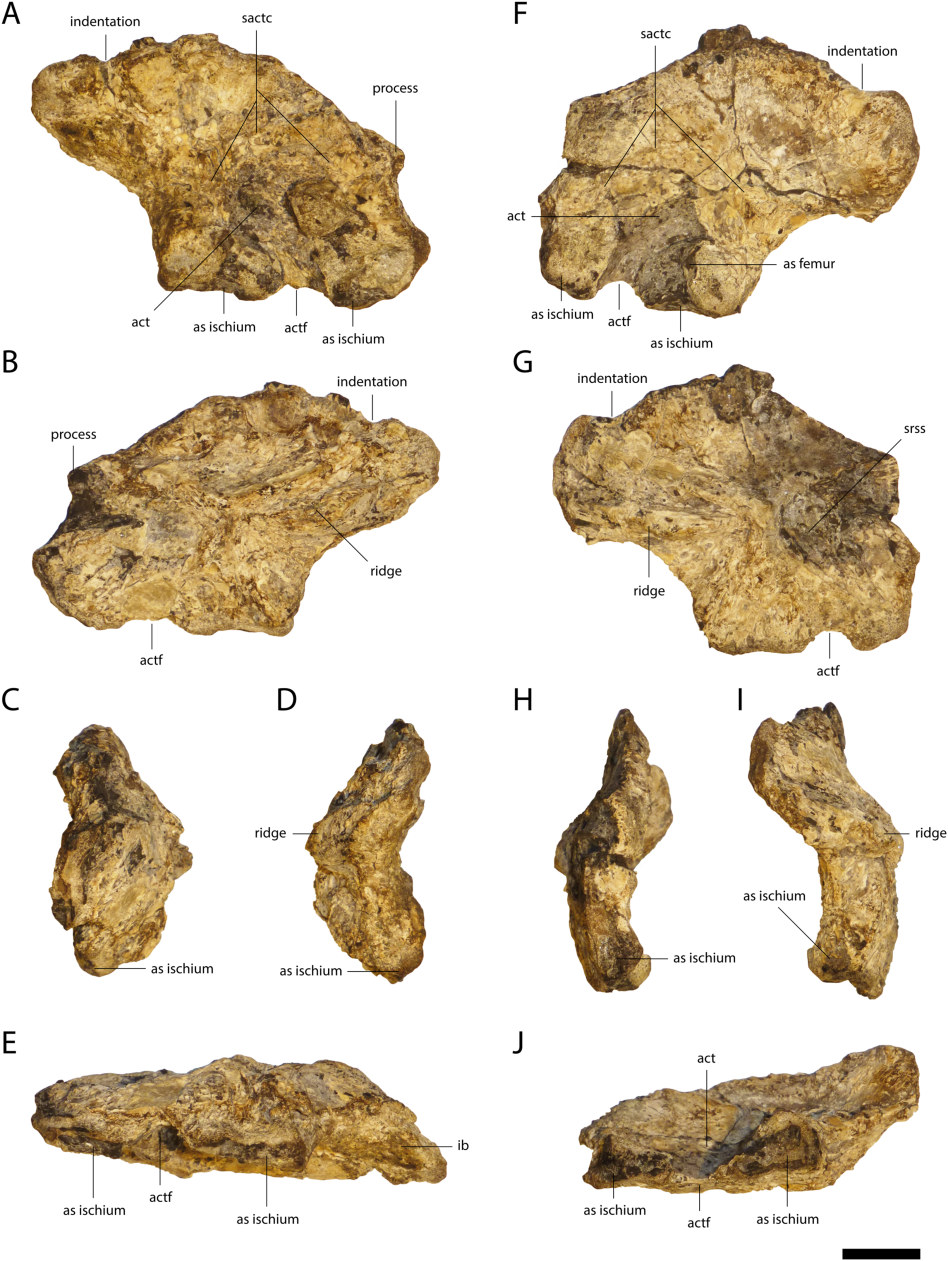
Ilia of *Orientalosuchus naduongensis* (GPIT/RE/09761) (holotype), Na Duong Formation, upper Eocene, Vietnam. Right ilium (left) and left ilium (right) in lateral (A and F), medial (B and G), anterior (C and H), posterior (D and I) and ventral (E and J) view. Abbreviations: as, articulation surface; act, acetabulum; actf, acetabulum foramen; sactc, supraacetabularcrest; srss, sutural surface for sacral rib. Scale = 1 cm.

Both ilia are preserved in GPIT/RE/09761 (Fig. 17) and GPIT/RE/09784. The posterior part of the iliac blade is rectangular and has a modest indentation dorsally. Anteriorly, the blade slopes at approximately 30° toward a small anterior process. The sutural surface for the first sacral rib is slightly visible on the anteromedial part in the left ilium. In ventral view, the posterior sutural surface for the ischium is triangular with a larger posterior part and a narrower anterior one. The anterior sutural surface is more oval and smaller. Dorsolaterally, an articulation surface with the femur is visible. The acetabulas foramen lies between two articular surfaces. The acetabulum itself seems relatively narrow. Dorsally from the acetabulum, the supraacetabular crest is visible. It separates the acetabulum from the iliac blade. Medially, at the articular surface with the second sacral rib, a prominent ridge is present, although the surface itself is not clearly visible due to weathering.

### Ischium

**Figure 18:**
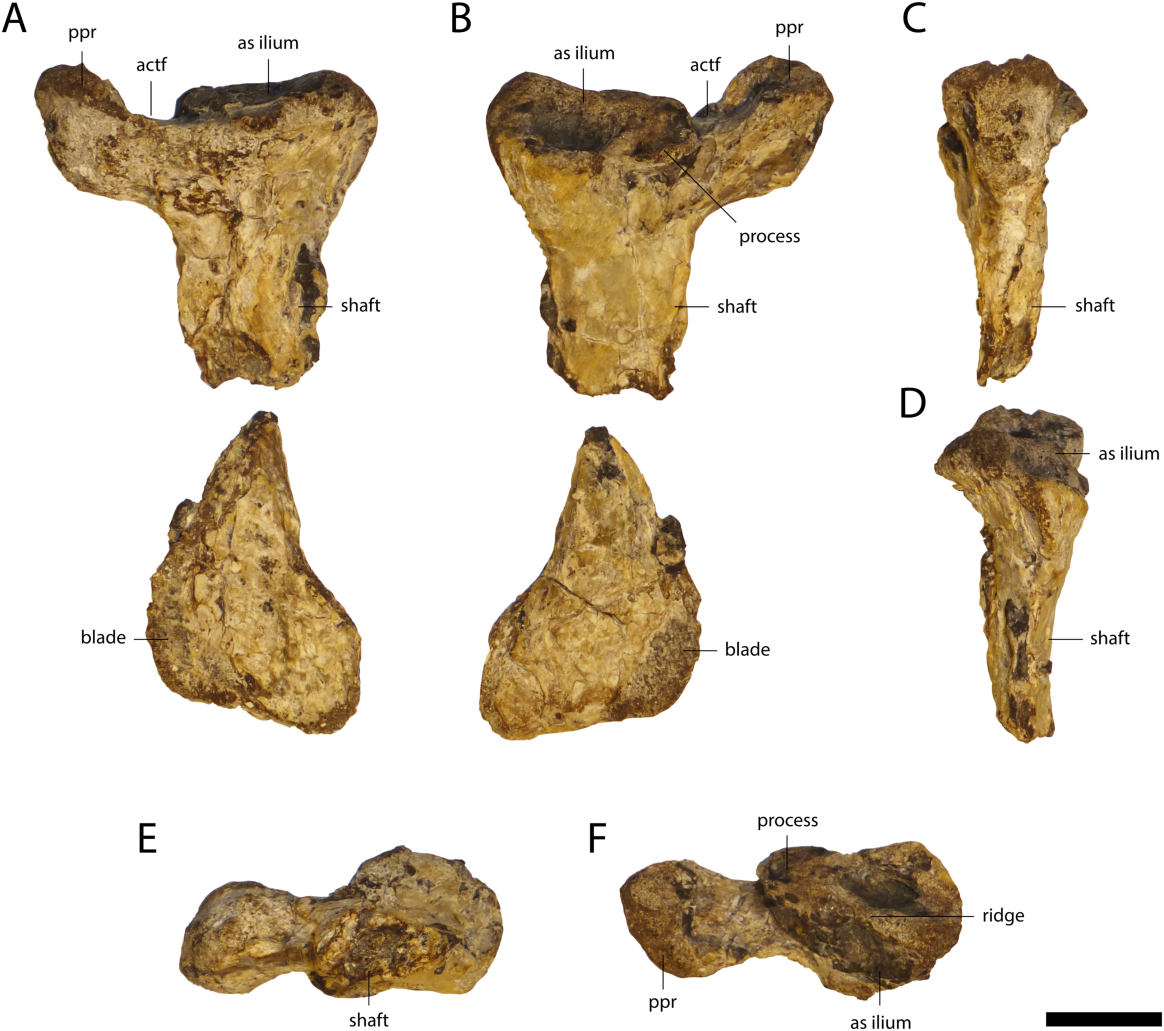
Ischium of *Orientalosuchus naduongensis* (GPIT/RE/09761) (holotype), Na Duong Formation, upper Eocene, Vietnam. Left ischium in lateral (A), medial (B), anterior (C), posterior (D), ventral (E) and dorsal (F) view. Abbreviations: as, articulation surface; actf, acetabulum foramen; ppr, pubic process. Scale = 1 cm.

The best preserved ischium is the left one of GPIT/RE/09761 (Fig. 18). Its proximal region has two articulation surfaces, separated from each other by the ventral part of the acetabulas foramen. The iliac process is approximately four times larger than the pubic process. The articulation surface with the ilium is oval, broad and has a shallow posterolaterally projecting ridge. The proximal part of the pubic process is oval and lateromedially oriented. The anterior edge of the shaft is bowed and the blade projects posteriorly.

### Pubis

A single left pubis is preserved in GPIT/RE/09784. Its articulation surface with the ischium is oval and anteroposteriorly elongated. The shaft is very thin and the blade flares strongly anteroposteriorly.

### Femur

**Figure 19:**
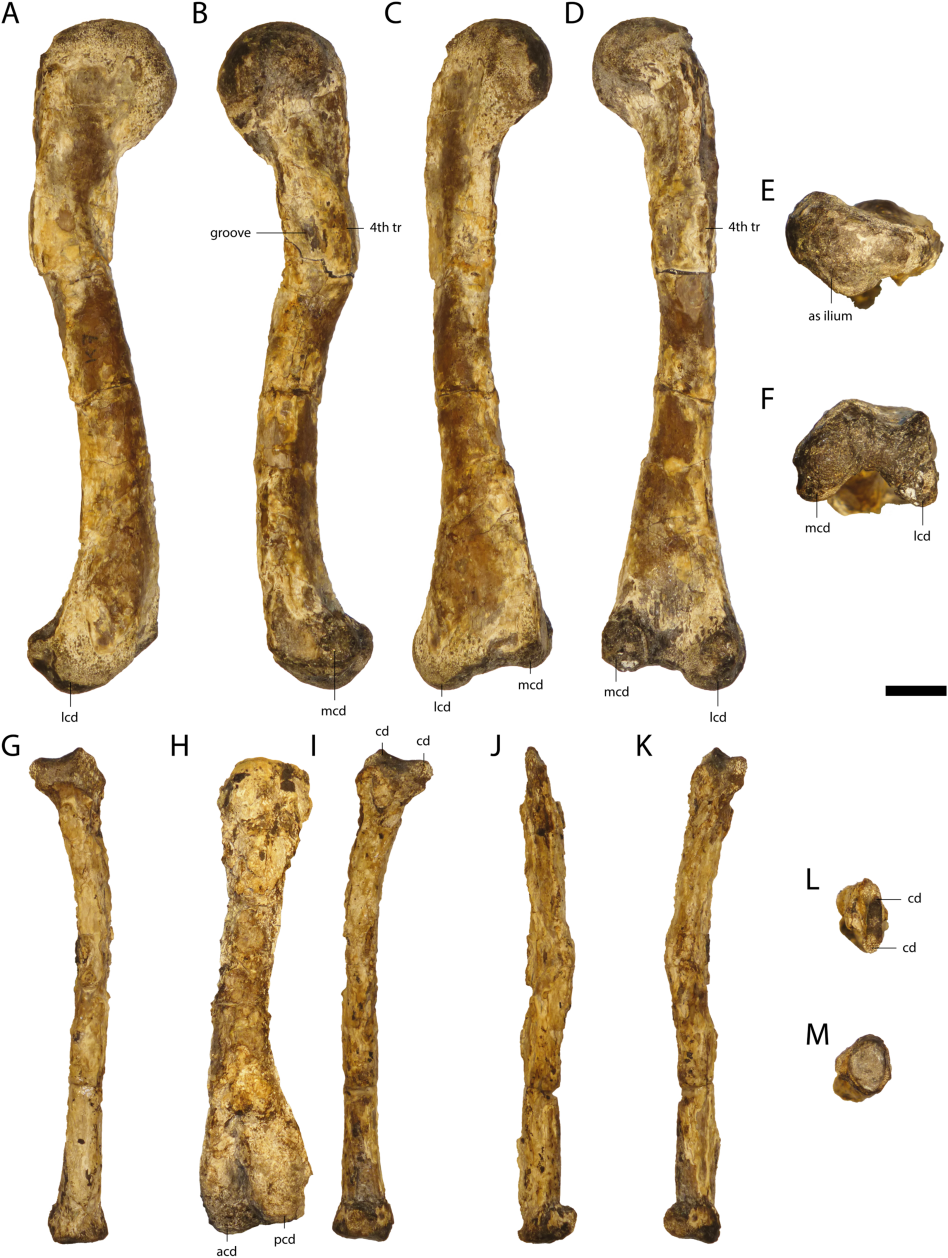
Femur and fibula of *Orientalosuchus naduongensis* (GPIT/RE/09761) (holotype), Na Duong Formation, upper Eocene, Vietnam. Right femur in lateral (A), medial (B), dorsal (C), ventral (D), proximal (E), and distal (F) view. Right fibula in lateral (G), medial (I), dorsal (J), ventral (K), proximal (L) and distal (M) view. Right tibia in medial (H) view. Abbreviations: 4th tr, fourth trochanter; acd, anterior condylus; as, articulation surface; cd, condylus; lcd, lateral condylus; mcd, medial condylus; pcd, posterior condylus. Scale = 1 cm.

The femur of GPIT/RE/09761 (Fig. 19) is 112.8 mm in length, while the femora of GPIT/RE/09784 and GPIT/RE/09727 are 110.8 and 109.2 mm, respectively. The femur is slightly sigmoidal with the proximal head lateromedially flattened and anteriorly broader than posteriorly. On the convex medial region, the head forms an articular surface with the acetabulum of the ilium. The smooth shaft has a prominent fourth trochanter on its medial side. Anteriorly to the fourth trochanter, a large groove is present. The distal end of the femur consists of the larger lateral and the smaller medial condyles with an intercondylar groove between them.

### Tibia/Fibula

The tibia and fibula are best preserved in GPIT/RE/09761 (Fig. 19) and GPIT/RE/09784. The tibia of GPIT/RE/09784 is 83.3 mm in length and has a slightly bowed shaft. Its proximal articulation surface is broad, whereas the distal one is narrow. Medially on the proximal epiphysis, a deep sulcus is present. The fibula of this individual is with the length of 78.1 mm, slightly smaller than the tibia and very thin.

### Astragalus/Calcaneum

The left astragalus and calcaneum are preserved in GPIT/RE/09784 and show no noticeable difference from other alligatoroids.

### Osteoderms

**Figure 20:**
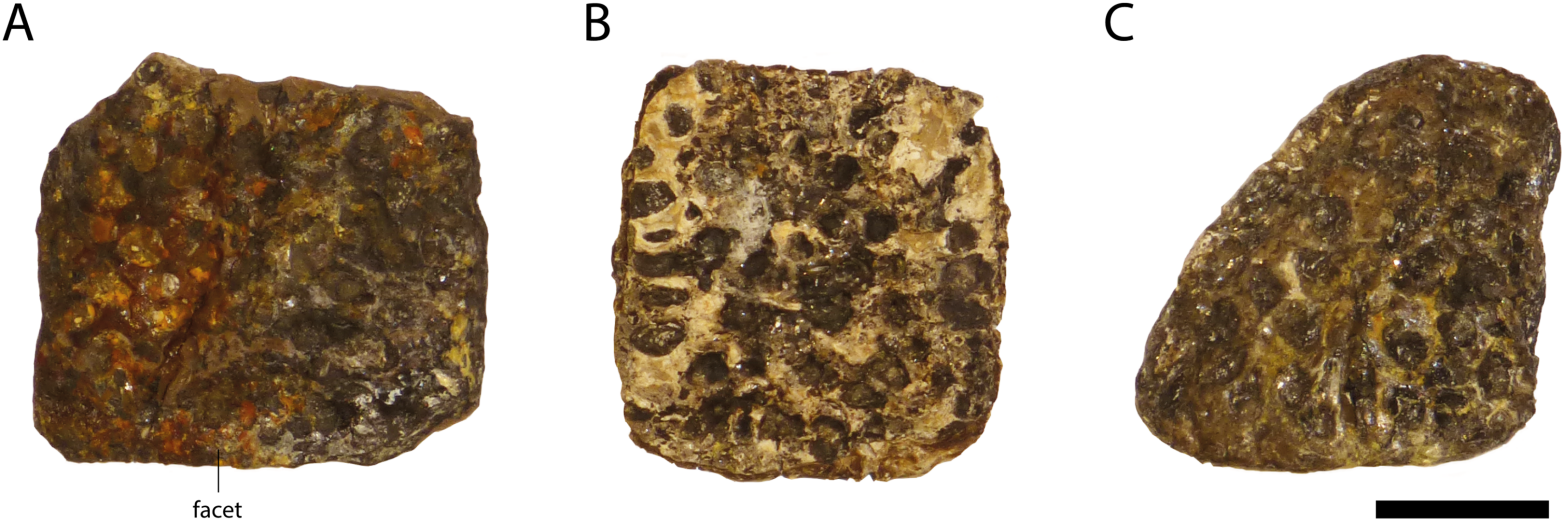
Osteoderms of *Orientalosuchus naduongensis* (GPIT/RE/09761) (holotype), Na Duong Formation, upper Eocene, Vietnam. Probable dorsal osteoderm (A and B) and a probable anterolateral osteoderm (C). Scale = 1 cm.

More than 150 osteoderms (Fig. 20) and osteoderm fragments are preserved, but most are in poor condition. All of them are ornamented with small rounded pits. Most of the osteoderms are disarticulated, but some are still in contact with the vertebra column and as such can be associated with *Orientalosuchus naduongensis*.

Most of the preserved osteoderms of GPIT/RE/09761 are dorsals. They are nearly square-shaped and possess no, or only a very shallow keel. This is also true for the posterodorsal midline osteoderms of GPIT/RE/09784.

Another osteoderm type, probably more posterolaterally located and very well preserved (GPIT/RE/09727) is relatively small and oval. These osteoderms show still weak, but slightly more pronounced keel than the dorsal osteoderms.

A single osteoderm of GPIT/RE/09761 is roughly triangular and could belong to the anterolateral region. No keel is visible, but the surface is weathered.

## Results of phylogenetic analysis

**Figure 21:**
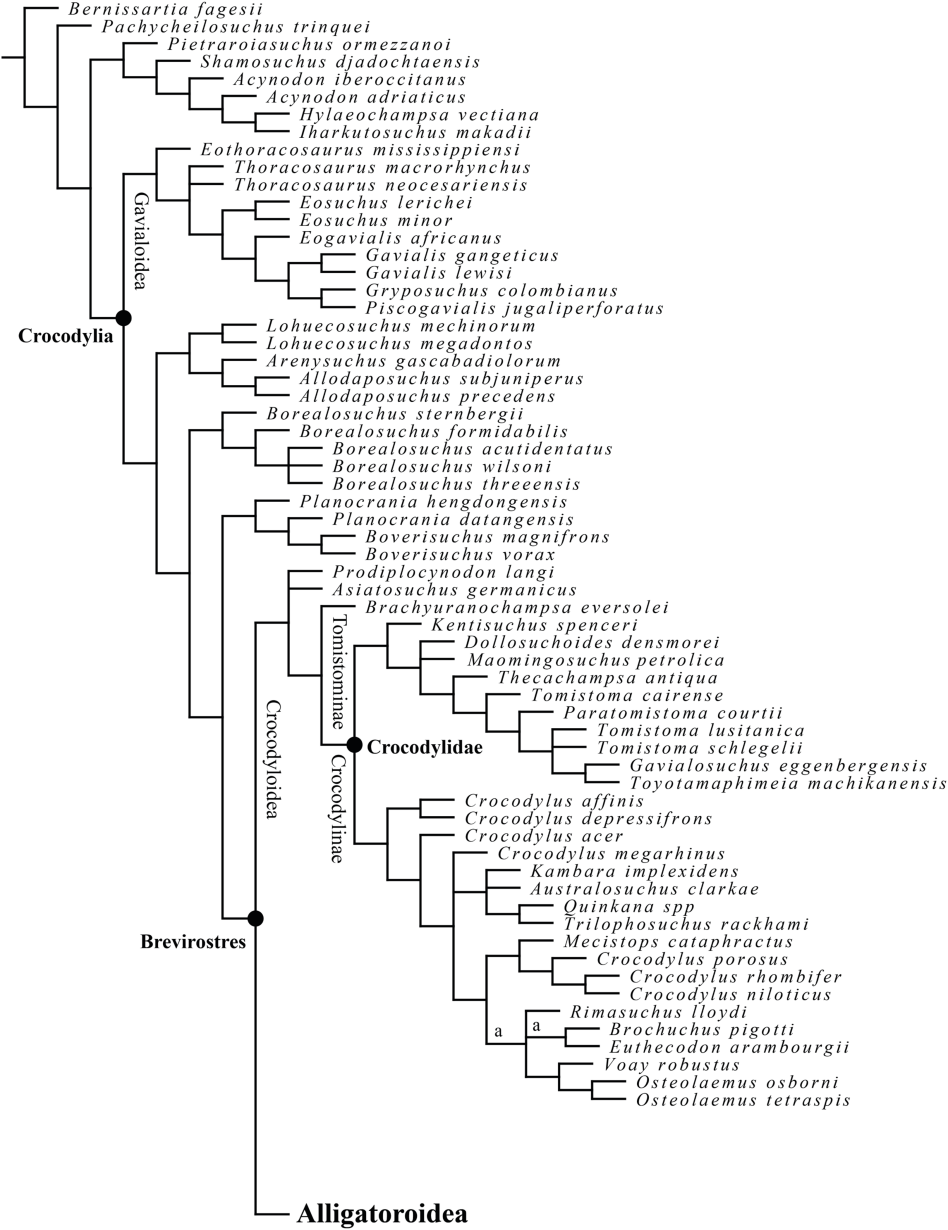
Reduced strict consensus tree of 20,160 equally optimal trees, obtained from the maximum parsimony analysis with 202 characters included; length: 927; CI: 0.292 and RI: 0.759. “a” indicates the alternative position of the pruned “Maoming alligatoroid”.

**Figure 22:**
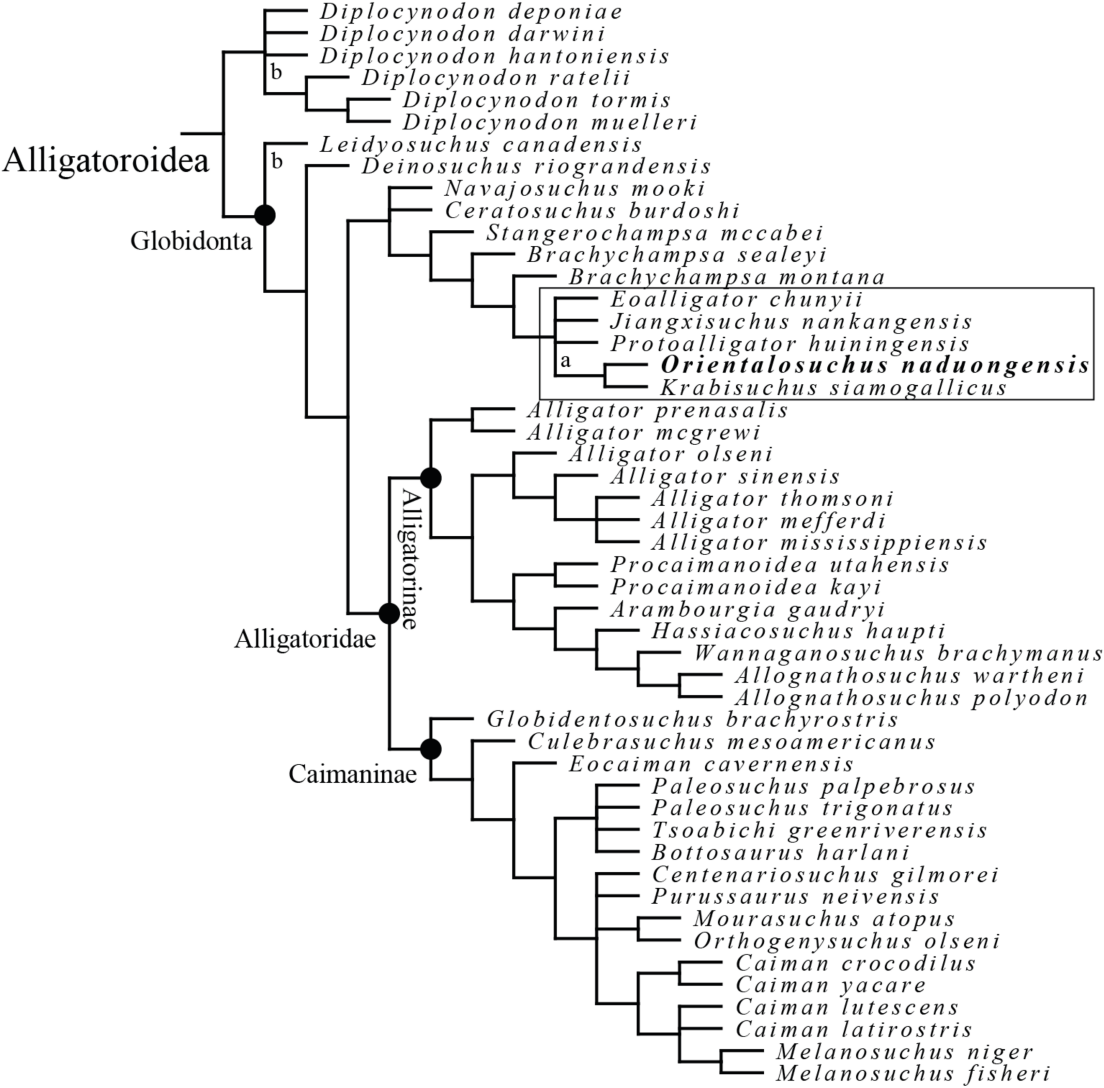
Alligatoroid phylogeny as inferred from the reduced strict consensus tree of 20,160 equally optimal trees, obtained from the maximum parsimony analysis with 202 characters included; length: 927; CI: 0.292 and RI: 0.759. “a” indicates the alternative position of the pruned “Maoming alligatoroid”. “b” indicates the alternative position of the pruned *Asiatosuchus nanlingensis*. The box highlights the monophyletic Orientalosuchina.

A total of 20,160 equally optimal trees with a length of 927 steps were recovered, with a consistency index (CI) of 0.292 and a retention index (RI) of 0.759 (Figs. 21 and 22). Two taxa (the Maoming alligatoroid and *Asiatosuchus nanlingensis* Young, 1964) were pruned from the strict consensus tree, because of their unstable position on the tree. Due to the expansion and modification of previous matrices, the retrieved trees differ from that of previous analyses (Brochu, 2007a, 2007b; Brochu & Storrs, 2012). A list of synapomorphies can be found in the File S2.

Outside Brevirostres, the monophyletic *Arenysuchus gascabadiolorum* Puértolas, Canudo & Cruzado-Caballero, 2011 + *Allodaposuchus subjuniperus* Puértolas-Pascual, Canudo & Moreno-Azanza, 2013 + *Allodaposuchus precedens* Nopcsa, 1928 + *Lohuecosuchus mechinorum* Narváez et al., 2015 + *Lohuecosuchus megadontos* Narváez et al., 2015 group is no longer found as the sister group to Hylaeochampsidae, but as sister group to all other Crocodylia except Gavialoidea. Omitting one of the new characters **(199)** from the analysis results in the previous sister group relationship outside Crocodylia.

The polytomy between *Pachycheilosuchus trinquei* Rogers, 2003, *Pietraroiasuchus ormezzanoi* Buscalioni et al., 2011 and *Shamosuchus djadochtaensis* Mook, 1924a (Narváez et al., 2015), was solved in the current tree. *Pietraroiasuchus ormezzanoi* and *Shamosuchus djadochtaensis* now form a monophyletic group with *Acynodon iberoccitanus* Buscalioni, Ortega & Vasse, 1997, *Acynodon adriaticus* Delfino, Martin & Buffetaut, 2008, *Hylaeochampsa vectiana* Owen, 1874 and *Iharkutosuchus makadii* Ősi, Clark & Weishampel, 2007. The character **(196)** supports this monophyletic group as all of these taxa, except *Hylaeochampsa vectiana*, in which the state is unknown, lack a notch between the premaxilla and maxilla as an adult (196-1). *Pachycheilosuchus trinquei* is the sister taxon to the clade formed by these taxa and all Crocodylia.

The clade *Crocodylus depressifrons* Blainville, 1855 + *Crocodylus affinis* Marsh, 1871, representing basal members of Crocodyloidea in previous studies (Delfino & Smith, 2009; Brochu & Storrs, 2012; Conrad et al., 2013), were retrieved in a weakly supported more derived position, as a basal member of Crocodylinae. One of the characters responsible for the more derived position of *Crocodylus depressifrons* + *Crocodylus affinis* is their short dentary symphysis (49-0), similar to most Crocodylidae, whereas the symphysis is long (49-1) in *Asiatosuchus germanicus* Berg, 1966. The character (142; posterior angle of the infratemporal fenestra), on the contrary, points towards a close relationship with *Asiatosuchus germanicus* and a more basal position (142-0). By adding a new character **(197)**, *Crocodylus depressifrons* and *Crocodylus affinis* are now drawn closer to Crocodylidae, but it is worth to mention that the character can be only scored for *Crocodylus depressifrons* (short sutural contact of the exoccipitals dorsal to the foramen magnum; 197-1).

In former analyses (Brochu, 2007b; Brochu & Storrs, 2012), the clade *Crocodylus acer* Cope, 1882 + *Brachyuranochampsa eversolei* Zangerl, 1944 was found in a sister group relationship with Crocodylidae. In the current phylogeny, *Crocodylus acer* was moved to a more derived position inside Crocodylinae. The reason for this is, that the scorings for the new characters are identical in *Crocodylus acer* and *Mecistops cataphractus* (Cuvier, 1825) and similar to other Crocodylinae, while *Brachyuranochampsa eversolei* could not be scored for these new characters.

*Paratomistoma courti* Brochu & Gingerich, 2000 was found as the sister taxon to the monophyletic group consisting of *Tomistoma lusitanica* Antunes, 1961, *Tomistoma schlegelii* (Müller, 1838), *Toyotamaphimeia machikanensis* (Kobatake et al., 1965) and *Gavialosuchus eggenburgensis* Toula & Kail, 1885 in the current analysis. In previous analyses (Brochu & Storrs, 2012; Conrad et al., 2013; Wang, Sullivan & Liu, 2016), *Paratomistoma courti* was found in a monophyletic group with *Maomingosuchus petrolica* (Yeh, 1958) and *Penghusuchus pani* Shan et al., 2009. *Penghusuchus pani* was not included in the current analysis, which most likely led to the new position of *Paratomistoma courti* on the tree.

*Leidyosuchus canadensis* has been considered the most basal alligatoroid (Brochu, 1999, 2004; Martin & Lauprasert, 2010; Wang, Sullivan & Liu, 2016). In the current analysis, however, *Leidyosuchus canadensis* is positioned more crown-ward and Diplocynodontinae is found as sister group to all other Alligatoroidea. This result is due to one of the new characters: the anterior jugal process placed at the same level **(195-1)** or posterior **(195-2)** to the anterior frontal process in Diplocynodontinae (only preserved in *D. tormis* Buscalioni, Sanz & Casanovas, 1992 and *D. muelleri* (Kälin & Peyer, 1936)), as opposed to *Leidyosuchus canadensis*, in which the jugal processes is anterior to the frontal **(195-0)**.

*Navajosuchus mooki* (Simpson, 1930) and *Ceratosuchus burdoshi* Schmidt, 1938 are outside of Alligatoridae, while in previous analyses they were unresolved at the base of Alligatorinae (Brochu, 1999, 2004; Cossette & Brochu, 2018). In the current analysis, they form a polytomy with a monophyletic group consisting of *Stangerochampsa mccabei*, *Brachychampsa sealeyi* Williamson, 1996, *Brachychampsa montana* and Orientalosuchina.

Orientalosuchina is supported by one synapomorphy (see “Discussion”). Inside Orientalosuchina, *Orientalosuchus naduongensis* and *Krabisuchus siamogallicus* form a monophyletic group, which in turn forms a polytomy with *Eoalligator chunyii*, *Jiangxisuchus nankangensis* and *Protoalligator huiningensis*.

In the present analysis, a broad scapulacoracoid facet immediately anterior to the glenoid fossa (26-1) and nearly squared dorsal midline osteoderms (39-1) defines Alligatoridae and three characters define Alligatorinae: eight contiguous dorsal osteoderms per row at maturity (40-2), a premaxilla with a deep notch lateral to the naris (86-1) and a longer prefrontal than the lacrimal (130-1).

The Bremer support for Orientalosuchina is 1 and the absolute frequency Bootstrap value is 5%. The Bremer support for *Orientalosuchus naduongensis* + *K. siamogallicus* is 1 and the absolute frequency bootstrap value is 35%. If the poorly preserved *Protoalligator huiningensis* is removed from the analysis, the absolute frequency bootstrap value of Orientalosuchina goes up to 45% and further removing the also poorly preserved *Eoalligator chunyii* results in a bootstrap value of 54% for Orientalosuchina and 51% for *Orientalosuchus naduongensis* + *K. siamogallicus*.

## Discussion

### Phylogeny of Globidonta

Globidonta is better resolved in the current analysis compared to previous studies with East and Southeastern Asian alligatoroids included (Skutschas et al., 2014; Wang, Sullivan & Liu, 2016; Wu, Li & Wang, 2018). Statistical support (Bremer and Bootstrap), however, remains low. The group has three synapomorphies: a lingual foramen for articular artery and alveolar nerve perforates surangular entirely (69-0), a concavo-convex frontoparietal suture (151-0) and an anterior jugal process extending anterior to the anterior process of frontal (195-0), but none is unique for this group.

Omitting the new characters **194**, **195**, **197** and **199**, results in a similar phylogeny in most non-globidontan taxa as in former analyses (Brochu, 1999, 2007b; Brochu & Storrs, 2012; Cossette & Brochu, 2018), but the resolution is reduced inside Tomistominae and Alligatorinae.

### Orientalosuchina

Orientalosuchina, includes *Orientalosuchus naduongensis* from the middle to late Eocene of Vietnam, *Krabisuchus siamogallicus* from the late or latest Eocene of Thailand (Benammi et al., 2001; Martin & Lauprasert, 2010), *Protoalligator huiningensis* from the middle Paleocene of Southeast China, and *Eoalligator chunyii* and *J. nankangensis* from the Late Cretaceous-early Paleocene of Southeast China.

In all trees, the single synapomorphy of Orientalosuchina is a short dentary symphysis extending to the height of the fourth to fifth alveolus (49-0) as opposed to the long symphysis (49-1) of for example, *Brachychampsa* spp. and *Stangerochampsa mccabei*. A short symphysis is otherwise common for alligatoroids (most *Alligator* spp. and most Caimaninae).

Inside Orientalosuchina, *J. nankangensis*, *Eoalligator chunyii*, *Protoalligator huiningensis* and *Orientalosuchus naduongensis* + *K. siamogallicus* form a polytomy. In those trees in which *Protoalligator huiningensis* is ancestral, the presence of a notch between the premaxilla and maxilla (196-0) is a further synapomorphy for the group.

If *Eoalligator chunyii* is recovered as the most basal taxon, the monophyly of Orientalosuchina is further supported by seven synapomorphies:

(1) a truncated surangular around the lateral wall of the glenoid fossa (67-1); (2) the medial position of the foramen aerum on the retroarticular process (70-0) and (3) the quadrate (177-0); (4) a very short anterior palatine process, not reaching the anterior end of the suborbital fenestra (115-1); (5) the quadratosquamosal suture extends dorsally along the caudal margin of the external auditory meatus (148-1); (6) the squamosal extends ventrolaterally to lateral extent of paraoccipital process (159-1); and (7) the 11th dentary tooth is the largest one after the fourth one (200-0).

Further synapomorphies may diagnose Orientalosuchina including: (1) the posterior lateral edges of the palatines are parallel (120-0); (2) the lacrimal makes broad contact with the nasal without any sign of a maxillary process (128-0); (3) the anterior tip of the frontal is acute (131-0); (4) the postorbital does not contact the quadrate and quadratojugal at the mediodorsal angle of the infratemporal fenestra (143-0); and (5) the angular-surangular suture lingually originates near the dorsal border of the external mandibular fenestra and is straight (201-1). However, given that the corresponding anatomical regions are unknown in the basally branching and poorly preserved taxa these now define a more inclusive clade.

Consistent with their basal divergence and age, orientalosuchians retain several crocodylian plesiomorphies, which may explain their recovery inside Crocodyloidea in previous analyses (Wang, Sullivan & Liu, 2016; Wu, Li & Wang, 2018; Li, Wu & Rufolo, 2019). These characters include: (1) and (2) the medially located foramen aerum (70-0) (177-0); (3) the fifth maxillary tooth is the largest one (93-1); (4) the anterior palatine process does not extend anterior to the suborbital fenestra (115-1); (5) the lateral edges of palatines are posteriorly parallel (120-0); (6) an acute anterior frontal tip (131-0); (7) a postorbital neither contacting the quadrate nor the quadratojugal medially at the dorsal angle of the infratemporal fenestra (143-0); (8) the quadratosquamosal suture extends dorsally along the caudal margin of the external auditory meatus (148-1); (9) the squamosals extend ventrolaterally to the lateral extent of the paraoccipital process (159-1); and (10) the presence of a notch between the premaxilla and maxilla (196-0).

### Relationships inside Orientalosuchina

*Orientalosuchus naduongensis* and *Jiangxisuchus nankangensis* share a very similar pterygoid around the choana opening (123-2), which strongly resembles the morphology of *Osteolaemus tetraspis* Cope, 1861 and *Voay robustus*. The choanal region in *Krabisuchus siamogallicus* (though not intact) looks like the pterygoid surface is flush with the choanal margin (123-0). In *Eoalligator chunyii* and *Protoalligator huiningensis* the pterygoid is not preserved. The pterygoid forming a neck around the choana is a condition unknown for any other alligatoroid and could be a further synapomorphy for Orientalosuchina with a potential reversal in *K. siamogallicus*.

A further potential synapomorphy for the group is the axial hypapophysis located towards the centrum of the axis body (15-1) in *Orientalosuchus naduongensis* and *Eoalligator chunyii*. The axis is, unfortunately, not preserved in any other members of Orientalosuchina. A shifted axial hypapophysis among Crocodylia is otherwise only present in Diplocynodontinae and *Crocodylus depressifrons*.

The clade of *Orientalosuchus naduongensis* + *K. siamogallicus* has no synapomorphies in all trees, but has five in some trees including (1) the intersupratemporal bar being similarly broad as the supratemporal fenestra (199-1); (2) the dentary curves deeply between the fourth and 10th dentary alveoli (50-1); (3) a palatine-pterygoid-suture situated far from the posterior angle of the suborbital fenestra (118-1); (4) the frontoparietal suture lies entirely on the skull table (150-2); and (5) the anterior and medial teeth have dominant vertical ridges on their labial surface (198-1) and to a lesser extent on their mesial side. Such strong ridges are only present in few crocodylians (*Allodaposuchus precedens*, *Allodaposuchus subjuniperus* and *Maomingosuchus petrolica*), but not in other member of Alligatoroidea.

Martin & Lauprasert (2010) described *K. siamogallicus* as having unusually long lacrimals, reaching the premaxilla and as a result preventing the maxilla from contacting the nasal. These sutures are more likely the preorbital ridges (97-1) also present in *Orientalosuchus naduongensis*. These ridges may be a further autapomorphy for *Orientalosuchus naduongensis* or a synapomorphy for *Orientalosuchus naduongensis* + *K. siamogallicus*. Prominent preorbital ridges are otherwise only present in *Mourasuchus atopus* Langston, 1966 among alligatoroids and in some members of Crocodyloidea, like in *Osteolaemus tetraspis* and *Crocodylus porosus*, but these structures are more prominently developed in *Orientalosuchus naduongensis*.

*Orientalosuchus naduongensis* is characterized by a very large supraoccipital exposure on the skull table (160-3), whereas the exposure is large (160-2) in *Eoalligator chunyii* and *K. siamogallicus*, but small (160-0) in *J. nankangensis*. The state of this character for *Protoalligator huiningensis* is unknown. A very large supraoccipital exposure is otherwise found in some Caimaninae, but in most of those taxa, except for *Globidentosuchus brachyrostris*, the supraoccipital is not trapezoid-shaped but block-shaped.

Omitting *Protoalligator huiningensis* from the phylogenetic analysis results in a sister taxon relationship between *J. nankangensis* and *Eoalligator chunyii*, forming a sister group to *Orientalosuchus naduongensis* + *K. siamogallicus*. A close relationship between the two Late Cretaceous/early Paleocene species *J. nankangensis* and *Eoalligator chunyii* was also recently found in the analysis of Li, Wu & Rufolo (2019), although the clade was nested inside of Crocodyloidea.

### *Brachychampsa* spp., *Stangerochampsa mccabei* and Orientalosuchina

In some trees, the monophyly of *Stangerochampsa mccabei* + *Brachychampsa* spp. + Orientalosuchina is supported by: (1) a dorsally pojecting naris (81-1), (2) a large incisive foramen, intersecting the premaxillary-maxillary suture (88-2), (3) a maxilla with posterior process between lacrimal and prefrontal (128-2) and (4) a frontoparietal suture making modest entry into the supratemporal fenestra (150-1).

The monophyly of *Brachychampsa* spp. + Orientalosuchina is supported by: (1) the fifth maxillary alveolus as the largest (93-1) and (2) a large supraoccipital exposure on the skull table (160-2).

In basal members of Alligatoroidea (*Diplocynodon* spp. and *Leidyosuchus canadensis*), the fourth and fifth maxillary teeth have the same size (93-3), whereas it is the fourth tooth (93-2) in members of Alligatoridae. The only exception is the Late Cretaceous/early Paleocene *Bottosaurus harlani*, although position of this taxon inside Caimaninae is questionable (Cossette & Brochu, 2018).

The monophyly of *Brachychampsa montana* + Orientalosuchina is supported by the anterior tip of the splenial passing ventrally to the Meckelian groove (54-1).

A closer relationship between *Brachychampsa montana* and Orientalosuchina than between *Brachychampsa montana* and *Brachychampsa sealeyi* seems unlikely and could be an artifact due to the poor preservation of the latter.

### *Navajosuchus mooki* and *Ceratosuchus burdoshi*

The shift to a more basal position of *N. mooki* and *Ceratosuchus burdoshi* compared to Alligatoridae in this study can be explained by: (1) a straight dentary (50-0), (2) the presence of a large incisive foramen (88-1), and (3) a relatively flat skull in lateral view (193-0) unlike in the monophyletic group consisting of *Arambourgia gaudryi* (De Stefano, 1905) + *Hassiacosuchus haupti* + *Wannaganosuchus brachymanus* Erickson, 1982 + *Allognathosuchus* spp..

### *Allognathosuchus* spp.

The monophyly of *Allognathosuchus* spp. is supported by: (1) a surangular-dentary suture intersecting the external mandibular fenestra at the posterodorsal corner (64-1) and (2) an anterior jugal process extending anterior to the frontal (195-0). In *Wannaganosuchus brachymanus*, *Arambourgia gaudryi* and *Hassiacosuchus haupti*, the anterior jugal process lies at the same height as the anterior frontal process (195-1).

### *Alligator* spp.

In the present phylogeny, *Alligator* is polyphyletic with *Alligator prenasalis* (Loomis, 1904) + *Alligator mcgrewi* Schmidt, 1941 outside the other *Alligator* species due to a long dentary symphysis (49-1). They further differ in: (1) an anterodorsally projecting naris (81-0), (2) a lingual foramen for the articular artery and alveolar nerve perforating the surangular/angular suture (69-1) and (3) a maxilla bearing a broad shelf extending into the suborbital fenestra (112-1). The last two characters, however, can also be found in other *Alligator* species (*Alligator mississippiensis*, *Alligator mefferdi* Mook, 1946 and *Alligator sinensis*).

### Caimaninae

*Eocaiman caverensis* Simpson, 1933 is found as the sister taxon to most of other caimans with the exception of the Miocene *Globidentosuchus brachyrostris* and *Culebrasuchus mesoamericanus*, which are found in an ancestral position in the current analysis, with *Globidentosuchus brachyrostris* as the most basal caiman.

This basal position of *Globidentosuchus brachyrostris* results mainly from (1) the splenial participating in the mandibular symphysis (54-0), (2) a concavoconvex frontoparietal suture (151-0) and (3) a trapezoid-shaped supraoccipital exposure on the skull table (202-0). *Globidentosuchus brachyrostris* and *Culebrasuchus mesoamericanus* both differ from most of other Caimaninae in: (1) having an angular-surangular suture contacting the external mandibular fenestra at the posterior angle (60-0) and (2) an exoccipital terminating dorsally to the basioccipital tubers (176-0) in *Culebrasuchus mesoamericanus*, whereas this is unknown for *Globidentosuchus brachyrostris*. Considering the Miocene age of both taxa, either they have a ghost lineage reaching back to the Cretaceous, or their basal position is an artifact due to incompleteness of those taxa.

As in Cossette & Brochu (2018), the Late Cretaceous/early Paleocene *Bottosaurus harlani* was found in a polytomy with *Paleosuchus* spp.. In the present analysis, however, *Tsoabichi greenriverensis* Brochu, 2010 was also found inside this polytomy. The reason for this is the new character **(202)**. In *Tsoabichi greenriverensis* and *Paleosuchus* spp., the large supraoccipital exposure is triangular (202-1), while it is block-shaped (202-2) or unknown for the remaining caimans, including all recent species.

### Taxonomic status of the Maoming alligatoroid

Two taxa were pruned from the consensus tree because of their unstable positions: the fragmentary *Asiatosuchus nanlingensis* and the Maoming alligatoroid. The Maoming taxon (Skutschas et al., 2014) is either the sister taxon to *Orientalosuchus naduongensis* or placed inside Crocodylinae. This is due to the poor character support, resulting in the same character combination for the Maoming alligatoroid as that of the crocodyline *Brochuchus pigotti* (Tchernov & van Couvering, 1978).

*Orientalosuchus naduongensis* and the Maoming alligatoroid closely resemble each other in the prominent ridges along the nasals, the triangular-shaped lacrimals and anteriorly shifted supratemporal fenestrae are marked similarities. The Maoming alligatoroid was interpreted as lacking a premaxilla-maxilla notch (Skutschas et al., 2014), but this is likely an artifact of deformation as indicated by a flattened specimen of *Orientalosuchus naduongensis* (Fig. 5) in which the notch appears absent even though better preserved specimens clearly reveal the presence of the notch in this species. The complex sutural contact between the nasal and frontal, proposed for the Maoming alligatoroid by Skutschas et al. (2014), differs from *Orientalosuchus naduongensis* but the area in the Maoming specimen is damaged and an anterior process of the frontal, similar to the one in *Orientalosuchus naduongensis*, could be assumed, making the taxa identical in this regard. These two taxa further share a close geographic and temporal proximity and we predict that they either represent the same taxon or are closely related to each other.

### Biogeographic implications

The Chinese alligator, *Alligator sinensis*, is the only recent alligatoroid in Asia and the timing and climatic context of its dispersal from North America to Asia is still unresolved (Brochu, 1999; Snyder, 2007; Oaks, 2011; Shan, Cheng & Wu, 2013; Wang, Sullivan & Liu, 2016). Martin & Lauprasert (2010) were the first to include an Asian alligatoroid (*Krabisuchus siamogallicus*), other than *Alligator sinensis* into a phylogeny, followed by Skutschas et al. (2014) (Maoming alligatoroid), Wang, Sullivan & Liu (2016) (*Eoalligator chunyii* and *Protoalligator huiningensis*) and Li, Wu & Rufolo (2019) (*Jiangxisuchus nankangensis*).

Our phylogenetic analysis robustly places Orientalosuchina including all the above taxa distantly from *Alligator sinensis* and their morphology as well as their Cretaceous origin (*Eoalligator chunyii* and *J. nankangensis*) strongly suggest that they represent a more basal monophyletic clade of alligatoroids. East and Southeastern Asia was, therefore, colonized by alligatoroids twice: once by Orientalosuchina during the Late Cretaceous and once by the *sinensis* lineage during the Cenozoic (Brochu, 1999). For both lineages, dispersal via Beringia is the most consistent with paleogeography, climate, phylogeny, inferred stenohalinity, the fossil record, and divergence dates (Taplin & Grigg, 1989; Brochu, 1999; Fiorillo, 2008; Oaks, 2011; Li, Wu & Rufolo, 2019). Inferred late Maastrichtian lower eustatic sea level (Kominz et al., 2008) would have favored the dispersal of Orientalosuchina, which is consistent with the probable age of *Eoalligator chunyii* and *J. nankangensis*. Evidence for Late Cretaceous vertebrate dispersal from North America to Asia is otherwise scarce and include some tyrannosauroid, hadrosaurid, and ceratopsian dinosaurs (Loewen et al., 2013; Farke et al., 2014; Prieto-Márquez et al., 2019). Dispersal from Asia to North America, on the other hand, has been more commonly inferred for the Late Cretaceous (Russell, 1993; Hutchison, 2000; Sereno, 2000; Godefroit, Bolotsky & Alifanov, 2003). The relationships of the Late Cretaceous *Asiatosuchus nanlingensis* from Asia and *Prodiplocynodon langi* from North America are yet to be resolved and, therefore, alligatoroids are so far the only crocodylians showing migration from North America to Asia during this time.

Timing of the dispersal of the *sinensis* lineage remains difficult to constrain because: (1) molecular divergence date estimates and fossil dates of crown-*Alligator* are in conflict, (2) pan-*sinensis* in Asia cannot be traced back further than the Pliocene (Iijima, Takahashi & Kobayashi, 2016), and (3) the dispersal may have occurred long after a possible North American divergence between *Alligator sinensis* and *mississippiensis*. The most recent molecular divergence date estimate placed the split between *Alligator sinensis* and *mississippiensis* at ≈58–31 Ma (Oaks, 2011) as opposed to 14 Ma suggested by the earliest known fossil record of crown-*Alligator* (*Alligator thomsoni* Mook & Thomson, 1923, Brochu, 1997, this study). The early Miocene *Alligator olseni* White, 1942 is also close to crown-*Alligator* (Brochu, 1999; Snyder, 2007, this study) and, thus, ca. 20 Ma can be considered the maximum divergence date of the lineage based on fossils. Even though the molecular data sampling of Oaks (2011) is using a multilocus sequence dataset of both mtDNA and nDNA, similar analyses have been subjected to overestimate shallow nodes (<10 MY), particularly when they are dated with old external priors (Van Tuinen & Torres, 2015). Given that both calibration points of Oaks (2011) are deep (Alligatorinae-Caimaninae split, 71–64 Ma; Crocodylia, 90 Ma), a potential overestimation of *Alligator* divergence should be taken into account. Climate obviously constrained *Alligator* dispersal via Beringia (Markwick, 1998) but a revised molecular clock analysis using shallower calibration points and the inclusion of the Chinese Miocene *Alligator luicus* is critical for evaluating the more precise role it played.

## Conclusions

Parsimony analysis finds the new late Eocene taxon from Vietnam, *Orientalosuchus naduongensis*, as the sister taxon to *Krabisuchus siamogallicus* from the Eocene of Thailand. Together they form a monophyletic extinct basal East to Southeastern Asian alligatoroid clade of Late Cretaceous origin that also included *Jiangxisuchus nankangensis*, *Eoalligator chunyii* and *Protoalligator huiningensis*. The current phylogeny supports at least two different dispersals from North America to Eastern Asia: one during the Late Cretaceous (Orientalosuchina) and a second during the Cenozoic (*Alligator sinensis* lineage). Improved fossil calibrations and taxon sampling will be vital for further constraining the timing and resolving the climatic/paleogeographical context of these dispersals.

## Appendix

### Modifications and new characters added to the characterlist of Brochu & Storrs (2012)

See Supplementary file 2 for further modifications and Supplementary file 3 for a complete character list.

**(51)** Largest dentary alveolus immediately caudal to fourth is (0) 13 or 14, (1) between 11 and 14 and a series behind it, (2) 11 or 12, (3) no differentiation, (4) behind 14, (5) 10. (modified from Brochu & Storrs (2012)).

***Comments*:** According to Brochu (2004:867), “this character expresses the enlarged rear dentition of some fossil alligatorids.” and “The exact position of the largest alveolus varies within species, but it is never in front of the thirteenth in most taxa, and is never behind the twelfth in crown-group caimans. Behind these, alveoli grow progressively smaller. But in some blunt-snouted forms, there is a third region of maximum diameter behind the thirteenth or fourteenth alveolus. This is where globular teeth erupt in those taxa bearing them-teeth erupting from the large 13th or 14th alveoli are still conical.”

We rephrased character state (1) of Brochu & Storrs (2012) from “largest dentary alveolus immediately caudal to fourth is 13 or 14 and a series behind it” to “between 11 and 14 and a series behind it” in order to score all taxa with enlarged posterior teeth, regardless the shape of the crown, with the same state. Previously, some taxa with a lower tooth count (e.g., *Hassiacosuchus haupti*) were scored with (1) despite the fact that their largest tooth was not the 13 or 14 because they also possess enlarged posterior teeth. In addition, taxa with enlarged posterior teeth that are compressed instead of globular were furthermore excluded previously from this state. For instance, in *Orientalosuchus naduongensis*, *Bottosaurus harlani*, *Procaimanoidea utahensis* (Gilmore, 1946) and *Procaimanoidea kayi* (Mook, 1941) the rear dentition consists of enlarged but laterally compressed teeth. Previously both species of *Procaimanoidea*, as well as *B. harlani* were scored with (2) as in recent *Alligator* spp. or *Caiman* spp., but here we score them as (1).

**(193)** Skull in lateral view relatively flat (0) or wedge-shaped (1) (new character).

***Comments*:** A triangular- or wedge-shaped skull in lateral view occurs in some short snouted taxa. The character is not fully correlated with the presence of an anterodorsally positioned external narial opening because some fossil taxa with an anterodorsally positioned naris have a relatively long and flat snout (e.g. *Navajosuchus mooki*). The character is somewhat problematic to score due to the often ocuring dorsoventrally deformation in most crocodilian fossils. In most taxa, the skull shows a sudden increase in height between the orbits giving the it a slide-like outline in lateral view. In wedge-shaped taxa, however, the snout region is nearly straight triangular from the narial opening to the skull table, clearly visible in some basal alligatorines like *Hassiacosuchus haupti*.

All speicmens of *Orientalosuchus naduongensis* are dorsoventrally compressed due to deformation, making a reliable scoring for this character impossible. The snout, however, looks more elongated than that of the typical wedge-shaped short snouted alligatorines. Martin & Lauprasert (2010) described *Krabisuchus siamogallicus* as having a similar morphology as *H. haupti* and the head of Kr-C-007 indeed looks wedge-shaped, although the preservation is suboptimal and it could be an artifact due to the apparent postmortem deformation.

**(194)** Nasal bone does (0) or does not (1) reach to the level of the orbita (new character).

***Comments*:** In *Alligator*, the nasal usually reaches the level of the orbits, but not in *Alligator sinensis* in which the nasal terminates anterior to the orbits in the herein analyzed individuals (SMNS 4915, IRSNB 13904-3487). *Hassiacosuchus haupti* and *Arambourgia gaudryi* have a far posteriorly reaching nasal bone, while it is shorter in all other basal alligatorines and *Navajosuchus mooki*. In crown-group Caimaninae, only *Melanosuchus niger* (Spix, 1825), *Orthogenysuchus olseni* Mook, 1924b and both *Paleosuchus* species have a nasal bone, reaching to the level of the orbits. In Orientalosuchina, this character can be scored for *Orientalosuchus naduongensis* and *Krabisuchus siamogallicus*, which have far posterior reaching nasal processes reaching the orbita.

**(195)** Anterior process of jugal extends anterior (0), lies at the same level as (1), or well posterior to the anterior process of frontal (2). (Modified from Jouve (2016) (174), Jouve et al. (2008) (174), Jouve (2004) (177)).

***Comments*:** In Crocodyila, the jugal shows different anterior extensions, which can be compared to the anterior extension of the frontal. In non-Brevirostres taxa, the anterior process of the jugal commonly lies posteriorly to the anterior frontal process, which is present in some species like *Procaimanoidea utahensis*, *Alligator mcgrewi* Schmidt, 1941 or *Paleosuchus palpebrosus* (Cuvier, 1807), but in most Brevirostres, the anterior jugal process either extends anterior or lies at the same level as the anterior process of the frontal. The prior version of this character from Jouve (2016) did not differentiate between (0) and (1), which led to the present modification of this character.

Among Globidonta, the jugal extends anteriorly to the frontal (0) in *Allognathosuchus polyodon* (Cope, 1873), *Allognathosuchus wartheni* Case, 1925, *Alligator sinensis*, *Alligator mississippiensis* and *Alligator prenasalis*, as well as in *Brachychampsa montana* and *Stangerochampsa mccabei* while it is on the same level (1) in *Hassiacosuchus haupti*, *Arambourgia gaudryi*, *Navajosuchus mooki*, *Wannaganosuchus brachymanus* and *Procaimanoidea kayi*. In crown-group Caimaninae, the process always reaches anterior to the frontal, except for *Paleosuchus palpebrosus* and *Melanosuchus niger*, in which the process lies on the same height as the frontal. For Orientalosuchina, only *Orientalosuchus naduongensis* could be reliably be scored as state (1).

**(196)** Notch between the premaxilla and maxilla present (0) or not present (1) in adult individuals (new character).

***Comments*:** A few characters in the matrix are referring to an early ontogenetic state of a taxon (10, 25, 60, 87, 91, 126 and 152). The reason for this is potential changes during ontogeny like the development of a notch between the premaxilla and maxilla late in ontogeny in *Caiman crocodilus* (Linnaeus, 1758) (Brochu, 1999).

However, several fossil taxa are scored for such “ontogenetic characters” even if no juvenile specimens are known.

We added a new character that pertains to the presence of the premaxillary-maxillary notch in adult individuals in order to allow consistent scoring of fossil taxa with unknwon juvenile stage. To avoid double weighting, character (91) was reviewed based on published work and if the scoring was solely based on adult individuals, they were rescored as (?) and the previous scoring was added to the new character (196).

**(197)** Sutural contact of the exoccipitals dorsal to the foramen magnum (0) long, at least half the height of the foramen magnum, (1) short, shorter than half the height of the foramen magnum, or (2) no sutural contact between the exoccipitals (new character).

***Comments*:** In recent *Crocodylus* and *Alligator* species, the supraoccipital does not project far ventrally, resulting in a relatively long suture between the two exoccipitals. In for example, *Osteolaemus tetraspis* and some globidonts (*Brachychampsa montana*, *Stangerochampsa mccabei*, *Hassiacosuchus haupti*, *Arambourgia gaudryi* and *Eoalligator chunyii*) the supraoccipital projects further ventrally and the sutural contact between the exoccipitals becomes smaller, until it is only roughly half as long as the maximal height of the foramen magnum. Unfortunately, in many fossil taxa, the occipital region is too poorly preserved or not figured to reliably score this character, but it could be valuable to understand wheter some basal *Alligator* species show a short suture and if there are differences between basal Alligatorinae taxa. Among recent Caimaninae, in *Paleosuchus palpebrosus* and *Melanosuchus niger* the suture is long and it is short in *Caiman crocodilus* and *Caiman latirostris* (Daudin, 1802). In Orientalosuchina, only *Eoalligator chunyii* could be scored (with a short suture).

In two non-Brevirostres taxa (*Allodaposuchus precedens* and *Borealosuchus sternbergii* (Gilmore, 1911)) the supraoccipital reaches the foramen magnum preventing the exoccipitals from contacting one another.

**(198)** Anterior maxillary teeth without (0) or with (1) ridges on their lateral surface (new character).

***Comments*:** In many crocodilians, the maxillary teeth bear fine striae, but in *Orientalosuchus naduongensis* and *Krabisuchus siamogallicus* there are marked dorsoventrally running ridges present on the labial surface of the anterior teeth, (unknown for *Eoalligator chunyii*). Dominant ridges are also present in the tomistomine *Maomingosuchus petrolica* and in *Allodaposuchus precedens* and *Allodaposuchus subjuniperus*.

**(199)** Intersupratemporal bar (0) as or near as broad as the supratemporal fenestra, (1) at least twice as broad as the supratemporal fenestra, (2) around half the broadness of the supratemporal fenestra or (3) constricted, less than half the broadness of the supratemporal fenestra (new character).

***Comments*:** The intersupratemporal region differs among crocodilians, but most commonly, the region is either similarly broad as the supratemporal fenestra or constricted, especially in longirostrine taxa. The level of constriction was divided in two states (2 and 3). A markedly broad state is only present in few taxa, especially in crown-group Caimaninae in which the supratemporal fenestrae are overgrown, but it is not completely correlated with (152) as a broad intersupratemporal bar is also present in *Procaimanoidea kayi*, which has open fenestrae.

Other globidonts mostly have an intersupratemporal bar as broad as the fenestra or a slender, but not constricted one. Exceptions are *Brachychampsa montana*, *Ceratosuchus burdoshi* and *Eoalligator chunyii*, which have a constricted bar.

**(200)** If largest dentary alveolus is between 11th and 14th and a series behind it, is it the (0) 11th, (1) 12th, or (2) 13th to 14th (new character).

***Comments*:** This character is only applicable for taxa with (51-1).

**(201)** Surangular-angular suture lingually originates (0) near the ventral border of the external mandibular fenestra, (1) near the dorsal border of the external mandibular fenestra and straight, (2) near the dorsal border of the external mandibular fenestra and bowed (new character).

***Comments*:** In most crocodylians, the surangular-angular suture lingually originates near the ventral border of the external mandibular fenestra (201-0). In Orientalosuchina, however, the suture originates near the dorsal border of the external mandibular fenestra and is notably straight (201-1). The state is, however, unknown for *Eoalligator chunyii*. The only other taxon showing this morphology is *Maomingosuchus petrolica*. In *Stangerochampsa mccabei* and *Voay robustus* (Grandidier & Vaillant, 1872), the suture also originates near the dorsal border of the fenestra, but the suture is notably bowed (201-2).

**(202)** Large to very large supraoccipital exposure on skull table is (0) trapezoid, (1) triangular, or (2) block-shaped (new character).

***Comments*:** In most crocodylians, the supraoccipital exposure on the skull table is either small (160-0) or absent (160-1). In some species, however, the exposure is either large (160-2) or very large, excluding the parietal from the posterior edge of skull table (160-3). Among species with a large to very large exposure, the shape can differ markedly. In Orientalosuchina, except for *Jiangxisuchus nankangensis*, which only has a small exposure, the supraoccipital is trapezoid. This also true for *Stangerochampsa mccabei*, *Brachychampsa* spp., *Bottosaurus harlani* and *Globidentosuchus brachyrostris*. In most Caimaninae, on the other hand, the shape is either triangular, for example, in *Paleosuchus* spp. or block-shaped like in *Caiman* spp.. This character is only applicable for taxa with large to very large supraoccipital exposure on the skull roof.

## Supplemental Information

10.7717/peerj.7562/supp-1 Supplemental Information 1 Supplemental File 1.

10.7717/peerj.7562/supp-2 Supplemental Information 2 Supplemental File 2. Character changes.

10.7717/peerj.7562/supp-3 Supplemental Information 3 Supplemental File 3. List of characters.

10.7717/peerj.7562/supp-4 Supplemental Information 4 Supplemental File 4. Postcranial measurements.

## Institutional abbreviations

AMNH: American Museum of Natural History, New York, USA
GMH: Geiseltal Museum of Martin-Luther-University Halle-Wittenberg, Halle (Saale), Germany
GPIT: Geologisch-Paläontologisches Institut Tübingen, Tübingen, Germany
IRSBN: Institut royal des Sciences naturelles de Belgique Brussels, Belgium
IVPP: Institute of Vertebrate Paleontology and Paleoanthropology, Chinese Academy of Sciences, Beijing, China
Kr: Krabi crocodilian, Sirindhorn Museum, Kalasin Province, Thailand
SMNS: Staatliches Museum für Naturkunde Stuttgart, Stuttgart
SZ: Museum der Universität Tübingen, Zoologische Schausammlung, Tübingen, Germany
UMMP: University of Michigan, Museum of Paleontology, Ann Arbor, USA.

## Additional Information and Declarations

### Data Availability

The following information was supplied regarding data availability: The raw data are available in the Supplemental Files.

### New Species Registration

The following information was supplied regarding the registration of a newly described species: Publication LSID: urn:lsid:zoobank.org:pub:08B6F167-AAC7-4184-97BA-B7467D4F036B. *Orientalosuchus* LSID: urn:lsid:zoobank.org:act:BAF6DB3F-0207-42E0-8220-06F9F6D89217. *Orientalosuchus naduongensis* LSID: urn:lsid:zoobank.org:act:9DEB3FC9-ED1F-4D30-96FE-236AD7B82C06.
