# Supplementary material for: A new alligatoroid from the Eocene of Vietnam highlights an extinct Asian clade independent from extant Alligator sinensis
Source: PeerJ. 2019 Nov 5;7:e7562. doi: 10.7717/peerj.7562 (PMC6839522; doi:10.7717/peerj.7562)
Supplement: Supplemental Information 2 — Character changes. [file peerj-07-7562-s002.docx]

# Supplementary 2

**Character changes**

**Character changes for *Navajosuchus mooki* (Simpson, 1930) and *Ceratosuchus burdoshi* Schmidt, 1938**

39

Dorsal midline osteoderms rectangular (0) or nearly square (1).

Brochu (2004) mentioned that these osteoderms are rectangular in *N. mooki* and scored them accordingly as (0), but they were rescored as squared (1) in the matrix of Brochu & Storrs (2012). Based on Brochu (2004, fig.6) and the explicit description of the character we think that the scoring of Brochu & Storrs (2012) might be an error and we rescored *N. mooki* as having rectangular midline osteoderms (0).

50

Dentary gently curved (0), deeply curved (1), or linear (2) between fourth and tenth alveoli.

Basal Alligatorinae were primarily all scored by Brochu (1999) as having a deep curvation, while most *Alligator* spp. have only a gentle curve. It seems, however, the distribution is more complex. Comparing Lucas & Estep (2000, fig.4) for *N. mooki* or Bartels (1984, fig.3) for *C. burdoshi* with recent *Alligator* spp., the curvation is highly comparable (gently curved). This is also true for at least *Procaimanoidea kayi* (Mook, 1941) and in some degree for *Procaimanoidea utahensis* Gilmore, 1946. A deep curvation is indeed present in some basal Alligatorinae *(Hassiacosuchus haupti* Weitzel, 1935, *Arambourgia gaudryi* (de Stefano, 1905), *Allognathosuchus polyodon* (Cope, 1872), *Allognathosuchus wartheni* Case, 1925, but also in *Alligator olseni* White, 1942 and *Alligator mcgrewi* Schmidt, 1941). For those taxa the scoring was not changed. For *N. mooki* and *C. burdoshi* on the other hand it seems the best to score them as gently curved. Therefore, we changed the scoring for both taxa from (1) to (0).

64

Surangular-dentary suture intersects external mandibular fenestra anterior to posterodorsal corner (0) or at posterodorsal corner (1).

*N. mooki* and *C. burdoshi* were scored as (0) in Brochu & Storrs (2012), while in Brochu (2004) they were scored as (1) (*N. mooki*) and (?) (*C. burdoshi*). After evaluating the figures presented by Bartels (1984) and Lucas & Estep (2000), we agree with the scoring of Brochu (2004) and we changed the scoring accordingly.

90

Dorsal premaxillary processes short, not extending beyond third maxillary alveolus (0) or long, extending beyond third maxillary alveolus (1).

*N. mooki* was scored as (0) in Brochu & Storrs (2012) but scored as (1) in Brochu (2004). After evaluating the figures presented by Lucas & Estep (2000) and Brochu (2004, fig.5) we agree with the scoring of Brochu (2004) and we changed the scoring accordingly.

120

Lateral edges of palatines parallel posteriorly (0) or flare posteriorly, producing shelf (1).

The palatines were scored as (0) for *C. burdoshi* in the matrix of Brochu (2004) and Brochu & Storrs (2012) but as producing a shelf (1) in Brochu (1999). The figure 1 of *C. burdoshi* in Bartels (1984) however suggest that the lateral edges indeed flare as scored in Brochu (1999) and we therefore rescored the character as (1).

**Character changes for *Arambourgia gaudryi***

51

Largest dentary alveolus immediately caudal to fourth is (0) 13 or 14, (1) between 11 and 14 and a series behind it, (2) 11 or 12, (3) no differentiation, (4) behind 14, (5) 10. [modified from Brochu & Storrs (2012)]In previous analyses (e.g. Brochu, 2004; Brochu & Storrs, 2012) *A. gaudryi* was scored as (0).

The figures presented in Kälin (1939) and photos kindly provided by Jeremy Martin, however, show that the dentition in the posterior dentary region is not exposed, and we therefore rescored the character as unknown. Individuals from another European taxon with a very similar snout morphology, *Hassiacosuchus haupti* from Messel on the other hand show mostly enlarged bulbous teeth (e.g. HLMD-Me 4415).

79

Teeth and alveoli of maxilla and/or dentary circular in cross-section (0), or posterior teeth laterally compressed (1), or all teeth compressed (2).

This character was scored in Brochu (2004) and Brochu & Storrs (2012) as posteriorly compressed for *A. gaudryi*. As the posterior tooth row is not fully exposed (see also the discussion about character 51), it is more appropriate to rescore as (?). Moreover, as the third before the last dentary tooth of *A. gaudryi* reveals it is more rounded than compressed. The last exposed maxillary tooth in the specimen is most likely the third or fourth before the anatomical last and in *H. haupti*, the bulbous teeth are situated posterior to the corresponding position. We therefore find it more conservative to rescore the character as (?).

**Character changes for *Procaimanoidea kayi* and *Procaimanoidea utahensis***

50

Dentary gently curved (0), deeply curved (1), or linear (2) between fourth and tenth alveoli.

*P. kayi* and *P. utahensis* were previously scored as (1). The shape of the dentary does not differ between *P. kayi* and modern *Alligator* spp. as shown in Mook (1941, fig.1) and we therefore rescored *P. kayi* as (0). In *P. utahensis*, the curvation appears slightly deeper in one dentary but nearly straight in another one (both figured in plate 11 of Gilmore 1946). We therefore rescored the character as (0&1).

51

Largest dentary alveolus immediately caudal to fourth is (0) 13 or 14, (1) between 11 and 14 and a series behind it, (2) 11 or 12, (3) no differentiation, (4) behind 14, (5) 10. [altered after Brochu & Storrs (2012)]

*P. kayi* and *P. utahensis* were scored as (0) in previous studies. Because they have relatively large posterior teeth, we changed the scoring to (1) in this analysis.

54

Splenial participates in mandibular symphysis; splenial symphysis adjacent to no more than five dentary alveoli (0) or splenial excluded from mandibular symphysis; anterior tip of splenial passes ventral to Meckelian groove (1) or splenial excluded from mandibular symphysis; anterior tip of splenial passes dorsal to Meckelian groove (2) or deep splenial symphysis, longer than five dentary alveoli; splenial forms wide ‘V’ within symphysis (3) or deep splenial symphysis, longer than five dentary alveoli; splenial constricted within symphysis and forms narrow ‘V’ (4).

This is scored as (?) for *P. kayi* and as (0) for *P. utahensis* in Brochu (1999), Brochu (2004) and Brochu & Storrs (2012). Based on Mook (1941) the splenial participation for *P. kayi* is indeed unknown but it seems more appropriate to score *P. utahensis* as (?), too. Gilmore (1946:65) stated that “the splenial participates little if at all in the symphysis” and Wassersug & Hecht (1967:32) stated that the “splenial just reaching the symphysis but not actively participating in its formation”. Therefore, for this study, we scored this character as (?) for *Procaimanoidea* *utahensis* as well.

160

Supraoccipital exposure on dorsal skull table small (0), absent (1), large (2), or large such that parietal is excluded from posterior edge of table (3).

In Brochu (1999) and Brochu & Storrs (2012) *P. kayi* is scored as lacking a supraoccipital exposure, whereas it is scored as having an exposure in Brochu (2004). Mook (1941:210) clearly describes its presence: “The supraoccipital occupies a considerable area of the posterior surface of the skull”. Wassersug & Hecht (1967:32) confirm this, they also state the same condition is present in *P.* *utahensis*: “In the cranial table the supraoccipital is visible dorsally in both forms (this character was not noted by Gilmore) as a small triangular insert into the parietal.” Unlike *P. kayi*, *P.* *utahensis* was scored by Brochu & Storrs (2012) as having a supraoccipital exposure on the skull table. The scoring of *P. kayi* was probably an error in Brochu & Storrs (2012) and we here rescored it as (0) following Brochu (2004).

190

Largest premaxillary tooth is the second (0), the third (1) or the fourth (2) or the third and fourth are largest (3) or all similar in size (4) or the fourth and fifth are largest (5) or the first four equal in size (6) (reworded from Wang, Sullivan & Liu, 2016; modified by Li, Wu & Rufolo, 2019)

*P. kayi* was previously scored as having the fourth premaxillary tooth as the largest. The premaxilla, however, is not known for *P. kayi* to our knowledge. We therefore changed the scoring to (?).

**Character changes for *Krabisuchus siamogallicus* Martin & Lauprasert, 2010**

The original scoring of Martin & Lauprasert (2010) was based on the matrix of Brochu (1999). Skutschas et al. (2014) transferred the scorings to the matrix of Brochu & Storrs (2012). While comparing *Orientalosuchus naduongensis* with *K. siamogallicus* we went through the scorings again and found some discrepancies between the original scoring and the transferred one. We further interpreted some characters differently. We listed all changes below.

33

Iliac anterior process prominent (0) or virtually absent (1).

Skutschas et al. (2014) scored this character as (?) while Martin & Lauprasert (2010) scored it as (0). In *K. siamogallicus*, the ilium is preserved and the character can be scored, but scoring the process as prominent seems problematic. In e.g. *Gavialis gangeticus* (Gmelin, 1789), the process is prominent, while the process is nearly absent (with a small visible bump) in e.g. *Alligator mississippiensis* (Daudin, 1802) or *Alligator sinensis* Fauvel, 1879. Although a small process is present in *K. siamogallicus*, the iliac clearly resembles more that of *Alligator* (see also comparison of iliac processes in Brochu (2007, fig.12 (all scored as (1))). In *Orientalosuchus naduongensis*, the process resembles that of *K. siamogallicus*. We therefore rescored the character as (1).

34

Dorsal margin of iliac blade rounded with smooth border (0) or rounded, with modest dorsal indentation (1) or rounded, with strong dorsal indentation (wasp-waisted; 2) or narrow, with dorsal indentation (3) or rounded with smooth border; posterior tip of blade very deep (4).

Skutschas et al. (2014) scored this character as (1) while Martin & Lauprasert (2010) scored it as (2). The iliac blade as interpreted for *K. siamogallicus* is not comparable to other crocodylians and since its preservation is poor, we question whether the blade is actually complete. In *Orientalosuchus naduongensis* the posterior tip of the iliac blade is very deep, while there is a dorsal indentation visible. It is however questionable if the iliac blade of *K. siamogallicus* would potentially look similar if complete. We changed the scoring to (?)until better material is found.

49

Dentary symphysis extends to fourth or fifth alveolus (0) or sixth through eighth alveolus (1) or behind eighth alveolus (2).

Both Martin & Lauprasert (2010) and Skutschas et al. (2014) scored this character as (1)but the states of this character were apparently reversed relative to Brochu (2004). In Brochu (2004, and his subsequent works) state (0) was “sixth or eight alveolus” while state (1) was “fourth or fifth alveolus”. We scored the character as (0) because the dentary symphysis extends to the fifth alveolus.

51

Largest dentary alveolus immediately caudal to fourth is (0) 13 or 14, (1) between 11 and 14 and a series behind it, (2) 11 or 12, (3) no differentiation, (4) behind 14, (5) 10. [modified from Brochu & Storrs (2012)]

Martin & Lauprasert (2010) described the posterior teeth in *K. siamogallicus* as bulbous, but scored them as (0). Although the posterior teeth are smaller compared to other bulbous taxa, the condition in *K. siamogallicus* is as the state (1) described in Brochu (2004) and we therefore score the character accordingly.

61

Anterior processes of surangular unequal (0) or sub-equal to equal (1).

Martin & Lauprasert (2010:619) described the anterior portion as “forked with the medial extension longer and more massive than the lateral extension”. Comparing to Brochu (1999, fig.24), the condition resembles nonetheless more the Alligatoroidea state (1) and that is how we scored here. In some recent examined caimans (*Paleosuchus palpebrosus* (SMNS 11108), *Caiman latirostris* (SZ48b5), *Caiman crocodilus* (SZ 10276)), the character looks very similar to the state of *K. siamogallicus* but they were scored as sub-equal to equal (1). However, there are also some examined recent *Crocodylus* individuals (*C. porosus* (SZ 4120), *C. niloticus* (SZ 381, SZ 9486)), which show a remarkable similarity with the state of these caimans. There seems to be some variation in this character and the scoring could depend on the comparison material. For the moment, we changed the scoring for *K. siamogallicus* to state (1) as in other alligatorids.

67

Surangular continues to dorsal tip of lateral wall of glenoid fossa (0) or truncated and not continuing dorsally (1).

We changed the scoring in *K. siamogallicus* from (0) to (?). We find the preservation insufficient for a reliable scoring and Martin & Lauprasert (2010) did not explicitly mention it in the description.

79

Teeth and alveoli of maxilla and/or dentary circular in cross-section (0), or posterior teeth laterally compressed (1), or all teeth compressed (2).

Martin & Lauprasert (2010) scored this character as (0), but they stated: “Kr-C-066 preserves two crushing posterior teeth, which are low crowned with rounded surfaces. One of these teeth is particularly longer than wide (Fig. 7E)” (Martin & Lauprasert 2010:619). This raises the question if the posterior teeth are similar to that of *Orientalosuchus naduongensis* where the posterior teeth are laterally compressed. There is actually no fig. 7E in Martin & Lauprasert (2010) to verify the morphology. We scored (1) following the description.

90

Dorsal premaxillary processes short, not extending beyond third maxillary alveolus (0) or long, extending beyond third maxillary alveolus (1).

Martin & Lauprasert (2010) scored this as (0), but in their description mentioned “...differs from all other alligatorines in having a long dorsal premaxillary process...” and “...the posterior premaxillary process is long and extends at least to the level of the third maxillary alveolus”. Thus, in the present study, we scored this character as a long premaxillary process (1).

92

All dentary teeth occlude lingual to maxillary teeth (0) or occlusion pit between seventh and eighth maxillary teeth; all other dentary teeth occlude lingually (1) or dentary teeth occlude in line with maxillary toothrow (2).

We rescored *K. siamogallicus* from (0) to (?). The tooth row is not well preserved and the character is not explicitly described but the outline of the maxilla resembles *O. naduongensis*.

93

Largest maxillary alveolus is 3 (0), 5 (1), 4 (2), 4 and 5 are same size (3), 6 (4), or maxillary teeth homodont (5), or maxillary alveoli gradually increase in diameter posteriorly toward penultimate alveolus (6).

*K. siamogallicus* was previously scored as (2). Martin & Lauprasert (2010) stated: “The best count is obtained on Kr-C-021, a right maxilla with 14 alveoli. The fourth alveolus is the largest.” but also: “Kr-C-021: a cervical vertebra associated with isolated teeth”. In fact, no maxilla with this number is figured and none of the other specimens verify this claim. Moreover, in *O. naduongensis*, a close relative of *K. siamogallicus*, the fifth is the largest. We conservatively scored (?) for the Thai taxon.

97

Preorbital ridges absent or very modest (0) or very prominent (1) at maturity.

*K. siamogallicus* was previously scored as (0) by Martin & Lauprasert (2010). Martin & Lauprasert (2010) does not describe the character and the skulls are poorly preserved. In *O. naduongensis*, the ridges border the nasals and reach the premaxilla. In *K. siamogallicus,* the alleged lacrimal reaches the premaxilla and excludes the maxilla from reaching the nasal bones which would be a very unusual condition. If the lacrimals would be interpreted as ridges, it is more likely that much of the lacrimal-nasal suture figured by Martin & Lauprasert (2010) (fig.2,3) corresponds to the preorbital ridge seen in *O. naduongensis*. Since we only had access to photographs of *K. siamogallicus*, we rather score the character as unknown (?)for the time being.

104

Ectopterygoid abuts maxillary tooth row (0) or maxilla broadly separates ectopterygoid from maxillary tooth row (1).

Skutschas et al. (2014) scored this character as (?), while Martin & Lauprasert (2010) scored it as (1). Martin & Lauprasert (2010:613) stated that “the ectopterygoid does not touch the posterior maxillary tooth row but it is not excluded by a wide extension of the maxilla”. Based on this, we score the character as (1).

112

Maxilla has linear medial margin adjacent to suborbital fenestra (0) or bears broad shelf extending into fenestra, making lateral margin concave (1).

This character was rephrased from the original character scored by Martin & Lauprasert (2010): “Lateral edge of suborbital fenestra straight (0) or bowed medially (1)”. The anterolateral portion of the suborbital fenestra seems not well preserved as it is not visible in any of the figures and we therefore scored the character as (?) in this analysis.

115

Palatine process extends (0) or does not extend (1) significantly beyond anterior end of suborbital fenestra.

Instead of (0) as in Martin & Lauprasert (2010) we scored (?). The shape of the suborbital fenestra, especially in the anterior region is unclear and the possibility that it extends further anterior cannot be excluded.

119

Pterygoid ramus of ectopterygoid straight, posterolateral margin of suborbital fenestra linear (0) or ramus bowed, posterolateral margin of fenestra concave (1).

This character was rewordedin Brochu & Storrs (2012) from Brochu (1999) (character 88). Former phrasing: “Suborbital fenestra without (0) or with (1) posterior notch”. Martin & Lauprasert (2010) scored this as (0) but state that “The lateral edge of the suborbital fenestra is made up of the medially bowed ectopterygoid“. This indicates more character state (1) where the ectopterygoid is bowed and we rescored the character as such.

120

Lateral edges of palatines parallel posteriorly (0) or flare posteriorly, producing shelf (1).

Martin & Lauprasert (2010) scored *K. siamogallicus* as (1), but they figure parallel edges, with, if any, a minimal posterior flaring, especially if compared with recent *Alligator* spp. or *Hassiacosuchus haupti* (ibid. fig.4). We therefore rescored this character as (0). Such a minimal flaring is also present in *Diplocynodon* spp., which further share an anterior positioned palatine-pterygoid suture with *O. naduongensis* and *K. siamogallicus*. We also revised this character for *Diplocynodon* spp., as the condition resembles more the *Crocodylus* condition than the *Alligator* spp. condition.

125

Internal choana not septate (0) or with septum that remains recessed within choana (1) or with septum that projects out of choana (2).

The choana was previously scored as lacking a septum but in Martin & Lauprasert (2010, fig.4), there is an anteroposteriorly extending bone lamina visible, which well could be the septum. The choana was only briefly described without mentioning the lamina and thus it is more conservative to score as (?).

128

Lacrimal makes broad contact with nasal; no posterior process of maxilla (0) or maxilla with posterior process within lacrimal (1) or maxilla with posterior process between lacrimal and prefrontal (2).

We changed the scoring for this character from (0) to (?). The general interpretation of the lacrimal is questionable (see 97).

132

Ectopterygoid extends along medial face of postorbital bar (0) or stops abruptly ventral to postorbital bar (1).

This character was previously scored as (0), but Martin & Lauprasert (2010:617) stated that “the ectopterygoid participation on the postorbital bar is unknown”. We therefore scored this character as (?).

135

Ventral margin of postorbital bar flush with lateral jugal surface (0) or inset from lateral jugal surface (1).

Martin & Lauprasert (2010) scored this as (0). As in character 90, there is a discrepancy between the scoring and the description. Martin & Lauprasert (2010:615) mentioned: “The postorbital bar is inset from the dorsal margin of the jugal.” We follow the description and score as (1) in this study.

177

Quadrate foramen aerum on mediodorsal angle (0) or on dorsal surface (1) of quadrate.

Martin & Lauprasert (2010) scored this as (1). However Martin & Lauprasert (2010:615) mentioned: “The foramen aerum is visible close to the medial margin on the left quadrate” and (2010:623): “Finally the fact that the quadrate foramen aerum does not face dorsally is unusual for Alligatoroidea”. This indicates that the scoring in the matrix is probably incorrect and we scored this character as (0) in this study.

178

Quadrate foramen aereum is small (0), comparatively large (1), or absent (2) at maturity.

According to Brochu (2011) this character comes from Brochu (2004) but the cited character pertains the form of the posterior maxillary alveoli and has only two character states instead of three. It is, therefore, likely that Martin & Lauprasert (2010) did not score the size of the quadrate foramen aerum. In their description, there is no clue provided for scoring this character. It is most likely a small foramen as outlined in their fig. 2 but without studying the original material, we scored the character as (?) in this study.

196

Notch between the premaxilla and maxilla present (0) or not present (1) in adult individuals.

We scored the new character 195 of this study as (?) in this analysis. Martin & Lauprasert (2010) mentioned in some of the individuals a notch between the premaxilla and maxilla but did not observe any indication of a notch in others, making the scoring problematic

**Character changes for *Protoalligator huiningensis* Young, 1982**

51

Largest dentary alveolus immediately caudal to fourth is (0) 13 or 14, (1) between 11 and 14 and a series behind it, (2) 11 or 12, (3) no differentiation, (4) behind 14, (5) 10. [modified from Brochu & Storrs (2012)]

Wang, Sullivan & Liu (2016) scored this character as (2). While the visible posterior teeth look smaller than the large 11th tooth, the posterior most teeth after the 13th one are missing. A fragmentary tooth is figured in Wang, Sullivan & Liu (2016, fig.10A). This presumably 14th tooth looks slightly larger than the 13th one, which could mean that state (1) would be more accurate. However, the preservation is poor, which makes us scoring as (?) in this study.

52

Splenial with anterior perforation for mandibular ramus of cranial nerve V (0) or lacks anterior perforation for mandibular ramus of cranial nerve V (1).

The splenial is fragmentary with a damaged surface. Whether the anterior perforation for the mandibular ramus of the fifth cranial nerve is absent or simply not preserved is difficult to establish. Therefore, it seems more appropriate to score this character as (?) until better material is available.

63

External mandibular fenestra absent (0) or present as narrow slit, no discrete fenestral concavity on angular dorsal margin (1) or present with discrete concavity on angular dorsal margin (2) or present and very large; most of foramen intermandibularis caudalis visible in lateral view (3).

Wang, Sullivan & Liu (2016) previously scored this character as (1&2) for *P. huiningensis*, but based on their provided figures, the fenestra could not have had the form of a narrow slit in the living animal and it was not as large as in modern *Alligator*. We therefore score the character as (2).

82

External naris bisected by nasals (0) or nasals contact external naris, but do not bisect it (1) or nasals excluded, at least externally, from naris; nasals and premaxillae still in contact (2) or nasals and premaxillae not in contact (3).

A bisected naris, as mentioned in Wang, Sullivan & Liu (2016), would be unexpected to find in an Alligatoroid outside of *Alligator* spp. but the evidence for this is not very strong. The material itself does not show a bisection, only an anterior and posterior process projecting into the naris. It is possible that they were connected but it is unclear. Wang, Sullivan & Liu (2016) explained their decision of scoring the naris as bisected because of a posteriorly reaching premaxilla process, which is visible at the anterior part of the naris. Such a process is also present in *Alligator* spp. and forms the anterior part of the bisection.

In *Orientalosuchus naduongensis*, such a posterior reaching process is also preserved but, although better preserved, a complete bisection cannot be found. We therefore score this character conservatively as (?) until better preserved material of the specimen is present. There remains the possibility that a cartilage bisection was present in both species, which began to ossify and represents a precursor of the *Alligator* spp. state and an intermediate scoring coupled with ordering could be a solution for future studies.

97

Preorbital ridges absent or very modest (0) or very prominent (1) at maturity.

The preorbital ridges in *P. huiningensis* were not previously scored because the prefrontal-lacrimal region is not preserved. While that may be true, the state looks clearly different from *O. naduongensis*. The preorbital ridges in the latter are very dominant and projecting towards the premaxilla, which is clearly not the case in *P. huiningensis*. Therefore, we changed the scoring from (?) to (0).

131

Anterior tip of frontal (0) forms simple acute point or (1) forms broad, complex sutural contact with the nasals.

The anterior tip of the frontal is not clearly visible in *P. huiningensis*. It was previously scored as a broad suture but Wang, Sullivan & Liu (2016) are uncertain if the present structure is a suture or not. Therefore, we score the character as (?) in this analysis.

190

Largest premaxillary tooth is the second (0), the third (1) or the fourth (2) or the third and fourth are largest (3) or all similar in size (4) or the fourth and fifth are largest (5) or the first four equal in size (6) (reworded from Wang, Sullivan & Liu, 2016; modified by Li, Wu & Rufolo, 2019)

This character was introduced by Wang, Sullivan & Liu (2016) and altered by Li, Wu & Rufolo, 2019. *P. huiningensis* was scored with the same condition as most alligatorids in having the fourth premaxillary tooth the largest. However, the total tooth count in the premaxilla is different from other alligatoroids, as only four teeth in total seem to be present. Usually the last tooth is the fifth one because of an omission in more anterior teeth. A gradually enlarging of the teeth, as described, would therefore result in the originally fifth one as the largest one. The problem in *P. huiningensis* is that all premaxillary teeth seem very similar in size and we therefore score the character conservatively as (?).

196

Notch between the premaxilla and maxilla present (0) or not present (1) in adult individuals.

Wang, Sullivan & Liu (2016) scored character 91 as if no notch were present in juvenile states, which seems very strange, taking into account that the only preserved individual is an adult, which shows a very prominent notch between the premaxilla and maxilla.

Thus, in the new formed character 195, we score *P. huiningensis* as having a notch (0) and character 91 as (?) because it is unknown how it looked in juvenile individuals.

**Character changes for the Maoming alligatoroid**

97

Preorbital ridges absent or very modest (0) or very prominent (1) at maturity.

Skutschas et al. (2014) scored the preorbital ridges as absent or very modest (0). However, their figures and description from clearly show the presence of very large preorbital ridges highly similar to the ones of *Orientalosuchus naduongensis*. We therefore score the character as (1).

131

Anterior tip of frontal (0) forms simple acute point or (1) forms broad, complex sutural contact with the nasals.

Skutschas et al. (2014) described the anterior tip of the frontal as broad. The anterior frontal region is, however, poorly preserved. Comparing the structure with *O. naduongensis*, there is a high possibility that the sutural contact between the nasal and frontal is more pointed than broad. We score the character as (?), until better-preserved material becomes available.

151

Frontoparietal suture concavoconvex (0) or linear (1) between supratemporal fenestrae.

We change the scoring for this character from (1) to (0&1). The suture looks very similar to *O. naduongensis* being only slightly-wavy and is therefore best scored intermediate between the two states..

196

Notch between the premaxilla and maxilla present (0) or not present (1) in adult individuals.

This character is problematic to score. While it seems tempting to score the Maoming alligatoroid as lacking a notch between the premaxilla and maxilla, the preservation is insufficient. GPIT/RE/09728 (Fig. 5) shows that a notch can easily vanish if the preservation is suboptimal. We score the character as (?)until better material is available.

**Character changes for *Eoalligator chunyii* Young, 1964**

1

Ventral tubercle of proatlas more than one-half (0) or no more than one-half (1) the width of the dorsal crest.

This character was scored by Wang, Sullivan & Liu (2016) as (0), but they mentioned: “A small ventral tuberosity representing the point of attachment of the atlantoccipital ligament...”. The proatlas resembles clearly the appearance in recent *Alligator* and the tuberosity indeed seems more narrow than broad. Therefore, we alter the scorings to (1) in this analysis.

13

Posterior half of axis neural spine wide (0) or narrow (1).

Wang, Sullivan & Liu (2016) scored this character as narrow (1). In the only preserved axis neural spine the dorsal part is broken off making a reliable scoring of this character impossible, which is also mentioned in Wang, Sullivan & Liu (2016:19): “...although only the base of the neural spine is present.” We therefore score the character as (?) in this analysis.

15

Axial hypapophysis located toward the center of centrum (0) or toward the anterior end of centrum (1).

Wang, Sullivan & Liu (2016) scored this character as (0), which would be the common state in Globidonta, but comparing the provided figures of Wang, Sullivan & Liu (2016) with Brochu (1999, fig.30), the hypapophysis in *E. chunyii* is posteriorly shifted as in *Diplocynodon* and *Orientalosuchus naduongensis*. We therefore change the scoring to (0) in this analysis.

38

Dorsal osteoderms not keeled (0) or keeled (1).

This character was previously not scored in *E. chunyii*, but Wang, Sullivan & Liu (2016, fig.7) reveals the presence of such a keel in the osteoderms. We therefore change the scoring from (?) to (1).

50

Dentary gently curved (0), deeply curved (1), or linear (2) between fourth and tenth alveoli.

*E. chunyii* was previously scored as having a gently curved dentary (0). Based on the material presented by Wang, Sullivan & Liu (2016), this seems questionable. The only preserved dentary seems laterally twisted and the fourth dentary alveolus is incomplete. We therefore score the character as (?).

51

Largest dentary alveolus immediately caudal to fourth is (0) 13 or 14, (1) between 11 and 14 and a series behind it, (2) 11 or 12, (3) no differentiation, (4) behind 14, (5) 10. [modified from Brochu & Storrs (2012)]

Wang, Sullivan & Liu (2016) scored this character as (2). The posterior dentary teeth or alveoli are not clearly visible, but in Wang, Sullivan & Liu (2016, fig. 6A) they do not seem much smaller than the enlarged 11th dentary tooth. Wang, Sullivan & Liu (2016, fig. 2D), on the other hand, shows posterior maxillary teeth which are not small and round at all but broad and elongated like the teeth in e.g. *O. naduongensis* and *Alligator prenasalis* (Loomis, 1904). It seems unlikely that the posterior maxillary teeth are that enlarged while the posterior dentary teeth were small and round. It therefore seems better to score them as enlarged as well and we change the state to (1) in this analysis.

54

Splenial participates in mandibular symphysis; splenial symphysis adjacent to no more than five dentary alveoli (0) or splenial excluded from mandibular symphysis; anterior tip of splenial passes ventral to Meckelian groove (1) or splenial excluded from mandibular symphysis; anterior tip of splenial passes dorsal to Meckelian groove (2) or deep splenial symphysis, longer than five dentary alveoli; splenial forms wide ‘V’ within symphysis (3) or deep splenial symphysis, longer than five dentary alveoli; splenial constricted within symphysis and forms narrow ‘V’ (4).

This character was previously scored as (2) in *E. chunyyi*. While the splenial is excluded from the mandibular symphysis, it is not that clear if the anterior tip passes ventral or dorsal to the Meckelian groove based on the figures in Wang, Sullivan & Liu (2016). This is also not mentioned in the description. In their fig. 6, the imprint of the splenial looks like if the anterior tip would lie more centrally over the Meckelian groove, but without seeing the original material it seems premature to score. We therefore score the character as (1&2) in this analysis.

67

Surangular continues to dorsal tip of lateral wall of glenoid fossa (0) or truncated and not continuing dorsally (1).

We change the scoring for this character from (0) to (1). Although Wang, Sullivan & Liu (2016) describe a surangular reaching the dorsal tip of the lateral wall of the glenoid fossa, they only refer this to IVPP V 2716-3 while in IVPP V 2721.1 (both fig. 6) the condition is truncated. In IVPP V 2716-3, the surangular-articular region is not as good preserved, leaving a potential dorsal projecting surangular in this case as a deformation result.

79

Teeth and alveoli of maxilla and/or dentary circular in cross-section (0), or posterior teeth laterally compressed (1), or all teeth compressed (2).

This was previously scored as (?) by Wang, Sullivan & Liu (2016), but their fig.2 shows a laterally compressed posterior tooth and we therefore change the scoring to (1).

102

Medial jugal foramen small (0) or very large (1).

Wang, Sullivan & Liu (2016) scored the medial jugal foramen as very large (1), which is unusual for alligatorids and is only present in some *Diplocynodon* taxa. They do not mentione it in their description and based on the provided figures such a foramen is not preserved. We therefore score the character as (?) in this analysis.

Palatine-characters

Wang, Sullivan & Liu (2016, fig.3) shows the dorsal and ventral view from the snout of *E. chunyii*, but it seems that a potential mistake in the interpretation of the anterior and posterior part was made. In *Orientalosuchus naduongensis*, the anterior palatine process is very short, not projecting anteriorly to the large suborbital fenestrae. In crocodylians, this condition is rare and only present in few taxa, e.g. *Osteolaemus tetraspis* Cope, 1861. The presence of such an unusual condition could have misled Wang, Sullivan & Liu (2016) in their interpretation. What they interpreted as the pterygoid could rather be the maxilla, which gets further support by the presence of two alveoli on the right lateral side. The tooth row therefore does not proceed posterior to the external mandibular fenestra contrary to Wang, Sullivan & Liu (2016).

This also raises a different interpretation in dorsal view. The lacrimal on the left side could be the premaxilla with a clear visible premaxilla-maxilla suture and the prefrontal and right lacrimal could be interpreted as the deformed external narial opening in which the nasal projects anteriorly. The frontal could be interpreted as relatively narrow, similar to that of *Orientalosuchus naduongensis* and the lacrimal longer than the prefrontal. The anterior opening could be the orbita. However, without seeing the original material, all of the dorsal sutures is more or less a guess, so we scored related characters of the dorsal view unknown (this involves all dorsal snout characters from 81-98 and 128-131 in the character list of Brochu & Storrs, 2012).

The ventral region is better preserved, allowing for rescoring characters:

(115) Palatine process extends (0) or does not extend (1) significantly beyond anterior end of suborbital fenestra. We score this character as (1).

(118) Palatine-pterygoid suture nearly at (0) or far from (1) posterior angle of suborbital fenestra, which was previously scored by Wang, Sullivan & Liu (2016). We score the character as (?), as the suture, after reinterpretation, is not present anymore. (119) Pterygoid ramus of ectopterygoid straight, posterolateral margin of suborbital fenestra linear (0) or ramus bowed, posterolateral margin of fenestra concave (1). Wang, Sullivan & Liu (2016) scored (1) and we score the character as (?), after reinterpretation.

140

Quadratojugal spine low, near posterior angle of infratemporal fenestra (0) or high, between posterior and superior angles of infratemporal fenestra (1).

Wang, Sullivan & Liu (2016) scored the quadratojugal spine as present and argued that the small dorsal process visible in their fig. 6A shows the spine, but contrary to their interpretation, this rather seems to be the posterior margin of the infratemporal fenestra and the presence of a potential spine cannot be identified and we therefore change the scoring to (?) in this analysis.

142

Quadratojugal forms posterior angle of infratemporal fenestra (0) or jugal forms posterior angle of infratemporal fenestra (1) or quadratojugal-jugal suture lies at posterior angle of infratemporal fenestra (2).

Wang, Sullivan & Liu (2016) scored this character as (?) but it can be seen in their fig. 6A, that the quadratojugal forms the posterior angle of the infratemporal fenestra. We therefore score this character as (0) in this analysis.

148

Quadrate and squamosal not in contact on the external surface of the skull, posteriorly to the external auditory meatus (0) or quadratosquamosal suture extends dorsally along caudal margin of the external auditory meatus (1) or extends only to the caudoventral corner of the external auditory meatus (2). (Modified by Delfino, Martin & Buffetaut (2008) from Brochu (1999), and Salisbury et al. (2006), character 132).

Wang, Sullivan & Liu (2016) scored this character as (0). This condition is not known for a single Brevirostres species*.* Based on their fig. 5A, it seems ambiguous to score this character because of the bad preservation in this area. If the suture interpretation by Wang, Sullivan & Liu (2016) is correct, the quadrate and squamosal are in contact and allows scoring this character as (1), a condition that is still absent in Globidonta but is present in some *Diplocynodon* species.

149

Caudal margin of otic aperture not defined and gradually merging into the exoccipital (0) or smooth and continuous with the paraoccipital process (1) or caudal margin of otic aperture inset (2). (Modified by Delfino, Martin & Buffetaut (2008) from Salisbury et al. (2006), character 102).

Wang, Sullivan & Liu (2016) scored this as (1), but described that the posterior margin of the otic recess forms a small posterior notch. We therefore score the character as (2) in this analysis.

160

Supraoccipital exposure on dorsal skull table small (0), absent (1), large (2), or large such that parietal is excluded from posterior edge of table (3).

Wang, Sullivan & Liu (2016) scored this character for *E. chunyii* as small (0), but their Fig. 4, shows a very prominent supraoccipital exposure, which is much larger than the exposure e.g. in recent *Crocodylus* taxa and resembles that of *Paleosuchus palpebrosus* (Cuvier, 1807). We therefore score this character as (2) in this analysis.

196

Notch between the premaxilla and maxilla present (0) or not present (1) in adult individuals.

Wang, Sullivan & Liu (2016) described an occlusal pit between the premaxilla and maxilla, which can be seen in their fig. 2A. Because of preservation, we find this interpretation however questionable and we therefore score this character as (?).

Nonetheless, if the interpretation from Wang, Sullivan & Liu (2016) is correct than *E. chunyii* differs from *O. naduongensis*, which has a clear notch in adults. Because of its size, it seems unlikely that *E. chunyii* is a juvenile, leaving a potential ontogenetic changeas in *Caiman crocodilus* (Linnaeus, 1758) out of question.

**Character changes for *Jiangxisuchus nankangensis* Li, Wu & Rufolo, 2019**

51

Largest dentary alveolus immediately caudal to fourth is (0) 13 or 14, (1) between 11 and 14 and a series behind it, (2) 11 or 12, (3) no differentiation, (4) behind 14, (5) 10. [modified from Brochu & Storrs (2012)]

This character was previously scored as (2). The largest dentary alveolus immediately caudal to the fourth is indeed the 12^th^. It is, however, unknown, if the dentary teeth are enlarged like in *Orientalosuchus naduongensis*, or not. The dentary alveoli of the 13^th^ and 14^th^ tooth are smaller than the 12^th^. However, this is also the case in species with enlarged posterior teeth. Caudal to the 11 ^th^ to 14 ^th^-tooth, there are two smaller ones, before the large bulbous or large compressed teeth. In *J. nankangensis*, the posterior alveoli seem relatively large and elongated. This would also be consistent with the elongated 11^th^ maxillary tooth. We therefore changed the scoring to (?), because with the current material it is impossible to determine if the character should be scored (2) or (1).

52

Splenial with anterior perforation for mandibular ramus of cranial nerve V (0) or lacks anterior perforation for mandibular ramus of cranial nerve V (1).

This character was previously scored as (0), but the opening for the cranial nerve V rather seems to slip out anteriorly from the splenial, instead of going through a foramen on the anterior end of the splenial. The opening for the cranial nerve is not entirely on the splenial but only the posterior border of the exit is formed by this element. An intermediate state seems therefore the most conservative way to score this character in *J. nankangensis* and we changed the scoring to (0&1). This character may be more variable than expected and the position of the cranial nerve exit can be asymmetric (e.g. *Alligator sinensis* SMNS 4915).

54

Splenial participates in mandibular symphysis; splenial symphysis adjacent to no more than five dentary alveoli (0) or splenial excluded from mandibular symphysis; anterior tip of splenial passes ventral to Meckelian groove (1) or splenial excluded from mandibular symphysis; anterior tip of splenial passes dorsal to Meckelian groove (2) or deep splenial symphysis, longer than five dentary alveoli; splenial forms wide ‘V’ within symphysis (3) or deep splenial symphysis, longer than five dentary alveoli; splenial constricted within symphysis and forms narrow ‘V’ (4).

This character was previously scored as (2). The anterior tip of the splenial, however, seems to pass rather ventral (1) than dorsal (2) to the Meckelian groove based on the figures of Li, Wu & Rufolo (2019). The anterior process is broken off and displaced slightly dorsally, but its original position is seen in form of an imprint, which lies ventrally to the groove. We therefore changed the scoring to (1).

61

Anterior processes of surangular unequal (0) or sub-equal to equal (1).

This character was previously scored as (0). Usually, in members of Crocodyloidea, there is none or only a rather small ventral process visible, whereas the process can prominently be seen in members of Alligatoroidea. In *J. nankangensis*, the ventral process is truncated but clearly visible, yet it was scored with (0), while in case of *Navajosuchus mooki*, the ventral process is clearly shorter than the dorsal, yet it was scored with (1). The ventral process in *J. nankangensis* is best scored intermediate (0&1). In the future, a quantification of this character could be an appropriate solution to prevent subjective interpretations of what is in fact continuous morphological variation.

79

Teeth and alveoli of maxilla and/or dentary circular in cross-section (0), or posterior teeth laterally compressed (1), or all teeth compressed (2).

This character was previously scored as (0), but the 11^th^ maxillary tooth of *J. nankangensis* is in fact laterally compressed. Moreover, the elongated posterior alveoli suggest elongated teeth. Li, Wu & Rufolo (2019:31) stated: “Starting from the tenth tooth (on the right maxilla), the bases of the maxillary teeth become constricted and the crown becomes low and bean-shaped”. This condition is very similar to the one in *O. naduongensis* We therefore changed the scoring to (1).

86

Premaxillary surface lateral to naris smooth (0) or with deep notch lateral to naris (1).

This character was previously scored as (1), but a notch lateral to the naris opening is neither mentioned nor figured for *J. nankangensis*, instead, Li, Wu & Rufolo (2019:27) stated: “The dorsal surface of the premaxilla is slightly elevated along the lateral margin of the naris.” This is the case in many Eusuchians. We therefore changed the scoring to (0), as a notch like in *Alligator* spp. is not visible in *J. nankangensis*.

104

Ectopterygoid abuts maxillary tooth row (0) or maxilla broadly separates ectopterygoid from maxillary tooth row (1).

This character was previously scored as (0), but the ectopterygoid of *J. nankangensis* does not contact the maxillary tooth row and we score (1). There is a posteriorly projecting process of the maxilla visible, which prevents the contact between the ectopterygoid and the tooth row up to the posteriormost tooth. The ectopterygoid is not strongly bowed, a condition similar to *O. naduongensis*.

167

Parietal with recess communicating with pneumatic system (0) or solid, without recess (1).

This character was previously scored as (0). The character itself, however, does seem to be problematic. It was not scored for any fossil taxa in the matrix of Brochu & Storrs (2012) but scored for some of them in the matrix of Brochu (1999). Furthermore, the scoring for the recent taxa was reversed. In Brochu (1999) alligatoroidea were scored as (0) and Crocodyloidea scored as (1). In Brochu & Storrs (2012) it was the other way around. As long as only recent taxa are scored for this character and the previously difference between Crocodyloidea and Alligatoroidea is maintained, it does not affect the tree, but scoring *J. nankangensis* could lead to a possible wrong correlation with either one of the two groups. We therefore changed the scoring to (?) for the time being.

181

Quadrate with small, ventrally-reflected medial hemicondyle (0) or with small medial hemicondyle; dorsal notch for foramen aerum (1) or with prominent dorsal projection between hemicondyles (2) or with expanded medial hemicondyle (3).

This character was previously scored as (0). The medial hemicondyle, however, do not seem ventrally reflected but more on the same horizontal line as the lateral hemicondyle. The medial hemicondyle is also smaller than the lateral one. A small dorsal notch is visible on the entrance of the foramen (Li, Wu & Rufolo 2019, fig.5) as is also the case for *O. naduongensis*. We therefore changed the scoring to (1).

**Character changes for *Bottosaurus harlani* Meyer, 1832**

51

Largest dentary alveolus immediately caudal to fourth is (0) 13 or 14, (1) between 11 and 14 and a series behind it, (2) 11 or 12, (3) no differentiation, (4) behind 14, (5) 10. [altered after Brochu & Storrs (2012)]

Due to the enlarged but elongated posterior dentary teeth, we altered the scoring from (2) to (1).

67

Surangular continues to dorsal tip of lateral wall of glenoid fossa (0) or truncated and not continuing dorsally (1).

Based on Cossette & Brochu (2018, fig.6) it seems very unlikely that the surangular continues to the dorsal tip of lateral wall of glenoid fossa and we changed the scoring to (1).

The following characters were not scored for *B. harlani* due to the different matrix used in Cossette & Brochu (2018): 118 - 1; 131 - 0; 135 - 0 and 181-200.

**Character changes for other taxa**

34

Dorsal margin of iliac blade rounded with smooth border (0) or rounded, with modest dorsal indentation (1) or rounded, with strong dorsal indentation (wasp-waisted; 2) or narrow, with dorsal indentation (3) or rounded with smooth border; posterior tip of blade very deep (4).

We changed this character for *Stangerochampsa mccabei* from (0) to (?). The figure of Wu, Brinkman & Russell (1996, plate 3) suggests that the dorsal order of the ilium is damaged in the region of a possible indentation. It is therefore impossible to tell if the dorsal margin was smooth or had an indentation.

38

Dorsal osteoderms not keeled (0) or keeled (1).

We changed the scoring of *S. mccabei* from (0) to (0&1). The dorsal osteoderms of *S. mccabei*, *Gavialis gangeticus* and *Orientalosuchus naduongensis* show an intermediate state. In all three, a shallow keel is visible but it is much lower than in most other crocodilians.

67

Surangular continues to dorsal tip of lateral wall of glenoid fossa (0) or truncated and not continuing dorsally (1).

We changed this character in *Alligator sinensis* from (0) to (1). Brochu (1999) states that *A. sinensis*, unlike *Alligator mississippiensis*, does not have a truncated surangular. However, in all specimens of *A. sinensis* available to our study (IRSNB 13904-3487, SMNS 4915, ZNS 54/969) the condition is more similar to A*. mississippiensis* than to e.g. *Caiman latirostris* (Daudin, 1801) in which the surangular reaches all the way up to the tip.

79

Teeth and alveoli of maxilla and/or dentary circular in cross-section (0), or posterior teeth laterally compressed (1), or all teeth compressed (2).

We changed the scoring for *Alligator prenasalis*, *Brachychampsa montana* Gilmore, 1911 and *Brachychampsa sealey* Williamson, 1996. In *A. prenasalis* and *Brachychampsa montana* we changed the scoring from (0) to (1) and in *Brachychampsa sealey* from (0) to (?). In Cossette & Brochu (2018), *Bottosaurus harlani* was described with large but elongated teeth, which is very similar to *O. naduongensis*. Cossette & Brochu (2018) further mentioned “Teeth resembling those of *B. harlani* are found in species of the fossil alligatoroid *Brachychampsa*.“ While this is true for *Brachychampsa montana*, the posterior maxilla teeth of *Brachychampsa sealeyi* are much more rounded and only its dentary teeth are more elongated. Therefore, we rescored *Brachychampsa montana* as elongated (2) and *Brachychampsa sealeyi* as (?). In *A. prenasalis*, the lower jaw of YPM VPPU 014063 shows that the enlarged posterior teeth are also elongated and we changed the scoring accordingly to (2).

82

External naris bisected by nasals (0) or nasals contact external naris, but do not bisect it (1) or nasals excluded, at least externally, from naris; nasals and premaxillae still in contact (2) or nasals and premaxillae not in contact (3).

We changed the scoring *Protoalligator huiningensis* (see above) and in *A. prenasalis* from (0) to (?). The nasal bone in the latter reaches far anterior into the naris, but it is unclear if the naris was really bisected or not. A posteriorly reaching premaxilla process, present in other *Alligator* species as well as in *P. huiningensis* and *O. naduongensis* is also missing or not preserved. We therefore decided for a conservative rescoring to (?) for this character.

120

Lateral edges of palatines parallel posteriorly (0) or flare posteriorly, producing shelf (1).

This character was scored for *Diplocynodon* spp. as having posteriorly flared palatines, but the condition resembles more that in *Crocodylus* or *Leydiosuchus* *canadensis* Lambe, 1907 (0) than *Alligator* spp. (1). A weak flaring of the suborbital bridge occurs in *Diplocynodon* spp. at the posterior corner of the suborbital fenestra but is formed by the pterygoid unlike as in e.g. *Alligator* spp. in which it is formed by the palatine. For the sake of objectivity, we rescored this character as (0).

123

Pterygoid surface lateral and anterior to internal choana flush with choanal margin (0) or pushed inward anterolateral to choanal aperture (1) or pushed inward around choana to form neck surrounding aperture (2) or everted from flat surface to form neck surrounding aperture (3).

Old character Brochu (1999): Pterygoid surface lateral and anterior to internal choana flush with choanal margin (0) or pushed inward to form "neck" (1).

The character of Brochu (1999) was reworded by Brochu (2011) and the former (1) was divided into three different character states. *Osteolaemus* spp., *Voay robustus* (Grandidier & Vaillant, 1872), *Boverisuchus magnifrons* Kuhn, 1938, *Brochuchus pigotti* (Tchernov & Van Couvering, 1978) and *Alligator olseni* were scored as (1), but picture comparison revealed that it is more appropriate to score *Osteolaemus* spp. and *V. robustus* as (2), the same condition as *O. naduongensis*. For *Boverisuchus magnifrons*, we could not check the scoring (1) and we therefore decided to score it as (?). *Brochuchus pigotti* was scored as (1) and Conrad et al. (2013) mentioned that there was no choanal neck visible, so we retained the previous scoring (1). *A. olseni* was also scored as (1) but its choanal region is in poor shape making a scoring based on pictures problematic and we therefore rescored it as (?).

190

Largest premaxillary tooth is the second (0), the third (1) or the fourth (2) or the third and fourth are largest (3) or all similar in size (4) or the fourth and fifth are largest (5) or the first four equal in size (6) (reworded from Wang, Sullivan & Liu, 2016; modified by Li, Wu & Rufolo, 2019)

The scoring for species with only four teeth early in ontogeny is problematic. *Paleosuchus* spp., *Osteolaemus* spp., *Piscogavialis jugaliperforatus* and *Allodaposuchus subjuniperus* are scored as having only four teeth early in posthatching ontogeny (87-1). This has a direct effect for the scoring of (190), because it is unclear, which premaxillary tooth was lost in those species from the ancestral five teeth condition. In e.g. *Paleosuchus* spp., the third premaxillary tooth is the largest. However, scoring it like this would only be right if the lost tooth would have been the fifth or fourth one, but if the missing tooth would have been one of the first three teeth, the scoring would be incorrect. We therefore decided it would be safest to score the taxa with only four teeth in the premaxilla as (?), until it could be clarified which tooth was missing.

Further rescorings were made for the following taxa:

*Brachychampsa montana* was previously scored with the third and fourth tooth as the largest (190-3). All of the premaxillary teeth of *B. montana* are relatively similar in size with the exception of the slightly larger third: “The premaxillary teeth are nearly equal in size, but the 3rd is slightly larger and the 5^th^ slightly smaller than the others.” (Norell, Clark & Hutchison, 1994, p.16 & fig.5). We therefore changed the scoring to state (1) for this analysis.

*Alligator mississippiensis* was previously scored as having the first four teeth equal in size (190-6). Comparison with specimens SZ 44, SZ 40d18, SZ 1057 however, shows that the fourth premaxilla tooth is the largest in *A. mississippiensis* as in other *Alligator* spp. Therefore, we changed the scoring to (2). This was likely a typo in Li, Wu & Rufolo (2019).

*Melanosuchus niger* was previously scored as the third and fourth premaxillary tooth being the largest. Vieira et al. (2016 fig.3) however clearly shows that in *M. niger* the fourth one is the largest premaxillary tooth. Therefore, we changed the scoring to (2).

**List of common synapomorphies for major clades**

*Pietraroiasuchus ormezzanoi* + *Shamosuchus djadochtaensis* + *Acynodon iberoccitanus* + *Acynodon adriaticus* + *Hylaeochampsa vectiana* + *Iharkutosuchus makadii*: **196** (1)

Crocodylia: **61** (0), **71** (1), **121** (1), **158** (1), **199** (2)

Gavialoidea: **49** (2), **50** (2), **54** (3), **81** (1), **90** (1), **92** (2), **116** (1), **133** (0), **147** (1), **151** (0), **197** (1)

*Lohuecosuchus mechinorum* + *Lohuecosuchus megadontos* + *Arenysuchus gascabadiolorum* + *Allodapusuchus subjuniperus* + *Allodaposuchus precedens*: **128** (1), **137** (1), **148** (0), **149** (0), **152** (1), **153** (0), **177** (1)

*Borealosuchus* spp. (5 taxa): **11** (1), **29** (0), **36** (1), **47** (0), **94** (1)

*Planocrania* spp. + *Boverisuchus* spp. (4 taxa): **79** (2), **151** (0)

Brevirostres: **18** (0), **27** (1), **33** (1), **119** (1), **149** (2)

Crocodyloidea: **115** (1), **181** (3), **199** (3)

Crocodylinae: **54** (1), **197** (1)

Tomistominae: **115** (0), **116** (1), **118** (0), **128** (1), **137** (1)

Alligatoroidea: **1** (1), **6** (1), **47** (0), **61** (1), **70** (1), **72** (1), **91** (1), **104** (1), **131** (1), **141** (1), **177** (1)

Diplocynodontinae: **15** (0), **42** (2), **140** (1)

Globidonta: **69** (0), **151** (0), **195** (0)

Alligatoridae: **26** (1), **39** (1)

Alligatorinae: **40** (2), **86** (1), **130** (1)

Caimaninae: **57** (1), **66** (1), **72** (0), **160** (3)

Orientalosuchina: **49** (0)

**List of specimens cited in this study:**

(Note: Taxa in bold were scored based on personal observation or additional photographic material besides of what is available in published works.)

*Acynodon adriaticus* (MCSNT 57248)

*Acynodon iberoccitanus* (ACAP-FX1, ACAP-M1343, ACAP-QR7)

*Alligator mcgrewi* (AMNH 7303)

***Alligator mississippiensis*** (IRSBN 19.664 5.858, **SZ 44, SZ 40d18, SZ 48b7, SZ 1057**)

*Alligator olseni* (MCZ 1887, UF 176192)

*Alligator prenasalis* (SDSM 243, YPM VPPU 014063)

***Alligator sinensis*** (IRSNB 13904-3487, **SMNS 4915**, USNM 292078, **ZNS 54/969**)

*Allodaposuchus precedens* (MMS/VBN-12-10A, MMS/VBN-12-10D, MMS/VBN-12-42, MMS/VBN-93-28, PSMUBB V 438)

*Allodaposuchus subjuniperus* (MPZ 2012/288)

*Allognathosuchus polyodon* (AMNH 6049)

*Allognathosuchus wartheni* (YPM-PU 16989)

***Arambourgia gaudryi*** (MNHN QU17155)

***Asiatosuchus germanicus*** (GMH XIV-4757a-1956, GMH XXXV- 477-1963)

*Asiatosuchus nanlingensis* (IVPP V 2773)

*Baryphracta deponiae* (IRSNB R 261, IRSNB R 262)

*Bottosaurus harlani* (NJSM 11265)

*Boverisuchus magnifrons* (GMH LEO X 8001, GMH XVIII 3094, GMH XXXVI 274)

*Boverisuchus vorax* (FMNH PR399)

*Brachychampsa montana* (AMNH-DVP 5032, UCMP 133901)

*Brachychampsa sealey* (NMMNH P-25050)

***Caiman crocodilus*** (FMNH 73441, **SZ 10276**, TMM m-7365)

***Caiman latirostris*** (BMNH 94-3-22-6, **SZ 48b5**)

*Ceratosuchus burdoshi* (FMNH P15576, UMMP 68238, UMMP 71490)

*Crocodylus depressifrons* (IRSNB R251, IRSNB R252, MNHN F BR 13194, MNHN G 160)

***Crocodylus niloticus*** (**SZ 381, SZ 9486**)

***Crocodylus porosus*** (**SZ 4120, SZ 18-1**)

***Diplocynodon darwini*** (**GMH Leo II- 9000 -1932, GMH 6026, GMH 6077, GMH XXII-588 -1965, GMH XXII-700 -1965**)

*Diplocynodon hantoniensis* (BMNH 30392)

*Diplocynodon muelleri* (NMB Spa 4)

*Diplocynodon ratelii* (MNHN SG12853, MNHN sg557)

*Diplocynodon remensis* (MHNH F BR 4020, CE0001)

*Eoalligator chunyii* (IVPP V 2716, IVPP V 2721)

***Gavialis gangeticus*** (**SZ 7458, SZ 38K2, SZ 2b2 (Rept 53), SZ Rept 180, SZ Rept 40**)

***Hassiacosuchus haupti*** (**GMH Ce IV-5900 -1932, GMH CE IV-6042, HLMD-Me 1435, HLMD-Me4415, HLMD-Me 52, HLMD-Me 5262, HLMD-Me 6117, HLMD-Me 9119, SMF-Me 3584, SMNK 927**)

***Krabisuchus siamogallicus*** (**Kr-C-006, Kr-C-007, Kr-C-010, Kr-C-012, Kr-C-015, Kr-C-017, Krc-020, Kr-C-021, Kr-C-022, Kr-C-028, Kr-C-032, Krc-038, Kr-C-065**)

*Leydiosuchus canadensis* (NMC 8543, NMC 8942, ROM 1903)

Maoming alligatoroid (MMC 001)

*Melanosuchus niger* (FMNH 27036, UF 72914)

*Navajosuchus mooki* (AMNH 6780)

*Orthogenysuchus olseni* (AMNH 5178)

*Osteolaemus osborni* (AMNH 10083)

***Osteolaemus tetraspis*** (**SMNS 11112**)

***Paleosuchus palpebrosus*** (FMNH 69871, **SMNS 11108**)

*Paleosuchus trigonatus* (FMNH 81980, MNHN-AC 2014-1)

*Paratomistoma courti* (CGM 421 88)

*Pietraroiasuchus ormezzanoi* (INHE PC-1, INHE PC-2)

*Procaimanoidea kayi* (CM 9600)

*Pocaimanoidea utahensis* (USNM 15996, USNM 15997)

***Protoalligator huiningensis*** (IVPP V 4058)

*Stangerochampsa mccabei* (RTMP 86.61.1)

*Maomingosuchus petrolica* (DM-F0001, DM-F0005, NMNS002060-F027511)

***Tomistoma schlegelii*** **(SMNS 13023**)

*Toyotamaphimeia machikanensis* (MOU F0001)

*Voay robustus* (AMNH 3101, MCZ 1006)

**Institutional Abbreviations**

(Note: Only the bold institutions were visited in person)

ACAP: Association Culturelle, Archéologique et Paléontologique de l’Ouest Biterrois, Cruzy, Hérault, France;

AMNH: American Museum of Natural History, New York, USA;

BMNH: Natural History Museum, London, United Kingdom;

CE: Collection Eldonia, Gannat, France;

CM: Carnegie Museum of Natural History, Pittsburgh, Pennsylvania, USA;

CGM: Cairo Geological Museum, Cairo, Egypt;

DM: Darwin Museum, Keelung, Taiwan;

FMNH: Field Museum of Natural History, Chicago, Illinois, USA;

**GMH**: Geiseltalmuseum of Martin-Luther-University Halle-Wittenberg, Germany;

**GPIT**: Geologisch-Paläontologisches Institut Tübingen, Germany;

**HLMD**: Hessisches Landesmuseum Darmstadt, Germany;

INHE: Instituto de Historia Natural y Ecología, Tuxtla Gutierrez, México;

IRSNB: Institut royal des Sciences naturelles de Belgique, Belgium;

IVPP: Institute of Vertebrate Paleontology and Paleoanthropology, Chinese Academy of Sciences, Beijing, China;

KR: Krabi crocodilian, Sirindhorn Museum, Kalasin Province, Thailand;

MCSNT: Museo Civico di Storia Naturale di Trieste, Italy;

MCZ: Museum of Comparative Zoology, Harvard University, Cambridge, Massachusetts, USA;

MMC: collection of crocodylians from the Maoming Basin in the School of Life Sciences, Sun Yat-sen University, Guangzhou, China;

MMS/VBN: Musée du Moulin seigneurial/Velaux-La Bastide Neuve, France;

MNHM: Museum National d'Histoire Naturelle, Paris, France;

MOU: Museum of Osaka University, Japan;

MPZ: Museo Paleontolo´gico de la Universidad de Zaragoza, Spain;

NJSM: New Jersey State Museum, Trenton, New Jersey, USA;

NMB: Naturhistorisches Museum Basel, Switzerland;

NMC: Canadian Museum of Nature, Ottawa, Ontario, Canada;

PSMUBB: Paleontology-Stratigraphy Museum, University Babes¸ Bolyai, Cluj-Napoca, Romania;

ROM: Royal Ontario Museum, Toronto, Ontario, Canada;

RTMP: Royal Tyrrell Museum of Palaeontology, Drumheller, Canada;

**SMF**: Senckenberg Museum, Frankfurt, Germany;

**SMNK**: Staatliches Museum für Naturkunde, Karlsruhe, Germany;

**SMNS**: Staatliches Museum für Naturkunde Stuttgart, Germany;

SMU: South Methodist University, Dallas, USA;

**SZ**: Museum der Universität Tübingen, Zoologische Schausammlung, Tübingen, Germany;

TMM: Texas Memorial Museum, University of Texas at Austin, Austin, Texas, USA;

UCMP: University of California Museum of Paleontology, California, USA;

UF: Florida Museum of Natural History, Gainesville, Florida, USA;

UMMP: University of Michigan Museum of Paleontology, Ann Arbor, Michigan, USA;

USMN: United States National Museum, Smithsonian Institution, Washington, DC, USA;

YPM: Peabody Museum of Natural History, Yale University, New Haven, Connecticut;

**ZNS**: Zentralmagazin Naturwissenschaftlicher Sammlung, Halle (Saale), Germany

# References

**Bartels, W. S. (1983).** A transitional Paleocene-Eocene reptile fauna from the Bighorn Basin, Wyoming. *Herpetologica*, 359-374.

**Brochu, C. A. (1999).** Phylogenetics, taxonomy, and historical biogeography of Alligatoroidea. *Journal of Vertebrate Paleontology*, *19*(S2), 9-100.

**Brochu, C. A. (2004).** Alligatorine phylogeny and the status of *Allognathosuchus* Mook, 1921. *Journal of Vertebrate Paleontology*, *24*(4), 857-873.

**Brochu, C. A. (2007) (b).** Morphology, relationships, and biogeographical significance of an extinct horned crocodile (Crocodylia, Crocodylidae) from the Quaternary of Madagascar. *Zoological Journal of the Linnean Society*, *150*(4), 835-863.

**Brochu, C. A. (2011).** Phylogenetic relationships of *Necrosuchus ionensis* Simpson, 1937 and the early history of caimanines. *Zoological journal of the linnean society*, *163*(suppl_1), S228-S256.

**Brochu, C. A., & Storrs, G. W. (2012).** A giant crocodile from the Plio-Pleistocene of Kenya, the phylogenetic relationships of Neogene African crocodylines, and the antiquity of *Crocodylus* in Africa. *Journal of Vertebrate Paleontology*, *32*(3), 587-602.

**Case, E. C. (1925).** *Note on a new species of the Eocene crocodilian Allognathosuchus, A. wartheni*. University of Michigan. *Contrib. Mus. Geol. Univ. Mich.* 2: 93-97.

**Conrad, J. L., Jenkins, K., Lehmann, T., Manthi, F. K., Peppe, D. J., Nightingale, S., Cossette, A., Dunsworth, H. M., Harcourt-Smith, W. E. H., & Mcnulty, K. P. (2013).** New specimens of ‘*Crocodylus*’ *pigotti* (Crocodylidae) from Rusinga Island, Kenya, and generic reallocation of the species. *Journal of Vertebrate Paleontology*, *33*(3), 629-646.

**Cope, E. D. (1861).** List of the recent species of emydosaurian reptiles in the Museum of the Academy of Natural Sciences. *Proceedings of the Academy of Natural Sciences of Philadelphia*, *12*, 549-550.

**Cope, E. D. (1872).** On the Extinct Vertebrata of the Eocene of Wyoming: Observed by the Expedition of 1872, with Notes on the Geology. *US Government Printing Office.*

**Cossette, A. P., & Brochu, C. A. (2018).** A new specimen of the alligatoroid *Bottosaurus harlani* and the early history of character evolution in alligatorids. *Journal of Vertebrate Paleontology*, 1-22.

**Cuvier, F. G. (1807).** Sur les diffèrentes espèces de Crocodiles vivans et Sur leurs caractères distinctiss. *Annales du Museum d’Histoire Naturelle de Paris* 10:8–66.

**Daudin, F. M. (1801).** *Histoire naturelle, générale et particulière des reptiles: ouvrage faisant suite à l'histoire naturelle générale et particulieère, composée par Leclerc de Buffon, et rédigée par CS Sonnini, membre de plusieurs sociétés savantes* (Vol. 3). Dufart.

**Daudin, F.M. (1802).** *Histoire naturelle, générale et particulière des Reptiles*. De L’Imprimerie de F. Dufart, Paris, 452 p.

**Delfino, M., Martin, J. E., & Buffetaut, E. (2008).** A new species of *Acynodon* (Crocodylia) from the upper cretaceous (Santonian–Campanian) of Villaggio del Pescatore, Italy. *Palaeontology*, *51*(5), 1091-1106.

**Fauvel, A.A., (1879).** Alligators in China. J. *North-China Branch Roy. Asiatic Soc.* 13, 1–36.

**Gilmore, C. W. (1911).** A new fossil alligator from the Hell Creek beds of Montana. *Proceedings of the United States National Museum*, 41: 297-301.

**Gilmore, C. W. (1946).** A new crocodilian from the Eocene of Utah. *Journal of Paleontology*, 62-67.

**Gmelin, J. (1789).** Linnei Systema Naturae.-1057 p. *Leipzig (GE Beer)*.

**Grandidier A, Vaillant L. (1872).** Sur le crocodile fossile d’Amboulintsatre (Madagascar). *Comptes Rendus de l’Academie* *des Sciences de Paris* 75**:** 150–151.

**Kälin, J. A. (1939).** Ein extrem kurzschnauziger Crocodilide aus den Phosphoriten des Quercy, *Arambourgia* (nov. gen.) *gaudryi* de Stefano. Abhandlungen der Schweizerischen Palaeontologischen Gesellschaft 62:1-18.

**Kuhn, O. (1938).** Die Crocodilier aus dem mittlerern Eozän des Geiseltales bei Halle. *Nova Acta Leopoldina* 39, 313–28.

**Lambe, L. M. (1907).** On a new crocodilian genus and species from the Judith River Formation of Alberta. *Transactions of the Royal Society of Canada*, *4*, 219-244.

**Li, C., Wu, X. C., & Rufolo, S. J. (2019).** A new crocodyloid (Eusuchia: Crocodylia) from the Upper Cretaceous of China. *Cretaceous Research*, *94*, 25-39.

**Linnaeus C. (1758).** *Systema naturæ per regna tria naturæ, secundum classes, ordines, genera, species, cum characteribus, differentiis, syninymis, locis. Tomus I. Editio decima, reformata.* Stockholm: Laurentius salvius

**Loomis, F. B. (1904).** ART. XLIII.--Two New River Reptiles from the Titanothere Beds. *American Journal of Science (1880-1910)*, *18*(108), 427-432

**Lucas, S. G., & Estep, J. W. (2000).** Osteology of *Allognathosuchus mooki* Simpson, a Paleocene Crocodilian from the San Juan Basin, New Mexico, and the monophyly of *Allognathosuchus*. *New Mexico's Fossil Record 2: Bulletin 16*, *16*, 155.

**Martin, J. E., & Lauprasert, K. (2010).** A new primitive alligatorine from the Eocene of Thailand: relevance of Asiatic members to the radiation of the group. *Zoological Journal of the Linnean Society*, *158*(3), 608-628.

**Meyer, H. von. (1832).** Paleologica zur Geschichte der Erde und ihrer Geschöpfe. S. Schmerber, Frankurt-am-Main, 560 pp.

**Mook, C. C. (1941).** A new crocodilian, *Hassiacosuchus kayi*, from the Bridger Eocene beds of Wyoming. *Annals of Carnegie Museum*, *28*, 207-220.

**Norell, M., Clark, J. M., & Hutchison, J. H. (1994).** The Late Cretaceous alligatoroid *Brachychampsa montana* (Crocodylia): new material and putative relationships. American Museum novitates; no. 3116.

**Salisbury, S. W., Molnar, R. E., Frey, E., & Willis, P. M. (2006).** The origin of modern crocodyliforms: new evidence from the Cretaceous of Australia. *Proceedings of the Royal Society of London B: Biological Sciences*, *273*(1600), 2439-2448.

**Schmidt KP. (1938).** New crocodilians from the upper Paleocene of western Colorado. *Field Museum of Natural History Geological Series* 6: 315–321.

**Schmidt, K. P. (1941).** A new fossil alligator from Nebraska. *Fieldiana: Geology* 8:27-32.

**Simpson, G. G. (1930).** *Allognathosuchus mooki*, a new crocodile from the Puerco Formation. *American Museum Novitates* 445:1-16.

**Skutschas, P. P., Danilov, I. G., Kodrul, T. M., & Jin, J. (2014).** The first discovery of an alligatorid (Crocodylia, Alligatoroidea, Alligatoridae) in the Eocene of China. *Journal of Vertebrate Paleontology*, *34*(2), 471-476.

**De Stefano, G. (1905).** Appunti sui Batraci e rettili del Quercy appartenenti alla collezioni Rossignol. *Bolletino della Societa Geologia Italiana*, *24*, 17-67.

**Tchernov, E., & Van Couvering, Judith (1978).** New crocodiles from the Early Miocene of Kenya. *Palaeontology*, *21*(Part 4), 857-867

**Vieira, L. G., Santos, A. L., Lima, F. C., Mendonça, S. H., Menezes, L. T., & Sebben, A. (2016).** Osteology of *Melanosuchus niger* (Crocodylia: Alligatoridae) and the evolutionary evidence. *Pesquisa Veterinária Brasileira*, *36*(10), 1025-1044.

**Wang, Y. Y., Sullivan, C., & Liu, J. (2016).** Taxonomic revision of *Eoalligator* (Crocodylia, Brevirostres) and the paleogeographic origins of the Chinese alligatoroids. *PeerJ*, *4*, e2356.

**Wassersug, R. J., & Hecht, M. K. (1967).** The status of the crocodylid genera *Procaimanoidea* and *Hassiacosuchus* in the New World. *Herpetologica*, *23*(1), 30-34.

**Weitzel, K. (1935).** *Hassiacosuchus haupti* ngn sp., ein durophages Krokodil aus dem Mitteleozän von Messel. *Notizblatt des Vereins für Erdkunde und der Hessischen Geologischen Landesanstalt Darmstadt* 16:40_49.

**White, T. E. (1942).** A new alligator from the Miocene of Florida. *Copeia*, *1942*(1), 3-7.

**Williamson, T. E. (1996).** ? *Brachychampsa sealeyi*, sp nov.,(Crocodylia, Alligatoroidea) from the Upper Cretaceous (lower Campanian) Menefee Formation, northwestern New Mexico. *Journal of Vertebrate Paleontology*, *16*(3), 421-431.

**Wu, X. C., Brinkman, D. B., & Russell, A. P. (1996).** A new alligator from the Upper Cretaceous of Canada and the relationship of early eusuchians. *Palaeontology*, *39*, 351-376.

**Young, C. C. (1964).** New fossil crocodiles from China. *Vertebrata Palasiatica*, *8*(2), 189-208.

**Young, C. C. (1982).** A Cenozoic crocodile from Huaining, Anhui. *Selected Works of Yang Zhongjian. China: Academia Sinica*, 47-48.
