# Supplementary material for: A new alligatoroid from the Eocene of Vietnam highlights an extinct Asian clade independent from extant Alligator sinensis
Source: PeerJ. 2019 Nov 5;7:e7562. doi: 10.7717/peerj.7562 (PMC6839522; doi:10.7717/peerj.7562)
Supplement: Supplemental Information 4 — Postcranial measurements. [file peerj-07-7562-s004.docx]

# Supplementary 4

**Postcranial Measurements**

Weathered bones are marked with an asterisk (*).Measurements (in mm)

Abbreviations:

CL: centrum length;

CW: centrum width (measured at the anterior end of the body);

CH: centrum height (measured at the anterior end of the body);

AIVAS: anteroposterior depth of concave anterior intervertebral articular surface

PIVAS: anteroposterior height of convex posterior intervertebral articular surface (measured from the ventral part of the condyle);

CoH: dorsoventral height of the condyle of the posterior intervertebral articular surface in posterior view;

CoW: transverse width of the condyle of the posterior intervertebral articular surface in posterior view

**Vertebrae**

|  | Length | Wide | Height |
| --- | --- | --- | --- |
| Atlas | 10.7 | 14.5 | 4.5 |

Cervical vertebrae

|  | CL | CW | CH | AIVAS | PIVAS | CoH | CoW |
| --- | --- | --- | --- | --- | --- | --- | --- |
| Axis* | 22.3 | - | 13.9 | - | 3.2 | 11.2 | 10.9 |
| C 1 | 22.9 | 12.4 | 14.8 | 3.2 | 3.9 | 9.8 | 8.9 |
| C 2* | 23.1 | 12.5 | 15.3 | 3.1 | 4.2 | 9.9 | 9.6 |
| C 3 or C4 | 20.7 | 14.4 | 15.3 | 3.6 | 3.6 | 10.9 | 11 |
| C 5* | 21.8 | - | 15.5 | 3.5 | 4.4 | 10.4 | 10.3 |
| C 6 or C7 | 21.7 | 14.8 | 15.4 | 3.3 | 4.8 | 10.6 | 10.2 |
| C ?* | - | - | - | 3.9 | - | - | - |
| C ?* | - | 14.2 | 14.1 | 3.9 | - | - | - |

Dorsal vertebrae

|  | CL | CW | CH | AIVAS | PIVAS | CoH | CoW |
| --- | --- | --- | --- | --- | --- | --- | --- |
| D 1 | 28.6 | 14.7 | 14.9 | 4.9 | 7.4 | 9.9 | 12.6 |
| D 2 | 25.8 | 15.5 | 13.8 | 3.4 | 3.9 | 8.7 | 12.7 |
| D 3* | 28.7 | 16.9 | 15.6 | 4.6 | 4.9 | 12.4 | 11.2 |
| D 4* | - | - | - | - | - | - | - |
| D 5* | 28.3 | 15.8 | - | - | - | - | - |
| D 6* | - | 14.8 | 14.2 | - | 5.3 | 11.4 | 10.4 |
| D 7* | 31.8 | 16.6 | 15.7 | 4.7 | 6.2 | 12.5 | 12.7 |
| D 8* | - | - | 13.8 | - | - | - | - |
| D 9* | - | - | - | - | - | - | - |
| D 10* | - | 17.1 | 14.3 | 3.9 | 4.8 | 11.1 | 12.1 |

Sacral vertebra

|  | CL | CW | CH | AIVAS | PIVAS | CoH | CoW |
| --- | --- | --- | --- | --- | --- | --- | --- |
| S 1* | 20.1 | 17.5 | 13.9 | 3.3 | - | - | - |

Caudal vertebrae

|  | CL | CW | CH | AIVAS | PIVAS | CoH | CoW |
| --- | --- | --- | --- | --- | --- | --- | --- |
| C 1 | 25.6 | 12.6 | 13.4 | - | 3.4 | 8.9 | 9.6 |
| C 2* | 27.4 | 14.3 | 13.5 | 2.9 | 3.8 | 7.3 | 8 |
| C 3 | 26.5 | 11.8 | 11.6 | 1.8 | 2.6 | 5.2 | 6.2 |
| C 4* | 26.9 | 13.5 | 10.6 | 2.3 | 2.9 | 5.4 | 6.5 |
| C 5* | 26.3 | 11.9 | 13 | - | 3.7 | 7.2 | 7.2 |
| C 6* | 28.9 | 10.8 | 10.4 | 1.7 | 2.8 | 4.2 | 5.1 |
| C 7* | 25.2 | 12.8 | 11.2 | 1.6 | 2.1 | 4.5 | - |
| C 8* | 20.7 | 4.4 | 5.7 | 1.9 | 1.3 | 3.5 | 3.9 |

**Ribs**

|  | Length | Width | Height |
| --- | --- | --- | --- |
| Cervical rib 1* | - | 10.2 | 15.3 |
| Cervical rib 2 | 28.1 | - | 14.5 |

|  | Length | Width at dorsal | Width at ventral |
| --- | --- | --- | --- |
| Dorsal rib | 68.9** | 11.4 | 10.1 |

**length without the articulation surface with the parapophysis of the vertebra

**Pectoral girdle and forelimb**

|  | Length | Width at prox. end | Width at dist. end |
| --- | --- | --- | --- |
| Scapula* (right) | 62.4 | - | - |
| Coracoid (right) | 52.1 | 34 | 33.2 |
| Humerus (left) | - | - | 21.1 |
| Humerus* (right) | - | 25.2 | - |
| Ulna | - | - | 12.5 |
| Radius* | - | 12.9 | 13.9 |
| Radiale* | 21.7 | 12.7 | 8.8 |
| Metacarpalia* | 23.7 | 9.1 | 6.2 |
| Ph 1 | 14.8 | 7.6 | 6.7 |
| Ph 2* | 20.8 | - | - |
| Ph 3* | - | 9.2 | - |
| Ph 4* | - | - | - |
| Ph 5* | - | - | - |
| Ph 6* | - | - | - |
| Ph 7* | - | - | - |

**Pelvic girdle**

|  | Height | Max. width | Width at artic. side |
| --- | --- | --- | --- |
| Ilium* (right) | 35.8 | 49.9 | 36.5 |
| Ilium* (left) | 38.4 | - | - |
| Ischium* (left) | - | 30.2 | 30.2 |

**Hindlimb**

|  | Length | Width at prox. end | Width at dist. end |
| --- | --- | --- | --- |
| Femur (right) | 112.8 | 23.8 | 23.9 |
| Femur* (left) | - | - | 27.3 |
| Tibia* | 75.9 | - | 17.2 |
| Fibula* (right) | 79.4 | 11.0 | 10.1 |
| Fibula* (left) | - | 8.8 | - |
| Mt 1* | 36.4 | 12.0 | 8.1 |
| Mt 2* | - | 13.6 | - |
| Mt 3* | - | 12.7 | - |

**Claws**

|  | Length | Width | Height |
| --- | --- | --- | --- |
| Claw 1 | 28.3 | 6.3 | 4.7 |
| Claw 2 | 27.3 | 5.1 | 5.6 |
| Claw 3 | 16.9 | 4.2 | 3.5 |
| Claw 4 | 13.0 | 3.3 | 3.7 |
